# Epigenome-wide association study of lung function level and its change

**DOI:** 10.1183/13993003.00457-2019

**Published:** 2019-07-04

**Authors:** Medea Imboden, Matthias Wielscher, Faisal I. Rezwan, André F.S. Amaral, Emmanuel Schaffner, Ayoung Jeong, Anna Beckmeyer-Borowko, Sarah E. Harris, John M. Starr, Ian J. Deary, Claudia Flexeder, Melanie Waldenberger, Annette Peters, Holger Schulz, Su Chen, Shadia Khan Sunny, Wilfried J.J. Karmaus, Yu Jiang, Gertraud Erhart, Florian Kronenberg, Ryan Arathimos, Gemma C. Sharp, Alexander John Henderson, Yu Fu, Päivi Piirilä, Kirsi H. Pietiläinen, Miina Ollikainen, Asa Johansson, Ulf Gyllensten, Maaike de Vries, Diana A. van der Plaat, Kim de Jong, H. Marike Boezen, Ian P. Hall, Martin D. Tobin, Marjo-Riitta Jarvelin, John W. Holloway, Deborah Jarvis, Nicole M. Probst-Hensch

**Affiliations:** 1 Chronic Disease Epidemiology Unit, Dept of Epidemiology and Public Health, Swiss Tropical and Public Health Institute, Basel, Switzerland; 2 University of Basel, Basel, Switzerland; 3 MRC-PHE Centre for Environment and Health, Imperial College London, London, UK; 4 Dept of Epidemiology and Biostatistics, School of Public Health, Imperial College London, London, UK; 5 Human Development and Health, Faculty of Medicine, University of Southampton, Southampton, UK; 6 Population Health and Occupational Disease, NHLI, Imperial College London, London, UK; 7 Centre for Cognitive Ageing and Cognitive Epidemiology, University of Edinburgh, Edinburgh, UK; 8 Medical Genetics Section, University of Edinburgh Centre for Genomic and Experimental Medicine and MRC Institute of Genetics and Molecular Medicine, Western General Hospital, Edinburgh, UK; 9 Alzheimer Scotland Dementia Research Centre, University of Edinburgh, Edinburgh, UK; 10 Dept of Psychology, University of Edinburgh, Edinburgh, UK; 11 Institute of Epidemiology, Helmholtz Zentrum München, German Research Center for Environmental Health, Neuherberg, Germany; 12 Research Unit Molecular Epidemiology, Helmholtz Zentrum München, German Research Center for Environmental Health, Neuherberg, Germany; 13 Comprehensive Pneumology Center Munich (CPC-M), Member of the German Center for Lung Research (DZL), Munich, Germany; 14 Dept of Mathematical Sciences, University of Memphis, Memphis, TN, USA; 15 Division of Epidemiology, Biostatistics, and Environmental Health, School of Public Health, University of Memphis, Memphis, TN, USA; 16 Division of Genetic Epidemiology, Dept of Medical Genetics, Molecular and Clinical Pharmacology, Medical University of Innsbruck, Innsbruck, Austria; 17 MRC Integrative Epidemiology Unit, University of Bristol, Bristol, UK; 18 Dept of Population Health Sciences, Bristol Medical School, University of Bristol, Bristol, UK; 19 Bristol Dental School, University of Bristol, Bristol, UK; 20 Institute for Molecular Medicine Finland (FIMM), University of Helsinki, Helsinki, Finland; 21 Unit of Clinical Physiology, HUS Medical Imaging Center, Helsinki University Central Hospital and University of Helsinki, Helsinki, Finland; 22 Obesity Research Unit, Research Programs Unit, University of Helsinki, Helsinki, Finland; 23 Abdominal Center, Endocrinology, Helsinki University Hospital and University of Helsinki, Helsinki, Finland; 24 Dept of Immunology, Genetics and Pathology, Science for Life Laboratory, Uppsala University, Uppsala, Sweden; 25 University of Groningen, University Medical Center Groningen, Dept of Epidemiology, Groningen, The Netherlands; 26 Groningen Research Institute for Asthma and COPD (GRIAC), University of Groningen, University Medical Center Groningen, Groningen, The Netherlands; 27 Division of Respiratory Medicine, University of Nottingham, Nottingham, UK; 28 National Institute for Health Research, Nottingham Biomedical Research Centre, Nottingham University Hospitals, Nottingham, UK; 29 Dept of Health Sciences, University of Leicester, Leicester, UK; 30 National Institute of Health Research Biomedical Research Centre, University of Leicester, Leicester, UK; 31 Center for Life Course Health Research, Faculty of Medicine, University of Oulu, Oulu, Finland; 32 Biocenter Oulu, University of Oulu, Oulu, Finland; 33 Unit of Primary Health Care, Oulu University Hospital, Oulu, Finland; 34 Dept of Life Sciences, College of Health and Life Sciences, Brunel University London, London, UK; 35 These authors contributed equally to this work

## Abstract

Previous reports link differential DNA methylation (DNAme) to environmental exposures that are associated with lung function. Direct evidence on lung function DNAme is, however, limited. We undertook an agnostic epigenome-wide association study (EWAS) on pre-bronchodilation lung function and its change in adults.

In a discovery–replication EWAS design, DNAme in blood and spirometry were measured twice, 6–15 years apart, in the same participants of three adult population-based discovery cohorts (n=2043). Associated DNAme markers (p<5×10^−7^) were tested in seven replication cohorts (adult: n=3327; childhood: n=420). Technical bias-adjusted residuals of a regression of the normalised absolute β-values on control probe-derived principle components were regressed on level and change of forced expiratory volume in 1 s (FEV_1_), forced vital capacity (FVC) and their ratio (FEV_1_/FVC) in the covariate-adjusted discovery EWAS. Inverse-variance-weighted meta-analyses were performed on results from discovery and replication samples in all participants and never-smokers.

EWAS signals were enriched for smoking-related DNAme. We replicated 57 lung function DNAme markers in adult, but not childhood samples, all previously associated with smoking. Markers not previously associated with smoking failed replication. cg05575921 (*AHRR* (aryl hydrocarbon receptor repressor)) showed the statistically most significant association with cross-sectional lung function (FEV_1_/FVC: p_discovery_=3.96×10^−21^ and p_combined_=7.22×10^−50^). A score combining 10 DNAme markers previously reported to mediate the effect of smoking on lung function was associated with lung function (FEV_1_/FVC: p=2.65×10^−20^).

Our results reveal that lung function-associated methylation signals in adults are predominantly smoking related, and possibly of clinical utility in identifying poor lung function and accelerated decline. Larger studies with more repeat time-points are needed to identify lung function DNAme in never-smokers and in children.

## Abstract 2

**An agnostic association study on lung function using longitudinal population-based cohort data shows that differentially methylated genomic sites related to smoking are strongly associated with lung function in adults** http://ow.ly/wYID30onUB4

## Introduction

Lung function has an estimated heritability of between 30% and 70% [1]. The variance in phenotype remains incompletely explained by genetic variation, but the impact of environmental exposure on respiratory health and lung function over the life course is well recognised. In particular, pro-inflammatory and oxidative inhalants such as cigarette and environmental tobacco smoke, air pollution, and occupational exposures are important contributors to the increased risk of respiratory symptoms, accelerated lung function decline in adults and poor lung growth in children. DNA methylation (DNAme) has been associated with a wide variety of traits and chronic diseases.

A large body of evidence including results from epigenome-wide association studies (EWASs) shows differentially methylated CpG (5′-cytosine-phosphate-guanine-3′ dinucleotide) sites throughout the genome in response to environmental exposures, in particular cigarette smoking [2–4]. In contrast, reports of DNAme associated with respiratory diseases and lung function show inconsistent findings [5, 6]. Most recently, however, independent reports pointed to the consistent association of DNAme in the *AHRR* gene, cg05575921, with lung function in adults [4, 6, 7].

The current study aimed at agnostically identifying lung function-specific DNAme signals. We undertook a covariate-adjusted EWAS using questionnaire data, spirometry and peripheral blood samples collected in the same participants (discovery cohorts: ECRHS (European Community Respiratory Health Study), NFBC1966 (Northern Finland Birth Cohort 1966) and SAPALDIA (Swiss Study on Air Pollution Heart and Lung Disease in Adults); cohort description in supplementary material) at two time-points 6–15 years apart. EWAS analyses were performed on lung function parameters of forced expiratory volume in 1 s (FEV_1_), forced vital capacity (FVC) and their ratio (FEV_1_/FVC). The analyses focused on cross-sectional associations at different time-points and on identifying DNAme markers predicting change in lung function. We tested discovery-identified CpGs (p<5×10^−7^ for at least one lung function parameter) for replication in adult samples from five adult cohorts (LBC1936 (Lothian Birth Cohort 1936, adult inception birth cohort), KORA (Cooperative Health Research in the Augsburg Region Study), LifeLines (LifeLines cohort study), NSPHS (North Sweden Population Health Study) and FTC (Finnish Twin Cohort study)) and in childhood samples from two birth cohorts (ALSPAC (Avon Longitudinal Study of Parents and Children) and IOWBC (Isle of Wight Birth Cohort)).

## Methods

### Study design and participants

The discovery sample (n=2043) comprised three population-based cohort studies, part of the Aging Lungs in European Cohorts (ALEC) project. ECRHS (n=470) and SAPALDIA (n=962) are adult cohorts designed to investigate respiratory health. NFBC1966 (n=611) is a birth cohort with follow-up to adult age. The replication sample consisted of five adult cohorts (KORA (n=628), LifeLines (n=1622), NSPHS (n=535), LBC1936 (n=449) and FTC (n=93)) and two childhood birth cohorts (ALSPAC (n=258) and IOWBC (n=162)). Replication data from two time-points were available only for KORA and LBC1936 (adult) and ALSPAC and IOWBC (childhood). For cohort details and contribution to analysis, refer to the supplementary material and supplementary figure S1.

All cohorts comply with the Declaration of Helsinki, and ethical approval was obtained from the respective national and regional ethical review committees.

### Procedures

In the discovery cohorts, DNAme measurements using Infinium technology (Illumina, San Diego, CA, USA) were obtained from peripheral blood samples collected at two consecutive follow-up surveys several years apart. The Infinium 450K BeadChip was used for samples of 984 SAPALDIA participants from both time-points and of 732 NFBC1966 participants collected at time-point 1. The Infinium EPIC BeadChip was used for samples of 509 ECRHS participants from both time-points and of 716 NFBC1966 participants collected at time-point 2. For cohort-specific EWAS analyses, we used all autosomal markers available for each time-point and cohort-specific EWAS marker results were meta-analysed without restriction to markers common to both arrays. DNAme data used for replication were restricted to discovery-identified (sentinel) CpGs and analysed on various arrays.

Epidemiological data, including covariate information at the subject level, were collected by interview-assisted questionnaires and objective measures. Pre-bronchodilation spirometric data were obtained by performing American Thoracic Society/European Respiratory Society-compliant spirometry (supplementary material).

### Statistical analyses

Epigenome-wide methylation data were analysed in R version 3.4.3 (R Foundation for Statistical Computing, Vienna, Austria). Differential blood cell count was estimated using a reference dataset and the R package minfi [8, 9]. DNAme used as predictors in the statistical models for the adult cohorts were obtained by deriving residuals from linear regression of the normalised absolute DNAme (β-values) on the Illumina control probe-derived 30 first principal components to correct for correlation structures within the data, including technical bias. Thus, effect sizes reported here of the association are not comparable to effect sizes reported elsewhere using normalised β-values as predictor. In the childhood data, batch effect was corrected at the analysis level by regressing the DNAme values against the technical covariates.

**FIGURE 1:**
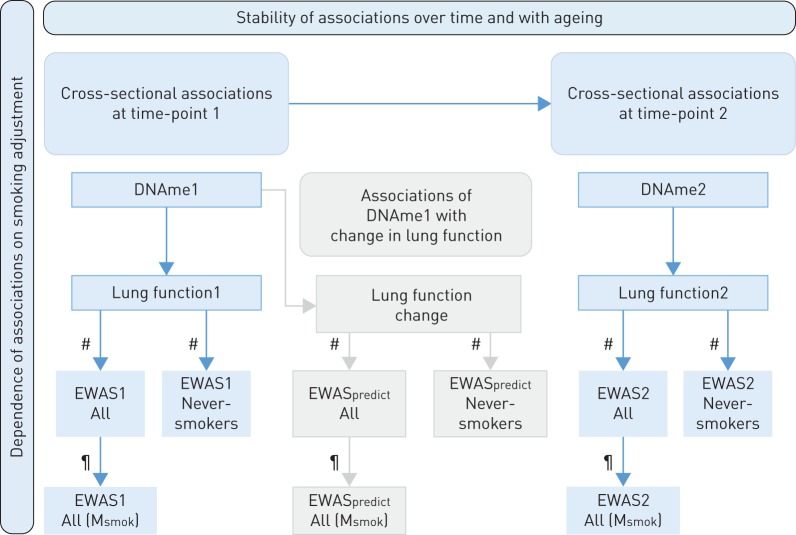
Flow of the multilevel discovery design of the epigenome-wide association study (EWAS) on lung function parameters: forced expiratory volume in 1 s (FEV_1_), forced vital capacity (FVC) and FEV_1_/FVC. DNAme1: DNA methylation at time-point 1; DNAme2: DNA methylation at time-point 2. ^#^: base model (M_base_) EWAS was covariate adjusted for age, age squared, height, squared deviation from the mean of height, sex and interaction terms of age, age squared, height and squared deviation of height with sex, education (low, medium and high), body mass index, spirometer type, study centre, and cell composition. ^¶^: smoking model EWAS (M_smok_) additionally adjusted for smoking covariates: history of smoking intensity as pack-years smoked up to the time-point of data collection for regressions and for smoking status (current smoker, ex-smoker and never-smoker). EWAS longitudinally predicting the change in lung function (EWAS_predict_) was additionally adjusted for lung function at time-point 1.

Epigenome-wide covariate-adjusted linear regression was performed to assess the association of single CpG markers with FEV_1_ (L), FVC (L), their ratio (FEV_1_/FVC) and their change during follow-up. This multilevel EWAS design tested different models in all participants and never-smoking participants (figure 1). First, cross-sectional EWASs were examined separately at time-point 1 (EWAS1) and time-point 2 (EWAS2) to assess the consistency of the association over follow-up time. Second, the association of DNAme at the first time-point (DNAme1) with change in lung function during follow-up was assessed (prediction EWAS (EWAS_predict_)). Covariate-adjusted mixed linear regressions with a random intercept on the subject were undertaken using data from both time-points (repeat cross-sectional analysis (EWAS_repeat_)).

All associations were adjusted for a set of *a priori* selected covariates known to influence respiratory outcomes from previous research conducted by SAPALDIA and ECRHS. The covariate-adjusted model (M_base_) included age, age squared, height, squared deviation from the mean of height, sex and interaction terms of sex with four covariates (age, age squared, height and squared deviation of height), education, body mass index, spirometer type, study centre as well as estimated cell composition (CD8 cells, CD4 cells, natural killer cells, B-cells, monocytes, eosinophils and neutrophils). Analyses in all participants were run without (M_base_) and with additional smoking adjustment including smoking status and pack-years (M_smok_). M_base_ covariate adjustment was applied in never-smokers. Prediction associations of DNAme1 were additionally adjusted for lung function at time-point 1. The same covariate adjustment was applied in adult replication analyses, whereas childhood covariates did not include squared terms.

Cohort-specific EWAS results were summarised by inverse-variance-weighted meta-analyses using METAL [10]. Meta-analysis results were not controlled for genomic inflation after confirming its negligible influence. Epigenome-wide significance level was set to p<1×10^−7^ (Bonferroni correction, 450 000 tests). The selection criteria for replication of sentinel CpGs was less stringent (p<5×10^−7^). Successful replication was defined as a p-value below the outcome-specific Bonferroni correction threshold.

Replicated CpGs were characterised by enrichment, pathway and functional analyses, and additional *post hoc* analyses were performed (details in supplementary material). 1) A two-sample Mendelian randomisation analysis based on publicly available data was applied to investigate the causality of replicated CpG associations. 2) A replication of a recently published mediation analysis [4] evidencing 10 smoking-related CpGs mediating the effect of smoking on lung function was undertaken in one discovery cohort (SAPALDIA). 3) To assess the combined effects of smoking-related CpGs on lung function in three discovery cohorts, we built two different DNAme smoking indices based on CpGs: a) predicting lung function effects of smoking [4] and b) located in genome-wide association study (GWAS)-identified lung function genes [2]. These smoking indices were tested for association with lung function in covariate-adjusted linear regression analyses, in all participants and in subgroups stratified by smoking status.

### Data availability statement

Statistical codes and full discovery/replication EWAS effect estimates (meta-analysed and cohort-specific) are made publically available with no end date on the public repository DRYAD (http://datadryad.org/) at the time of publication. Access restrictions apply to the individual methylome data underlying the analysis. Contact details for data requests to the contributing cohorts can be found in the supplementary material.

## Results

Differences in the cohorts' age structure and smoking habits are shown in tables 1 and 2. Mean age was highest for LBC1936 (69.9 years) and youngest for FTC (30.4 years). Self-report of current smoking status was lowest in LBC1936 (5.8%) and highest in LifeLines (43.5% due to oversampling of current smokers for the DNAme-typed subset).

**TABLE 1:** Characteristics of discovery cohorts

	**SAPALDIA 2** **time-point 1**	**SAPALDIA 3** **time-point 2**	**ECRHS II** **time-point 1**	**ECRHS III** **time-point 2**	**NFBC1966** **(age 31 years)** **time-point 1**	**NFBC1966** **(age 46 years)** **time-point 2**
**Subjects n**	962	962	470	470	611	611
**Female**	53.5	53.5	56	56	55.3	55.3
**Age years**	50.5±11.3	58.8±11.3	43.6±6.8	54.5±6.8	31.0±0.3	46.3±0.4
**Height cm**	169.4±9.2	168.7±9.4	170.0±9.2	169.2±9.3	171±8.8	171±8.9
**Weight kg**	74.2±14.7	75.5±15.4	72.6±14.6	76.2±15.5	71.3±13.6	78.7±16.3
**Body mass index kg·m^−2^**	25.8±4.4	26.5±4.6	25.0±4.0	26.5±4.4	24.2±3.7	26.7±4.8
**Smoking status**						
Never-smoker^#^	41.7	41.1	43.2	41.7	54.5	54.5
Ex-smoker	30.0	37.0	31.1	40.4	21.3	30.2
Current smoker	28.3	21.9	25.7	17.9	24.1	15.3
**Pack-years**	20.4±20.2	22.6±22.1	16.6±16.9	20.0±21.3	7.7±5.9	11.0±9.6
**Education^¶^**						
Low	5.4	5.4	11.5	11.5	0.7	0.7
Intermediate	65.7	65.7	29.2	29.2	55.9	55.9
High	28.9	28.9	59.3	59.3	43.3	43.3
**FVC L^+^**	4.4±1.0	4.1±1.1	4.3±1.0	3.9±1.0	4.8±1.0	4.5±0.9
**FEV**_**1**_ **L^+^**	3.3±0.8	3.0±0.8	3.4±0.7	3.0±0.8	4.0±0.8	3.5±0.7
**FEV**_**1**_**/FVC^+^**	0.75±0.07	0.73±0.08	0.78±0.06	0.75±0.06	0.83±0.06	0.77±0.06
**Airflow obstruction**						
FEV_1_/FVC <0.7^+^	20.4	29.5	8.9	16.2	1.8	10.8
FEV_1_/FVC <LLN^+,§^	12.9	14.1	8.7	10.4	3.3	9.5
**Doctor-diagnosed asthma**	13.8	16.5	14.3	16.8	10.7	15.8
**Respiratory medication (% missing values)**	22.2 (0.8)	23.7 (0.3)	13.4	14.2	NA	NA

Data are presented as % or mean±sd, unless otherwise stated; percentages may not total 100% due to rounding. FEV_1_: forced expiratory volume in 1 s; FVC: forced vital capacity; LLN: lower limit of normal; NA: not assessed. ^#^: self-reported lifetime nonsmoking. ^¶^: the categorical variable “education” is defined differently in cohorts (in SAPALDIA low corresponds to primary education; intermediate to secondary, middle or vocational school and high to technical college or university; in ECRHS and NFBC1966 information of age reached at end of studies is used to define low as ≤16 years, intermediate as 17–19 years and high as ≥20 years). ^+^: values derived from pre-bronchodilation spirometry (lung function values corrected for spirometer device change in SAPALDIA at time-point 2). ^§^: LLN values estimated using Global Lung Initiative 2012 reference equations [11].

**TABLE 2:** Characteristics of adult replication cohorts

	**KORA** **time-point 1**	**KORA** **time-point 2**	**LBC1936** **time-point 1**	**LBC1936** **time-point 2**	**LifeLines** **time-point 1**	**NSPHS** **time-point 1**	**FTC** **time-point 1**
**Subjects n**	628	628	449	449	1622	535	93
**Female**	53.2	53.2	46.8	46.8	42.8	53.1	47.3
**Age years**	53.6±4.5	60.1±4.5	69.6±0.9	76.3±0.7	46.7±10.8	55.1±16.0	30.4±3.8
**Height cm**	169.5±9.3	168.7±9.4	167.2±8.8	166.1±8.8	176.9±9.1	163.8±9.8	173.0±10.5
**Weight kg**	79.0±16.7	79.9±17.3	77.2±14.6	76.5±14.8	82.1±14.7	74.0±15.2	82.0±18.8
**Body mass index kg·m^−2^**	27.4±4.7	28.0±5.1	27.5±4.3	27.7±4.6	26.2±3.9	27.5±4.7	27.3±5.4
**Smoking status**							
Never-smoker^#^	38.2	38.2	52.3	52.3	56.6	83.2	53.8
Ex-smoker	43.8	45.5	40.8	41.9	0^ƒ^	NA^##^	26.9
Current smoker	18.0	16.2	6.9	5.8	43.5	16.5	19.4
**Pack-years**	12.8±19.3	13.5±20.2	13.9±24.0	14.1±24.6	21.0±11.7	8.1±21.6	NA
**Education^¶^**							
Low	47.6	47.6	49.7	49.7	23.1	NA	1.1
Intermediate	26.4	26.4	32.3	32.3	40.8	NA	38.6
High	26.0	26.0	18.0	18.0	35.4	NA	60.2
**FVC L^+^**	4.3±1.0	3.9±1.0	3.2±0.9	2.8±0.9	4.7±1.1	3.4±1.1	4.8±1.1
**FEV**_**1**_ **L^+^**	3.3±0.8	3.0±0.7	2.5±0.7	2.1±0.7	3.5±0.9	2.8±0.9	3.9±0.9
**FEV**_**1**_**/FVC^+^**	0.78±0.06	0.75±0.07	0.79±0.09	0.76±0.12	0.73±0.09	0.83±0.09	0.81±0.07
**Airflow obstruction**							
FEV_1_/FVC <0.7^+^	8.1	20.1	15.4	26.3	38.4	8.8	5.0
FEV_1_/FVC <LLN^+,§^	5.0	9.6	7.6	14.9	27.5	4.3	11.3
**Doctor-diagnosed asthma**	7.2	8.6	4.5	7.1	9.9	14.2	0
**Respiratory medication**	3.3	4.9	6.7	11.8	8.0	7.7	0

Data are presented as % or mean±sd, unless otherwise stated; percentages may not total 100% due to rounding. FEV_1_: forced expiratory volume in 1 s; FVC: forced vital capacity; LLN: lower limit of normal; NA: not assessed. ^#^: self-reported lifetime nonsmoking. ^¶^: the categorical variable “education” is defined differently in different cohorts. ^+^: values derived from pre-bronchodilation spirometry. ^§^: LLN values estimated using Global Lung Initiative 2012 reference equations [11]. ^ƒ^: LifeLines: nonrandom selection of samples for DNA methylation typing (current smokers *versus* never-smokers). ^##^: NSPHS: information obtained on current smoking status (yes/no).

Across all discovery EWAS meta-analyses, we identified 111 CpG markers for replication (p<5×10^−7^: 74 for FEV_1_, 16 for FVC and 47 for FEV_1_/FVC) (supplementary tables S1 and S2). We present here the results for FEV_1_/FVC (for FEV_1_ and FVC, refer to the supplementary material).

### Cross-sectional associations without smoking adjustment

**FIGURE 2:**
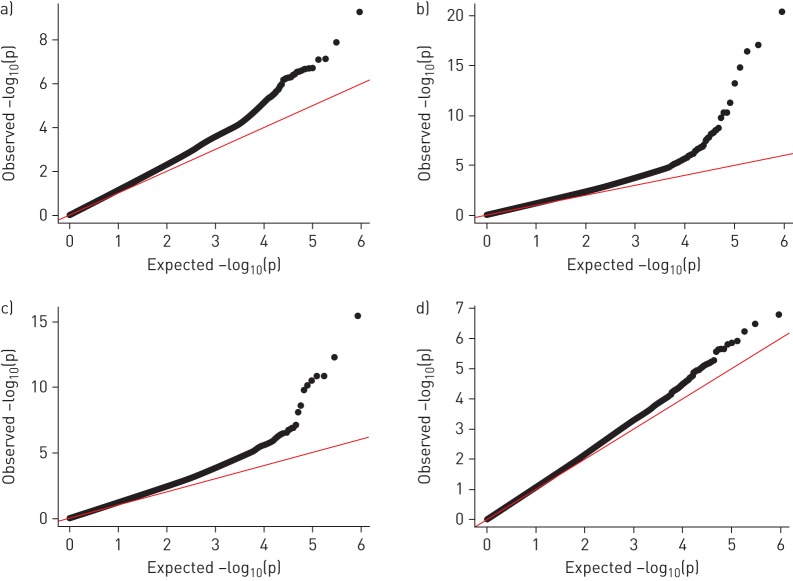
a, b) Effect of ageing on the associations between DNA methylation (DNAme) and lung function: quantile–quantile plots of the cross-sectional covariate-adjusted discovery epigenome-wide association study (EWAS) (M_base_^#^) on forced expiratory volume in 1 s (FEV_1_)/forced vital capacity (FVC) at a) time-point 1 and b) time-point 2, all participants. Increase in numbers of signals with ageing. For FEV_1_/FVC, we identified 21 CpGs at time-point 2 compared with three CpGs at time-point 1 to be statistically significant. Meta-analyses were performed without genomic control (for time-point 1 inflation factor λ=1.15 and for time-point 2 inflation factor λ=1.14). For analogous figure for cross-sectional associations with FEV_1_ and FVC, see supplementary figure S2. c, d) Effect of smoking adjustment on the associations between DNAme and lung function: quantile–quantile plots of c) the repeat cross-sectional covariate-adjusted discovery EWAS (M_base_^#^; inflation factor λ=1.13) and d) additionally smoking adjusted (M_smok_^¶^; inflation factor λ=1.05), all participants. Decrease in numbers of signals after smoking adjustment. ^#^: base model (M_base_) EWAS was covariate adjusted for age, age squared, height, squared deviation from the mean of height, sex and interaction terms of age, age squared, height and squared deviation of height with sex, education (low, medium and high), body mass index, spirometer type, study centre, and cell composition. ^¶^: smoking-adjusted model (M_smok_): covariates applied for M_base_ and additionally smoking status and pack-years smoked.

In the study-specific and meta-analysed discovery EWAS, the number of lung function-associated DNAme increased from the first to second cross-sectional time-point in the same participants, despite age adjustment (figure 2a and b). We therefore meta-analysed cross-sectional discovery and replication results from the older participants' age time-point available. We observed 29 cross-sectional CpG associations with FEV_1_/FVC. 27 of them replicated formally (Bonferroni correction, p<0.0011; 47 tests on FEV_1_/FVC) (table 3 and supplementary table S3). All replicated CpG lung function associations were exclusively DNAme previously associated with smoking [2]. Successful replication was observed for cg05575921 (*AHRR*), showing the strongest signal for FEV_1_ and FEV_1_/FVC (FEV_1_/FVC: p-value combining discovery and replication cohorts (p_combined_)=7.22×10^−50^) among all identified lung function DNAme markers. Methylation at this CpG, previously shown to be hypomethylated with increased smoking, showed positive cross-sectional lung function association. The top 10 CpGs associated with FEV_1_/FVC (table 3) were located in six loci: cg03636183 (*F2RL3*), cg21566642, cg01940273 and cg03329539 (vicinity of *ALPPL2*), cg05575921 and cg21161138 (*AHRR*), cg23771366 and cg11660018 (*PRSS23*), cg21611682 (*LRP5*), and cg15342087 (*IER3*). The same CpGs, along with cg19572487 (*RARA*), were also among the top 11 markers cross-sectionally associated with FEV_1_. Formal replication of cross-sectional associations with FEV_1_ was observed for 44 CpGs and with FVC for three CpGs (supplementary tables S4 and S5). Similar results were found for repeat cross-sectional analyses (EWAS_repeat_) (supplementary table S6 and supplementary figure S3).

**TABLE 3:** Combined epigenome-wide association study (EWAS) meta-analyses of cross-sectional associations^#^ of CpG markers with forced expiratory volume in 1 s (FEV_1_)/forced vital capacity (FVC) in all participants: base model covariate-adjusted EWAS (M_base_^¶^)

**CpG**	**Chr.**	**Position (hg19)**	**Locus**	β**^#^±se**	**p-value meta-analysis**	**Direction of effects^+^**	**p-value between-study heterogeneity**	**Replicated p<0.0011^§^**	**Previously reported smoking CpG^ƒ^**	**Previously reported smoking p**_**FDR**_**-value^ƒ^**	**Previously reported smoking association direction of effect^ƒ^**
**cg05575921**	5	373 378	*AHRR*	0.124±0.008	7.22×10^−50^	+/+/+/+/+/+/+	0.023	Yes	Yes^##^	6.10×10^−22^	(−)
**cg03636183**	19	17 000 585	*F2RL3*	0.201±0.015	4.50×10^−43^	+/+/+/+/+/+/+	0.008	Yes	Yes^##^	5.70×10^−17^	(−)
**cg21566642**	2	233 284 661	*ALPPL2*	0.151±0.011	5.02×10^−43^	+/+/+/+/+/+/+	0.043	Yes	Yes^##^	4.50×10^−21^	(−)
**cg01940273**	2	233 284 934	*ALPPL2*	0.206±0.015	4.09×10^−41^	+/+/+/+/+/+/+	0.031	Yes	Yes^##^	9.80×10^−30^	(−)
**cg03329539**	2	233 283 329	*ALPPL2*	0.257±0.023	5.58×10^−30^	+/+/+/+/+/+/+	0.628	Yes	Yes	9.70×10^−16^	(−)
**cg21161138**	5	399 360	*AHRR*	0.243±0.021	9.72×10^−30^	+/+/+/+/+/+/+	0.152	Yes	Yes^##^	7.90×10^−13^	(−)
**cg23771366**	11	86 510 998	*PRSS23*	0.233±0.022	5.38×10^−27^	+/+/+/+/+/+/+	0.286	Yes	Yes	1.90×10^−14^	(−)
**cg11660018**	11	86 510 915	*PRSS23*	0.238±0.023	3.40×10^−26^	+/+/+/+/+/+/+	0.318	Yes	Yes	4.40×10^−21^	(−)
**cg21611682**	11	68 138 269	*LRP5*	0.309±0.030	1.26×10^−25^	+/+/+/+/+/+/+	0.049	Yes	Yes	4.20×10^−15^	(−)
**cg15342087**	6	30 720 209	*IER3*	0.359±0.036	5.44×10^−24^	+/+/+/+/+/+/+	0.169	Yes	Yes	3.90×10^−14^	(−)
**cg26703534**	5	377 358	*AHRR*	0.266±0.026	7.34×10^−24^	+/+/+/+/+/+/+	0.101	Yes	Yes	7.20×10^−18^	(−)
**cg25648203**	5	395 444	*AHRR*	0.250±0.026	9.84×10^−22^	+/+/+/+/+/+/+	0.194	Yes	Yes	2.70×10^−11^	(−)
**cg19572487**	17	38 476 024	*RARA*	0.196±0.021	8.87×10^−21^	+/+/+/+/+/+/+	0.018	Yes	Yes	1.60×10^−16^	(−)
**cg00310412**	15	74 724 918	*SEMA7A*	0.261±0.028	4.01×10^−20^	+/+/+/+/+/+/+	0.275	Yes	Yes	1.20×10^−13^	(−)
**cg24859433**	6	30 720 203	*IER3*	0.303±0.034	2.05×10^−19^	+/+/+/+/+/+/+	0.067	Yes	Yes^##^	2.20×10^−9^	(−)
**cg09935388**	1	92 947 588	*GFI1*	0.105±0.012	7.05×10^−19^	+/+/+/+/+/+/+	0.034	Yes	Yes^##^	7.00×10^−14^	(−)
**cg14753356**	6	30 720 108	*IER3*	0.189±0.021	9.08×10^−19^	+/+/+/+/+/+/+	0.405	Yes	Yes	2.30×10^−14^	(−)
**cg04885881**	1	11 123 118	*SRM/EXOSC10*	0.168±0.020	5.66×10^−18^	+/+/+/+/+/+/+	0.670	Yes	Yes	2.70×10^−11^	(−)
**cg25949550**	7	145 814 306	*CNTNAP2*	0.335±0.039	6.04×10^−18^	+/+/+/+/+/+/+	0.013	Yes	Yes	9.30×10^−21^	(−)
**cg19859270**	3	98 251 294	*GPR15*	0.467±0.055	2.80×10^−17^	+/+/+/+/+/+/+	0.029	Yes	Yes	6.30×10^−17^	(−)
**cg03450842**	10	80 834 947	*ZMIZ1*	0.265±0.031	2.92×10^−17^	+/+/+/+/+/+/+	0.003	Yes	Yes	2.40×10^−11^	(−)
**cg03707168**	19	49 379 127	*PPP1R15A*	0.206±0.025	1.27×10^−16^	+/+/+/+/+/+/+	0.668	Yes	Yes	3.50×10^−7^	(−)
**cg17087741**	2	233 283 010	*ALPPL2*	0.161±0.020	4.48×10^−16^	+/+/+/+/+/+/−	<0.001	Yes	Yes	6.10×10^−7^	(−)
**cg21140898**	1	51 442 318	*CDKN2C*	0.120±0.017	4.46×10^−13^	+/+/+/+/+/+/+	0.103	Yes	Yes	3.70×10^−8^	(−)
**cg01899089**	5	369 969	*AHRR*	0.172±0.027	1.47×10^−10^	+/+/+/+/+/+/+	0.005	Yes	Yes	1.80×10^−12^	(−)
**cg08763102**	4	3 079 751	*HTT*	0.225±0.039	1.20×10^−8^	+/+/+/+/+/+/−	0.001	Yes	Yes	3.80×10^−15^	(−)
**cg21282907**	6	74 289 980	*SLC17A5*	0.176±0.031	1.28×10^−8^	+/+/+/+/+/+/−	0.003	No	Yes	1.28×10^−2^	(−)
**cg20853880**	2	10 184 444	*KLF11*	0.077±0.014	6.05×10^−8^	+/+/+/+/+/+/+	0.052	No	Yes	3.70×10^−7^	(−)
**cg16391678**	16	30 485 597	*ITGAL*	0.164±0.031	1.15×10^−7^	+/+/+/+/+/+/−	0.003	Yes	Yes	3.00×10^−11^	(−)

Chr.: chromosome; hg19: human genome build 19; β: coefficient of association; FDR: false discovery rate. Meta-analyses of cross-sectional associations obtained using data from the oldest time-point available: time-point 2 of ECRHS, NFBC1966, SAPALDIA and LBC1936; time-point 1 of KORA, LifeLines and NSPHS. For complete results for FEV_1_/FVC associations, see supplementary table S3. See supplementary tables S4 and S5 for analogous results for FEV_1_ and FVC, respectively. ^#^: presentation of CpG markers showing meta-analysis p<5×10^−7^ in the combined meta-analysis. Note that DNA methylation predictors used were technical bias-adjusted, normalised residuals and thus effect sizes of the association (β) are not directly comparable to effect sizes reported elsewhere using normalised % methylation as predictor. ^¶^: base model (M_base_) epigenome-wide association study was covariate adjusted for age, age squared, height, squared deviation from the mean of height, sex and interaction terms of age, age squared, height and squared deviation of height with sex, education (low, medium and high), body mass index, spirometer type, study centre as well as cell composition. ^+^: order of cohorts: ECRHS, NFBC1966, SAPALDIA, KORA, LBC1936, LifeLines and NSPHS (FTC was excluded from this meta-analysis, given the smaller sample size and lower mean age (30.4 years) compared with the other adult cohorts (ECRHS (54.5 years), NFBC1966 (46.3 years), SAPALDIA (58.8 years) and LBC1936 (76.3 years), and the single available time-point for KORA (60.1 years), LifeLines (46.7 years) and NSPHS (55.1 years)). ^§^: replication was defined for association if replication p<0.0011 (multiple testing correction, 47 tests for FEV_1_/FVC). ^ƒ^: smoking CpGs defined on the reported FDR-corrected p<0.05 for association reported with smoking status and reported direction of effects for association with smoking [2]. ^##^: smoking CpG previously reported to mediate the effect of smoking on lung function [4].

### Cross-sectional smoking-adjusted associations

The smoking-adjusted EWAS (M_smok_) resulted in fewer genome-wide significant results (figure 2c and d). Yet, despite adjustment for self-report of smoking history, the top five CpGs were known smoking-related CpGs. DNAme at cg05575921 (*AHRR)* remained the top cross-sectional association signal for FEV_1_/FVC (p_combined_=2.21×10^−11^) (supplementary table S7).

### Predictive associations without smoking adjustment

**FIGURE 3:**
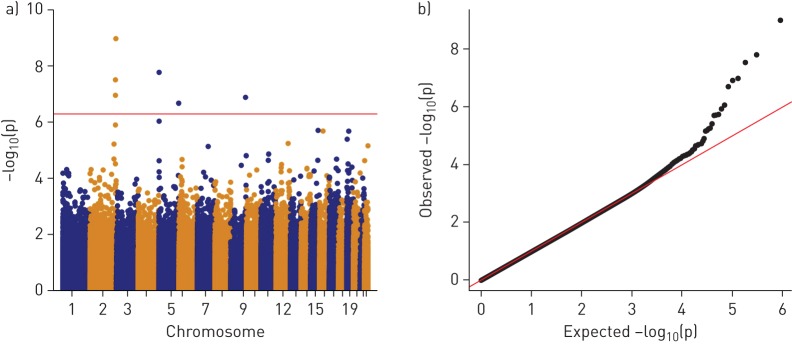
a) Manhattan and b) quantile–quantile plots of the covariate-adjusted prediction^#^ epigenome-wide association study (EWAS) (M_base_^¶^) on forced expiratory volume in 1 s (FEV_1_)/forced vital capacity (FVC), all participants. Meta-analysis of the prediction association was performed without genomic control (inflation factor λ=0.95). For analogous figure for associations with change in FEV_1_ and FVC, see supplementary figure S4. ^#^: predictive associations of DNA methylation at first time-point with change in lung function during follow-up. ^¶^: base model (M_base_) EWAS was covariate adjusted for age, age squared, height, FEV_1_/FVC at time-point 1, squared deviation from the mean of height, sex and interaction terms of age, age squared, height and squared deviation of height with sex, education (low, medium and high), body mass index, spirometer type, study centre, and cell composition.

The prediction EWAS results (table 4 and figure 3) revealed that DNAme at time-point 1 (DNAme1) at six of nine sentinel CpGs (p<5×10^−7^) associated with change in FEV_1_/FVC was replicated (cg05575921 and cg21161138 (*AHRR*), cg21566642, cg01940273 and cg03329539 (vicinity of *ALPPL2*), and cg03636183 (*F2RL3*)). These six replicated CpGs were smoking-related markers. They were also associated with cross-sectional FEV_1_/FVC and four of them also with predicting change in FEV_1_ (*AHRR* (cg05575921), *ALPPL2* (cg05951221 and cg01940273) and *F2RL3* (cg03636183)) (supplementary table S8).

**TABLE 4:** Combined meta-analyses of the prediction associations^#^ of CpG markers on annual change in forced expiratory volume in 1 s (FEV_1_)/forced vital capacity (FVC) in all participants: base model adjustment (M_base_^¶^)

**CpG**	**Chr.**	**Position (hg19)**	**Locus**	**Combined meta-analysis** **(ECRHS/NFBC1966/SAPALDIA/KORA/LBC1936)**					**Previously reported smoking CpG^ƒ^**	**Previously reported smoking p**_**FDR**_**-value^ƒ^**	**Previously reported smoking association direction of effect^ƒ^**
				β**^#^±se**	**p-value meta-analysis**	**Direction of effects^+^**	**p-value between-study heterogeneity**	**Replicated p<0.0011^§^**			
**cg05575921**	5	373 378	*AHRR*	0.006±0.001	2.77×10^−13^	+/+/+/+/+	0.005	Yes	Yes^##^	6.10×10^−22^	(−)
**cg21566642**	2	233 284 661	*ALPPL2*	0.006±0.001	3.17×10^−11^	+/+/+/+/+	0.235	Yes	Yes^##^	4.50×10^−21^	(−)
**cg01940273**	2	233 284 934	*ALPPL2*	0.009±0.001	4.93×10^−11^	+/+/+/+/+	0.023	Yes	Yes^##^	9.80×10^−30^	(−)
**cg21161138**	5	399 360	*AHRR*	0.011±0.002	5.81×10^−9^	+/+/+/+/+	0.103	Yes	Yes^##^	7.90×10^−13^	(−)
**cg03636183**	19	17 000 585	*F2RL3*	0.008±0.001	6.22×10^−9^	+/+/+/+/+	0.001	Yes	Yes^##^	5.70×10^−17^	(−)
**cg01377124**	2	237 172 609	*ASB18*	−0.018±0.003	7.38×10^−8^	−/−/+/−/+	0.005	No	No	NA	NA
**cg03329539**	2	233 283 329	*ALPPL2*	0.011±0.002	7.66×10^−8^	+/+/+/+/+	0.015	Yes	Yes	9.70×10^−16^	(−)
**cg07222133**	5	179 499 488	*RNF130*	−0.009±0.002	2.45×10^−7^	?/−/+/−/+	<0.001	No	No	NA	NA
**cg14366110**	9	133 779 382	*FIBCD1*	0.014±0.003	9.62×10^−7^	+/+/+/−/−	0.206	No	No	NA	NA

Chr.: chromosome; hg19: human genome build 19; β: coefficient of association; FDR: false discovery rate; NA: not assessed. For complete results for FEV_1_/FVC and analogous results for FEV_1_ and FVC, see supplementary table S8. ^#^: predictive associations of DNA methylation at first time-point (DNAme1) with annual change in lung function during follow-up, defined as (lung function at second time-point−lung function at first time-point)/time of follow-up (years). Presentation of CpG markers showing meta-analysis p-value<5×10^−7^ at discovery or combined meta-analyses level. CpGs shown sorted by statistical significance of combined meta-analysis results. Note that DNAme predictors used were technical bias-adjusted, normalised residuals and thus effect sizes of the association (β) are not directly comparable to effect sizes reported elsewhere using normalised % methylation as predictor. ^¶^: base model (M_base_) epigenome-wide association study was covariate adjusted for age, age squared, height, FEV_1_/FVC at time-point 1, squared deviation from the mean of height, sex and interaction terms of age, age squared, height and squared deviation of height with sex, education (low, medium and high), body mass index, spirometer type, study centre as well as cell composition. ^+^: order of cohorts: ECRHS, NFBC1966, SAPALDIA, KORA and LBC1936. ^§^: replication was defined for association if replication p<0.0011 (multiple testing correction, 47 tests for FEV_1_/FVC). ^ƒ^: smoking CpGs defined on the reported FDR-corrected p<0.05 for association reported with smoking status and reported direction of effects for association with smoking [2]. ^##^: smoking CpG previously reported to mediate the effect of smoking on lung function [4].

### Associations in never-smokers

The agnostic discovery EWAS (M_base_) in never-smokers, similar to the entire sample, showed more statistically significant associations at time-point 2 (older age). Eight CpGs were cross-sectionally associated with FEV_1_/FVC in never-smokers (p<5×10^−7^), but none replicated (table 5 and supplementary figure S5). The CpG cg14366110 (*FIBCD1*) showed predictive association of DNAme1 with change in FEV_1_/FVC (p_discovery_=4.2×10^−9^, p_combined_=3.6×10^−9^) in never-smokers, but it did not replicate in KORA and LBC1936 (p_replication_=0.439; replication cohorts with lung function at two time-points). The direction of effect, however, was consistent (table 6; see supplementary table S9 for cross-sectional associations and supplementary table S10 for prediction associations) in discovery and replication cohorts.

**TABLE 5:** Combined meta-analyses^#^ of cross-sectional associations on forced expiratory volume in 1 s (FEV_1_)/forced vital capacity (FVC) in never-smokers only: base model adjustment (M_base_^¶^)

**CpG**	**Chr.**	**Position (hg19)**	**Locus**	**Combined meta-analysis** **(ECRHS/NFBC1966/SAPALDIA/KORA/LBC1936/Lifelines/NSPHS)**					**Previously reported smoking CpG^ƒ^**	**Previously reported smoking p**_**FDR**_**-value^ƒ^**	**Previously reported smoking association direction of effect^ƒ^**
				β**^#^±se**	**p-value meta-analysis**	**Direction of effects^+^**	**p-value between-study heterogeneity**	**Replicated p<0.0011^§^**			
**cg09884077**	15	23 086 698	*NIPA1*	−0.308±0.084	0.0003	−/−/−/+/−/−/−	0.001	No	No	NA	NA
**cg25758394**	1	3 623 859	*TP73*	0.213±0.083	0.0107	?/?/+/−/−/+/−	<0.001	No	No	NA	NA
**cg18664508**	3	169 487 465	*ARPM1*	−0.308±0.072	2.02×10^−5^	+/−/−/−/−/−/−	<0.001	No	No	NA	NA
**cg19268386**	15	23 086 595	*NIPA1*	−0.263±0.140	0.0615	?/?/−/−/−/−/−	<0.001	No	No	NA	NA
**cg15981995**	3	169 487 311	*ARPM1*	−0.231±0.073	0.0016	?/?/−/−/−/−/+	<0.001	No	No	NA	NA
**cg05785298**	1	204 654 622	*LRRN2*	−0.423±0.111	1.41×10^−4^	−/+/−/+/−/+/−	0.001	No	No	NA	NA
**cg20278790**	20	57 583 474	*CTSZ*	0.319±0.070	5.01×10^−6^	−/+/+/+/−/−/−	<0.001	No	No	NA	NA
**cg13562246**	8	33 368 277	*C8orf41*	0.349±0.074	2.67×10^−6^	+/+/+/+/+/−/+	0.206	No	No	NA	NA

Chr.: chromosome; hg19: human genome build 19; β: coefficient of association; FDR: false discovery rate; NA: not assessed. For complete results for FEV_1_/FVC and for FEV_1_ and FVC in never-smokers, see supplementary table S8. ^#^: presentation of CpG markers showing meta-analysis p<5×10^−7^ at discovery level for cross-sectional association at time-point 2, using data from time-point 2 of ECRHS, NFBC1966, SAPALDIA and LBC1936 and from time-point 1 of KORA, LifeLines and NSPHS. Note that DNA methylation predictors used were technical bias-adjusted, normalised residuals and thus effect sizes of the association (β) are not directly comparable to effect sizes reported elsewhere using normalised % methylation as predictor. ^¶^: base model (M_base_) epigenome-wide association study was covariate adjusted for age, age squared, height, squared deviation from the mean of height, sex and interaction terms of age, age squared, height and squared deviation of height with sex, education (low, medium and high), body mass index, spirometer type, study centre as well as cell composition. ^+^: order of cohorts: ECRHS, NFBC1966, SAPALDIA, KORA, LBC1936, LifeLines and NSPHS.  ^§^: replication was defined for association if replication p<0.0011 (multiple testing correction, 47 tests for FEV_1_/FVC). ^ƒ^: smoking CpGs defined on the reported FDR-corrected p<0.05 for association reported with smoking status and reported direction of effects for association with smoking [2].

**TABLE 6:** Combined meta-analyses of the prediction associations^#^ of CpG markers on annual change in forced expiratory volume in 1 s (FEV_1_)/forced vital capacity (FVC) in never-smokers only: base model adjustment (M_base_^¶^)

**CpG**	**Chr.**	**Position (hg19)**	**Locus**	**Combined meta-analysis** **(ECRHS/NFBC1966/SAPALDIA/KORA/LBC1936)**					**Previously reported smoking CpG^ƒ^**	**Previously reported smoking p**_**FDR**_**-value^ƒ^**	**Previously reported smoking association direction of effect^ƒ^**
				β**^#^±se**	**p-value meta-analysis**	**Direction of effects^+^**	**p-value between-study heterogeneity**	**Replicated p<0.0011^§^**			
**cg14366110**	9	133 779 382	*FIBCD1*	0.017±0.003	3.60×10^−9^	+/+/−/+/+	0.315	No	No	NA	NA
**cg11216682**	2	131 113 867	*PTPN18*	−0.017±0.003	1.10×10^−7^	+/−/+/−/−	0.282	No	No	NA	NA

Chr.: chromosome; hg19: human genome build 19; β: coefficient of association; FDR: false discovery rate; NA: not assessed. For complete results for FEV_1_/FVC and for analogous results for FEV1 and FVC, see supplementary table S9. ^#^: predictive associations of DNA methylation at first time-point (DNAme1) with annual change in lung function during follow-up, defined as (lung function at second time-point−lung function at first time-point)/time of follow-up (years). Presentation of CpG markers showing meta-analysis p<5×10^−7^ at discovery or replication level. Note that DNAme predictors used were technical bias-adjusted, normalised residuals and thus effect sizes of the association (β) are not directly comparable to effect sizes reported elsewhere using normalised % methylation as predictor. ^¶^: base model (M_base_) epigenome-wide association study was covariate adjusted for age, age squared, height, FEV1/FVC at time-point 1, squared deviation from the mean of height, sex and interaction terms of age, age squared, height and squared deviation of height with sex, education (low, medium and high), body mass index, spirometer type, study centre as well as cell composition. ^+^: order of cohorts: ECRHS, NFBC1966, SAPALDIA, KORA and LBC1936. ^§^: replication was defined for association if replication p<0.0011 (multiple testing correction, 47 tests for FEV_1_/FVC). ^ƒ^: smoking CpGs defined on the reported FDR-corrected p<0.05 for association reported with smoking status and reported direction of effects for association with smoking [2].

### Characterisation of replicated CpGs

None of the not-smoking-related discovery-identified sentinel CpGs (n=25) were confirmed by replication. In contrast, 78% of the sentinel CpGs (n=86) had previously been identified as smoking related, and 57 of these (mapping to 43 loci) formally replicated across all models and lung function outcomes tested (supplementary table S11). They were used jointly for functional annotation and pathway analyses (supplementary tables S12–S16). Briefly, these 57 lung function-associated CpGs displayed enrichment for transcription factors, such as *RELA* (false discovery rate-adjusted p-value (p_FDR_)=0.002) and *EP300* (p_FDR_=0.004), and suggestive enrichment (p_FDR_<0.1) for the chromatin state model of flanking active transcription start sites, of transcription at gene 5′ and 3′, and of enhancers. No significant pathways were revealed using Ingenuity Pathway Analysis database or Gene Ontology term enrichment. Transcriptional misregulation in cancer, pathways in cancer and regulation of actin cytoskeleton were identified (p_FDR_<0.05) using KEGG (Kyoto Encyclopedia of Genes and Genomes) pathways enrichment.

Using the weighted Kolmogorov–Smirnov test on the entire EWAS discovery results, we noted statistically significant enrichment for smoking-related CpGs among the lung function-associated CpGs. This enrichment was also present in the smoking-adjusted EWAS and even in the EWAS restricted to never-smokers (supplementary table S17).

### Association of adult lung function CpG markers with childhood lung function

Using the same scheme of analysis as for the adult replication cohorts, none of the sentinel CpGs showed associations with FEV_1_, FVC and FEV_1_/FVC in the childhood replication cohorts (ALSPAC and IOWBC) (supplementary table S18). The strongest associations observed in children (p<0.01) were for five CpGs not known to be smoking-related DNAme markers and one smoking-related CpG (cg00310412 (*SEMA7A*)).

### Comparison with published DNAme–lung function association reports

Our agnostic results were compared with previously reported lung function-specific [4, 6, 7, 12] or chronic obstructive pulmonary disease (COPD)-specific [13, 14] DNAme. We retrieved all CpGs reported being associated with lung function (n=376) for a look-up in the cross-sectional FEV_1_, FVC and FEV_1_/FVC associations at time-point 2. Only 12 out of 376 CpGs showed evidence for association (Bonferroni correction for 376 tests: p<1.3×10^−4^) (supplementary table S19). Notably, the most recently reported CpG markers [4, 6], having also been related to smoking, showed consistent associations with lung function, *e.g*. cg05575921 and cg21161138 (*AHRR*), cg05951221 (near *ALPPL2*), and cg06126421 (*IER3*). They were among our top replicated lung function association signals.

### Two-sample Mendelian randomisation investigation

To assess the causality of replicated DNAme–lung function association, we conducted a *post hoc* Mendelian randomisation look-up using publicly available databases [15, 16]. Genetic instruments were identified for 12 replicated CpGs. A two-sample Mendelian randomisation on cross-sectional lung function could be completed for seven CpGs (supplementary table S20). Results support causal effects for cg23771366 and cg11660018 (*PRSS23*), cg21990700 (*C1RL*), and cg00073460 (*ZC3H12D*) on FEV_1_, and for cg00073460 (*ZC3H12D*) and cg24086068 (*SHROOM3*) on FVC.

### Integration of DNAme into a smoking index

A recent smoking EWAS followed-up by a mediation analysis identified 10 CpGs as mediators of the smoking–lung function association [4]. Eight of these mediating CpGs were among our replicated lung function-associated CpGs (supplementary table S21). In a *post hoc* mediation analysis in SAPALDIA, we showed statistically significant average causal mediation on lung function for nine of these mediating CpGs (FEV_1_/FVC: table 7; FEV_1_ and FVC: supplementary table S22).

**TABLE 7:** Mediation^#^ analysis on the role of previously reported CpGs in the smoking association with forced expiratory volume in 1 s (FEV_1_)/forced vital capacity (FVC): the SAPALDIA cohort

**CpG^¶^**	**Locus**	**ACME**		**ADE**		**Total effect**		**Proportion**	
		**Estimate (95% CI)**	**p-value**	**Estimate (95% CI)**	**p-value**	**Estimate (95% CI)**	**p-value**	**Estimate (95% CI)**	**p-value**
**cg01940273**	*ALPPL2*	−0.0079 (−0.0119– −0.0041)	<0.0001	−0.0026 (−0.0129–0.0077)	0.604	−0.0106 (−0.0203– −0.0014)	0.026	0.7313 (0.2616–3.4325)	0.026
**cg03636183**	*F2RL3*	−0.0080 (−0.0122– −0.0040)	<0.0001	−0.0029 (−0.0126–0.0062)	0.556	−0.0108 (−0.0197– −0.0021)	0.018	0.7312 (0.2819–2.9097)	0.018
**cg05575921**	*AHRR*	−0.0102 (−0.0147– −0.0055)	<0.0001	−0.0008 (−0.0109–0.0086)	0.870	−0.0110 (−0.0202– −0.0020)	0.012	0.9213 (0.3818–4.0453)	0.012
**cg05951221**	*ALPPL2*	−0.0075 (−0.0122– −0.0030)	0.002	−0.0033 (−0.0131–0.0062)	0.520	−0.0109 (−0.0197– −0.0022)	0.020	0.6836 (0.1942–2.7656)	0.022
**cg06126421**	*IER3*	−0.0054 (−0.0093– −0.0017)	<0.0001	−0.0049 (−0.0148–0.0049)	0.328	−0.0103 (−0.0194– −0.0012)	0.030	0.5233 (0.1050–2.5558)	0.030
**cg09935388**	*GFI1*	−0.0033 (−0.0058– −0.0010)	0.002	−0.0073 (−0.0168–0.0022)	0.122	−0.0105 (−0.0198– −0.0013)	0.034	0.3009 (0.0568–1.4190)	0.036
**cg21161138**	*AHRR*	−0.0056 (−0.0089– −0.0025)	<0.0001	−0.0052 (−0.0146–0.0043)	0.282	−0.0108 (−0.0194– −0.0020)	0.020	0.5127 (0.1647–2.0961)	0.020
**cg21566642**	*ALPPL2*	−0.0098 (−0.0145– −0.0057)	<0.0001	−0.0014 (−0.0116–0.0089)	0.796	−0.0112 (−0.0209– −0.0011)	0.024	0.8663 (0.3453–4.6567)	0.024
**cg22994830**	*PRKAR1B*	−0.0002 (−0.0009–0.0003)	0.542	−0.0103 (−0.0201– −0.0010)	0.028	−0.0105 (−0.0202– −0.0013)	0.024	0.0103 (−0.0470–0.1595)	0.550
**cg24859433**	*IER3*	−0.0024 (−0.0053–0.0002)	0.068	−0.0082 (−0.0179–0.0013)	0.112	−0.0107 (−0.0201– −0.0014)	0.022	0.2186 (−0.0438–1.2776)	0.090

ACME: average causal mediation effect; ADE: average direct effect. For analogous results for FEV_1_ and FVC, see supplementary table S22. ^#^: performed using the R package mediation [17]. ^¶^: previously reported candidate CpG for mediation of effect of smoking on lung function [4].

**FIGURE 4:**
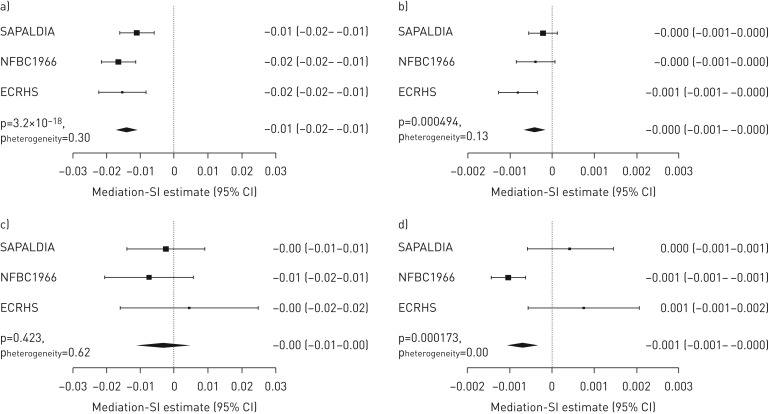
Forest plots of cohort-specific results and meta-analyses of the association of the mediation smoking index (Mediation-SI) with forced expiratory volume in 1 s (FEV_1_)/forced vital capacity (FVC) and change in FEV_1_/FVC in a, b) ever-smokers and c, d) never-smokers in the discovery cohorts: a, c) time-point 2 and b, d) prediction. Associations run applying base model adjustment (M_base_^#^). ^#^: base model (M_base_) epigenome-wide association study was covariate adjusted for age, age squared, height, squared deviation from the mean of height, sex and interaction terms of age, age squared, height and squared deviation of height with sex, education (low, medium and high), body mass index, spirometer type, study centre, and cell composition. Prediction models were additionally adjusted for FEV_1_/FVC at time-point 1.

**FIGURE 5:**
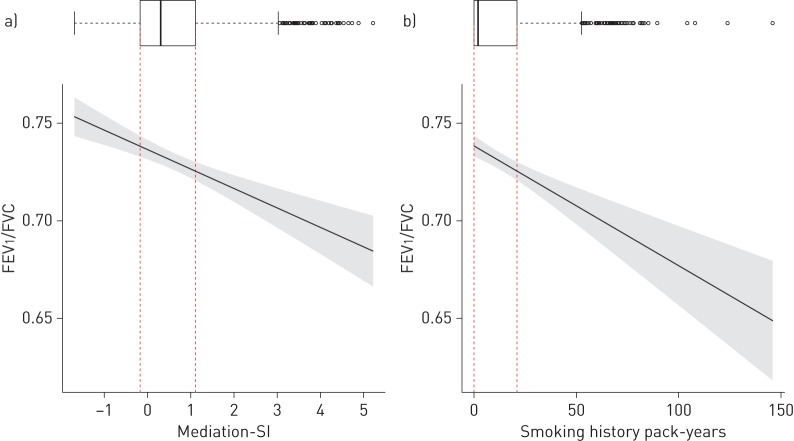
Distribution and association^#^ of a) mediation smoking index (Mediation-SI)^¶^ and b) self-reported smoking history (pack-years) with forced expiratory volume in 1 s (FEV_1_)/forced vital capacity (FVC) with 95% confidence intervals (shaded). Box plots of a) Mediation-SI (median (range) 0.3 (−1.7–5.2)) and b) pack-years (median (range) 2.0 (0–145.9)) in all participants of SAPALDIA are shown at the top of each panel. Red dotted lines indicate box plot interquartile range (IQR) borders. Whiskers indicate 1.5 IQR of the lower and upper quartile; outliers are indicated. For analogous figures for associations of Mediation-SI with FEV_1_ and FVC, see supplementary figures S6 and S7, respectively. ^#^: associations were adjusted for the base model (M_base_): age, age squared, height, squared deviation from the mean of height, sex and interaction terms of age, age squared, height and squared deviation of height with sex, education (low, medium and high), body mass index, spirometer type, study centre, and cell composition. ^¶^: Mediation-SI can be constructed for all participants irrespective of their smoking status. The M_base_-adjusted model explained 17.5% of the variance in the outcome. The M_base_-adjusted model additionally adjusted for the Mediation-SI explained 19.6% of the FEV_1_/FVC variance (total adjusted R^2^=0.196) of which 2.8% of the variance was specifically explained by the Mediation-SI variable. This was comparable to the variance explained by the M_base_-adjusted model additionally adjusted for pack-years and smoking status corresponding to the M_smok_ model (R^2^=0.198, and with 1.6% of the variance specifically explained by the pack-years variable). Model including both smoking adjustments (M_smok_ and additionally Mediation-SI) explained 20.1% of the FEV_1_/FVC variance.

**FIGURE 6:**
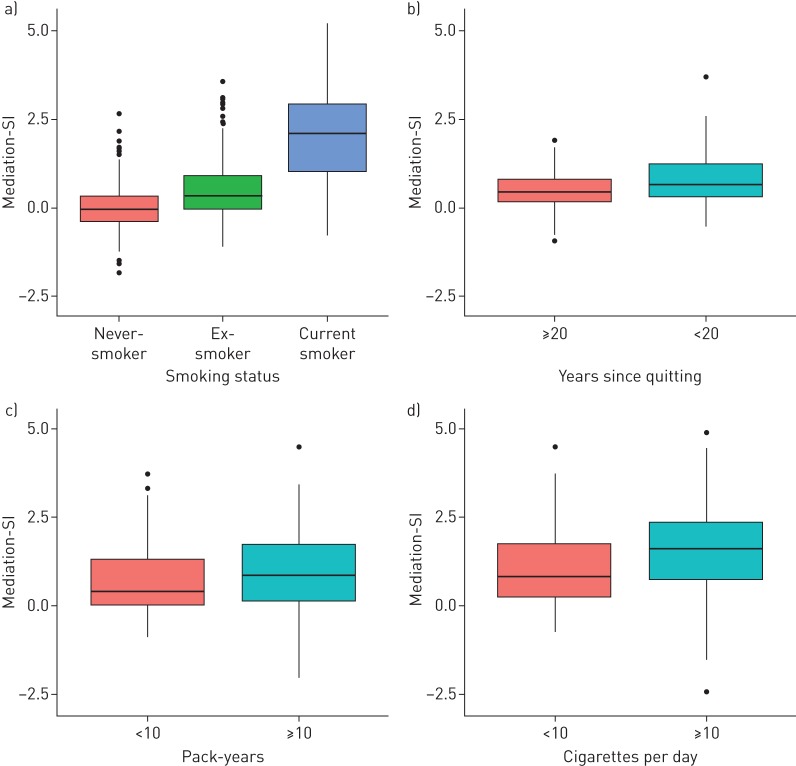
Distribution of adjusted mediation smoking index (Mediation-SI) in SAPALDIA at time-point 2. a) Smoking status: adjusted for age, sex and education. Never-smokers (n=395), ex-smokers (n=356) and current smokers (n=211). b) Years since quitting: adjusted for age, sex, education, pack-years and cigarettes per day. Ex-smokers (n=356). c) Pack-years: adjusted for age, sex, education and cigarettes per day. Current smokers (n=211). d) Cigarettes per day: adjusted for age, sex, education and pack-years. Current smokers (n=211). Data are presented as median with interquartile range (IQR) (boxes) and 1.5 IQR of the lower and upper quartile (whiskers); outliers are indicated.

To assess the combined effect of these smoking exposure-mediating CpGs on lung function, we constructed a mediation smoking index (Mediation-SI). Its association with lung function by smoking status was tested in covariate-adjusted regression models in the discovery cohorts and following EWAS models (SAPALDIA, ECRHS and NFBC1966). Meta-analysed results of Mediation-SI showed strong association with cross-sectional FEV_1_/FVC in all participants and ever-smokers (table 8 and figure 4; FEV_1_ and FVC: supplementary table S23). Mediation-SI association in all participants was more pronounced for cross-sectional (β±se −1.2±0.13; p=2.65×10^−20^) than for prediction association (β±se −0.03±0.01; p=0.0072). We noted comparable associations of Mediation-SI and of pack-years with lung function (figure 5). Both were inversely associated with level of FEV_1_/FVC. Adding Mediation-SI or self-reported smoking history (smoking status and pack-years) to the different M_base_-adjusted statistical models showed a comparable increase in total adjusted R^2^. The highest total adjusted R^2^ was obtained when including both DNAme score and self-reported smoking history. Covariate-adjusted mean Mediation-SI values decreased from never- to ex- to current smokers and from more distant to more recent smoking exposure, with increase in pack-years in current smokers and with fewer years since quitting in ex-smokers (figure 6).

**TABLE 8:** Meta-analyses^#^ of the discovery cohort-specific association of mediation smoking index (Mediation-SI) with forced expiratory volume in 1 s (FEV_1_)/forced vital capacity (FVC) (%), cross-sectionally at time-point 2 and longitudinally predicting the annual change during follow-up, in all study participants, ever and never-smokers: base model adjustment (M_base_^¶^)

	**Cross-sectional meta-analysis at time-point 2^#^**				**Prediction on change in lung function^¶^**			
	β**±se**	**p-value^+^**	**Direction of effects^§^**	**p-value between-study heterogeneity**	β**±se**	**p-value^+^**	**Direction of effects^§^**	**p-value between-study heterogeneity**
**All**	−0.012±0.0013	1.05×10^−20^	−/−/−	0.44	−0.0005±0.0001	8.66×10^−9^	−/−/−	0.006
**Ever-smokers**	−0.014±0.0016	3.28×10^−18^	−/−/−	0.30	−0.0004±0.0001	4.94×10^−4^	−/−/−	0.13
**Never-smokers**	−0.0033±0.0041	0.423	−/−/+	0.62	−0.0007±0.0002	1.73×10^−4^	+/−/+	0.003

β: coefficient of association. For analogous results of associations of Mediation-SI with FEV_1_ and FVC, see supplementary table S23. ^#^: cohort-specific association results for Mediation-SI were meta-analysed. The 10 CpGs contributing Mediation-SI values are shown in supplementary table S21. Note that DNA methylation predictors used were technical bias-adjusted, normalised residuals and thus effect sizes of the association (β) are not directly comparable to effect sizes reported elsewhere using normalised % methylation as predictor. ^¶^: base model (M_base_) covariate adjustment: age, age squared, height, squared deviation from the mean of height, sex and interaction terms of age, age squared, height and squared deviation of height with sex, education (low, medium and high), body mass index, spirometer type, study centre as well as cell composition. Prediction models were additionally adjusted for FEV_1_/FVC at time-point 1. ^+^: p-value of meta-analysis: p<0.008 was considered statistically significant, Bonferroni correction for six tests per lung function outcome. ^§^: order of cohorts: ECRHS, NFBC1966 and SAPALDIA.

The assessment of a second DNAme smoking score (Lung-Function-Genes-SI), based on smoking-related CpGs located in 18 GWAS-identified lung function candidate genes (supplementary table S24), showed less prominent associations with lung function (strongest association observed in ever-smokers for FEV_1_: β±se −0.196±0.053; p=0.0002) (supplementary table S25).

## Discussion

The understanding of how environmental exposure and disease are related to site-specific DNAme status is growing [18, 19]. Our agnostic EWAS on lung function contributes to this body of evidence. Lung function-associated DNAme markers were strongly enriched for smoking-associated loci. More than 50 known smoking CpGs were consistently, and in several cases causally, associated with lung function and its change in adults. The current agnostic approach converges with recent results of DNAme–lung function studies [4, 6, 7] that were *a priori* focusing on smoking-related loci, and included pyrosequencing in blood [7] and lung tissue [4] of some of our strongest association signals, including *AHRR* hypomethylation at cg05575921 and cg21161138, cg05951221 and cg21566642 (*ALPPL2*), and cg06126421 (*IER3)*. A methylation index integrating 10 DNAme that reportedly mediate the effect of smoking on lung function [4] was associated with lung function level and its change in adults.

Smoking is an important risk factor for poor lung function and accelerated decline. Several EWASs identified a large number of differentially methylated CpG markers to be associated with smoking [2–4]. In particular, the hypomethylation of cg05575921, a CpG located in the third intron of the aryl hydrocarbon receptor repressor (*AHRR*) gene, investigated for lung function and respiratory symptoms [4], stands out as a robust indicator of smoking status and smoking history [20]. Given the consistency of the associations observed for cg05575921 and the smoking index containing it in this study, the latter may have potential as a biomarker of clinical utility in predicting smoking-related morbidity and mortality [20, 21]. The positive direction of effects observed in identified DNAme–lung function association is in accordance with the reported hypomethylation of smoking-related DNAme sites. The identified lung function-associated CpGs in this study have been previously reported to be associated with smoking-related molecular phenotypes [22], with increased risk of noncommunicable disease, including cancer [20, 23], and with epigenetically defined accelerated ageing [24].

Whether most smoking-related DNAme markers are only markers of exposure or indirectly associated with lung function [7] or whether some inform on causal disease pathways cannot be answered conclusively by the current study. First, DNAme may just be a more precise measure of smoking exposure than self-reporting, as *AHRR* DNAme was previously shown to correlate with genetic smoking dependency [20]. Second, DNAme identified by previous smoking EWASs [2, 4] may not exclusively have picked up methylation effects of smoking, but methylation related to phenotypes also affected by smoking. In this case, the observed DNAme–lung function associations may result from comorbidity between lung function and other smoking-related phenotypes. However, some of the results are consistent with a causal disease pathway. First, Mendelian randomisation results support causal effects from some DNAme. Unfortunately, no genetic instrument was available for the top ranked *AHRR* signal. Second, our report confirms nine CpGs, including cg05575921 (*AHRR*), previously shown to mediate the effect of smoking of lung function [4]. The observation that many smoking DNAme–lung function associations withstood smoking adjustment is consistent with the mediating role of DNAme between smoking behaviour (more distant predictor) and lung function. Third, smoking was also observed to influence methylation in lung tissue at several lung function CpGs, including at cg05575921 in *AHRR*, and these methylation levels correlated with *AHRR* gene expression [25] and expression of other genes [4]. Hypotheses for a mediating and causal role of smoking-related DNAme include altered *AHRR* DNAme inducing altered phase 2 enzyme activity and toxicant metabolism, and altered inflammatory pathways in the lung [7]. Other inhalants impacting on the same pathways could in part explain the observed enrichment for smoking DNAme among never-smokers. Methylation of *AHRR* cg05575921 was previously associated with lung function and chronic bronchitis in never-smokers [7]. Maternal smoking, passive smoking and environmental exposures other than cigarette smoking (*e.g*. air pollution) are known to modify DNAme patterns across the genome [26–32]. Maternal smoking during pregnancy has been shown to alter the offspring's DNA markers in a number of genes known to contain smoking-related CpGs [27, 28] and some of these epigenetic patterns, including in *AHRR*, persist to adulthood [29].

From our findings in two well-characterised childhood birth cohorts, there was no evidence for shared common epigenetic mechanisms underlying lung function in adults and children. The comparison was driven by results from the lung function EWAS in adults, given sample size limitations in the available birth cohorts. Lung function in childhood *versus* adulthood is expected to be influenced in part by different biological processes. The nonreplication of the mostly smoking-related lung function DNAme signals might reflect the nonsmoking status of the children and adolescents. Our findings in SAPALDIA point to a dose–response effect of smoking history and intensity on the smoking index. Effects of maternal exposure *in utero*, passive smoking or other inhalants on smoking DNAme are likely smaller than the effects of active smoking [30]. Our EWAS findings generally showed an age-related increase in number and strength of DNAme–lung function associations in adults, despite covariate adjustment for age, as also observed by others [6]. This result is consistent with the observed dose–response effect of smoking and possibly other inhalants on DNAme. However, the inherent interdependency of lung function decline, cumulative smoking exposure and DNAme with ageing prohibits attributing associations to single factors.

A systematic review of peripheral DNAme associated with lung function in population-based cohorts pointed to the lack of consistent evidence [5]. Epigenome-wide DNAme profiling studies of lung tissue suggested DNAme in genes such as *NOS1AP*, *TNFAIP2* and *CHRM1* to be associated with COPD [13, 14]. An EWAS meta-analysis, adjusted for smoking status and pack-years, identified differential DNAme related to COPD and lung function in Koreans. Five loci (*CTU2*, *USP36*, *ZNF516*, *KLK10* and *CPT1B*) were associated with at least two respiratory traits [12]. Evidence of associations in the current EWAS was only observed for 12 out of 376 CpGs associated with lung function phenotypes in these previous studies. This inconsistency may be due to differences in population ancestry, disease status, exposure status, tissue-specific methylation or covariate adjustment. Furthermore, limited sample size and false discovery findings could contribute to nonreplication, as could the absence of post-bronchodilation lung function in the current EWAS. However, our results confirm the associations of two recently published population-based reports [4, 6] investigating smoking, DNAme and lung function. Both reports and our results reveal the same smoking CpGs as prominent signals.

The strength of this EWAS investigation is the robust and extensive study design with availability of repeat measures of DNAme and spirometry data in the same cohort participants, as well as its population-based design. The utilisation of a multilevel analysis scheme, including cross-sectional and longitudinal EWAS analyses at two time-points in the same participants, and EWAS with and without smoking adjustment in all participants and in never-smokers, allowed for a better understanding of lung function DNAme being affected by ageing and smoking. The lung function-associated smoking index derived is building on robust evidence that DNAme in blood is correlated with DNAme and gene expression in lung tissue [4, 23, 33], and that it is a valid biomarker for capturing the effect of smoking on DNAme in the lung [7, 20].

There are several limitations to this study. Limitations in sample size may explain the inability to find association signals in never-smokers and therefore signals common to lung function in childhood and adulthood. The estimation of decline in lung function from only two spirometry time-points is likely to misclassify decline. Additionally, not all replication cohorts had data available for more than one time-point. Pre-bronchodilation lung function is less robust than post-bronchodilator values and may increase variability of the findings. The meta-analysed EWAS results of the cross-sectional analyses showed evidence of inflation (inflation factor λ>1.1) indicating insufficient genomic control; however, adjusting for genomic inflation did not alter our main results. The relevance of the smoking index derived from CpGs in or close to lung function GWAS genes can be questioned given evidence on the complex *trans*-regulation of gene expression [34].

In conclusion, our agnostic investigation shows that DNAme at CpGs related to smoking behaviour are the predominant signals associated cross-sectionally and prospectively with lung function in adults. The findings stimulate further research into the involvement of smoking-related CpGs in lung function-relevant mechanisms and potentially their role as exposure markers beyond active smoking. From our EWAS results it has become clear that larger samples are required to confidently identify CpGs involved in lung function and its age-related decline in persons who never smoked.

## Supplementary material

10.1183/13993003.00457-2019.Supp1 **Please note:** supplementary material is not edited by the Editorial Office, and is uploaded as it has been supplied by the author.
Supplementary material ERJ-00457-2019.Supplement
Spreadsheet containing additional tables S3, S4, S5, S9 and S19. ERJ-00457-2019.Additional_Tables
