# Supplementary material for: Epigenome-wide association study of lung function level and its change
Source: Eur Respir J. 2019 Jul 4;54(1):1900457. doi: 10.1183/13993003.00457-2019 (PMC6610463; doi:10.1183/13993003.00457-2019)
Supplement: Supplementary file 1 [file ERJ-00457-2019.Supplement.pdf]

Online Supplement to

Epigenome-wide association study of lung function level and its change.

Medea Imboden*, Matthias Wielscher*, Faisal I. Rezwan*, André F.S. Amaral*, Emmanuel Schaffner, Ayoung Jeong, Anna Beckmeyer-Borowko, Sarah E. Harris, John M Starr, Ian J. Deary, Claudia Flexeder, Melanie Waldenberger, Annette Peters, Holger Schulz, Su Chen, Shadia Khan Sunny, Wilfried J.J. Karmaus, Yu Jiang, Gertraud Erhart, Florian Kronenberg, Ryan Arathimos, Gemma C. Sharp, Alexander John Henderson, Yu Fu, Päivi Piirilä, Kirsi H. Pietiläinen, Miina Ollikainen, Asa Johansson, Ulf Gyllensten, Maaike de Vries, Diana A. van der Plaat, Kim de Jong, H. Marike Boezen, Ian P. Hall, Martin D. Tobin, Marjo-Riitta Jarvelin*, John W. Holloway*, Deborah Jarvis*, and Nicole M. Probst-Hensch*.

* These authors have contributed equally.

Contents

1. Description of cohorts 4
  1.1. Discovery cohort: ECRHS - European Community Respiratory Health Survey 4
  1.2. Discovery cohort: NFBC1966 - Northern Finland Birth Cohort 1966 4
  1.3. Discovery cohort: SAPALDIA - Swiss Study on Air Pollution Heart and Lung Disease in Adults 5
  1.4. Replication cohort: ALSPAC - Avon Longitudinal Study of Parents and Children 6
  1.5. Replication cohort: FTC - Finnish Twin Cohort 7
  1.6. Replication cohort: IOWBC – Isle Of Wight Birth Cohort 8
  1.7. Replication cohort: KORA - Cooperative Health Research in the Augsburg Region Study 9
  1.8. Replication cohort: LBC1936 - Lothian Birth Cohort 1936 9
  1.9. Replication cohort: LifeLines Cohort 10
  1.10. Replication cohort: NSPHS - Northern Sweden Population Health Study 10
  1.11. Data availability statement 11
  1.12. Childhood replication cohorts: Characteristics of ALSPAC and IOWBC 12
2. Methods: Study design and participants 13
  2.1. Study population 13
  2.2. Figure S1: Selection of study participants in discovery cohorts and data availability in replication cohorts 14
  2.3. Discovery and replication data availability 15
  2.4. Whole blood sample collection and DNA methylation typing in discovery cohorts 15
  2.5. QC- processing of DNA methylation data 15
  2.6. Table: Methylation data quality control processing and sample exclusion in discovery cohorts. 16

2.7. *Spirometry and epidemiological data collection* ... 16
2.8. Table: Use of spirometers in discovery cohorts. ... 17
3. Methods: Statistical analysis ... 17
3.1. *Statistical models* ... 17
3.2. *Generation of smoking scores* ... 20
3.3. Annotation of genomic loci of replicated CpGs ... 20
3.4. Pathway analyses of replicated CpGs ... 21
3.5. Smoking enrichment analysis ... 21
3.6. Replication of mediation analysis in SAPALDIA ... 21
3.7. Two-sample Mendelian randomization study of the replicated CpGs ... 22
4. Results: Sentinel CpG marker selection for replication ... 23
4.1. Table S1: List of sentinel CpGs for replication, all participants ... 23
4.2. Table S2: List of sentinel CpGs for replication, never smokers ... 27
5. Results: Cross-sectional associations with each lung function measure, without smoking adjustment ... 28
5.1. Table S3 (FEV_1/FVC), Table S4 (FEV_1) and Table S5 (FVC) ... 28
5.2. Figure S2: Quantile-Quantile plot of cross-sectional EWAS on FEV_1 and FVC ... 29
6. Results: $EWAS_{repeat}$ - Repeat cross-sectional EWAS in the same study participants – combining the data from both time points ... 30
Box1 Figure: $EWAS_{repeat}$ Manhattan and QQ plots, in all participants ... 31
6.1. Table S6: Repeat cross-sectional associations with lung function in all participants ($EWAS_{repeat}$, M_{base}) ... 32
7. Results: Effect of smoking adjustment on the cross-sectional EWAS (Ms_{mok}): ... 34
7.1. Figure S3: Quantile-Quantile plots of smoking adjusted EWAS, in all participants. ... 35
7.2. cg16288101: Smoking adjusted meta-analyses of cross-sectional associations (M_{smok}), in all participants. ... 36
8. Results: Prediction of DNAme1 association with lung function ($EWAS_{predict}$) ... 37
8.1. Table S8: Prediction EWAS (M_{base}) for change in FEV_1 and for FVC, in all participants ... 37
8.2. Figure S4: Plots of prediction EWAS on FEV_1 and FVC ... 39
9. Results: EWAS meta-analyses in never smokers ... 40
9.1. Figure S5: Quantile-Quantile plots for cross-sectional associations, in never smokers. ... 40
9.2. Table S9: Cross-sectional associations, in never smokers. ... 41
9.3. Table: $EWAS_{repeat}$ in never smokers: ... 42
9.4. Table S10: Prediction EWAS, in never smokers. ... 43
10. Results: Functional characterization of replicated CpGs ... 44
10.1. Table S11: 43 differentially methylated loci associated with lung function ... 44

10.2. Table S12: Functional annotation: Transcription binding sites..45

10.3. Table S13: Functional annotation: Chromatin State Models..45

10.4. Table S14: Functional annotation: Canonical Pathways...46

10.5. Table S15: Functional annotation: Pathway analysis using KEGG..................................46

10.6. Table S16: Functional annotation: GO term enrichment analysis..................................47

11. Results: Enrichment analysis of smoking-related CpGs in discovery EWAS. 47

11.1. Table S17: Overrepresentation of smoking-related CpGs among the lung function associated CpG markers...47

12. Results: Associations of sentinel CpGs with lung function in childhood cohorts (ALSPAC, IOWBC) 48

12.1. Table S18: Associations of sentinel CpGs with FEV_1, FEV_1/FVC, and FVC in childhood birth cohorts, ALSPAC and IOWBC...48

13. Results: ALEC discovery meta-analysis look-up results of CpGs previously associated with respiratory traits 50

13.1. Table S19: Results for cross-sectional adjusted associations (M_{base}) with FEV_1, FEV_1/FVC and FVC at time point 2 50

14. Results: Two-sample Mendelian Randomization look-up in publicly available databases 50

14.1. Table S20: Two-sample MR results for FEV_1 predicted percentage and for FVC.............50

15. Results: Mediation analysis and Mediation smoking index (Mediation-SI)................................52

15.1. Table S21: List of smoking effect mediating candiate CpGs and CpG list of mediation smoking index (Mediation-SI)...52

15.2. Table S22: Replication of mediation analyses on FEV1 and on FVC in SAPALDIA 53

15.3. Figure S6: Association of Mediation-SI with FEV_1 and comparison with association of packyears SI with FEV_1 54

15.4. Figure S7: Association of Mediation-SI with FVC and comparison with association of packyears with FVC...55

15.5. Table S23: Mediation-SI association with FEV1 and with FVC by smoking strata 56

15.6. Figure S8: Mediation-SI association with FEV_1 in ever - and never smokers....................57

16. Results: Scatter plots of percent methylation with lung function outcomes of 57 replicated CpGs 58

16.1. Figure S9: Scatter plots visualizing the relationship between percent methylation (β-value, X-axis) with FEV1/FVC (ratio, y-axis) in SAPALDIA at time point 2..58

16.2. Figure S10: Scatter plots visualizing the relationship between percent methylation (β-value, X-axis) with FEV1 (L, y-axis) in SAPALDIA at time point 2..59

16.3. Figure S11: Scatter plots visualizing the relationship between percent absolute methylation (β-value, X-axis) with FVC (L, y-axis) in SAPALDIA at time point 2 60

17. Results: Candidate Gene based DNAme smoking index (lung-function-gene-SI).........................61

17.1. Table S24: List of CpGs contributing to lung-function-gene-SI.......................................61

17.2. Table S25: Associations of lung-function-gene-SI with FEV_1, FEV_1/FVC and FVC 62
18. Acknowledgments & Funding of cohorts: 63
18.1. Discovery cohort: ECRHS - European Community Respiratory Health Survey 63
18.2. Discovery cohort: NFBC1966 - Northern Finland Birth Cohort 1966 64
18.3. Discovery cohort: SAPALDIA - Swiss Study on Air Pollution Heart and Lung Disease in Adults 64
18.4. Replication cohort: ALSPAC - Avon Longitudinal Study of Parents and Children 65
18.5. Replication cohort: FTC - Finnish Twin Cohort 65
18.6. Replication cohort: IOWBC – Isle Of Wight Birth Cohort 66
18.7. Replication cohort: KORA - Cooperative Health Research in the Augsburg Region Study66
18.8. Replication cohort: LBC1936 - Lothian Birth Cohort 1936 67
18.9. Replication cohort: LifeLines Cohort 67
18.10. Replication cohort: NSPHS - Northern Sweden Population Health Study 67
19. References 68

1. Description of cohorts

1.1. Discovery cohort: ECRHS - European Community Respiratory Health Survey

Study description

The European Community Respiratory Health Survey (ECRHS) is an international multicentre cohort study designed to assess the prevalence of asthma and allergic disease and identify their risk factors.[1] Young adults of European descent were randomly recruited from community-based sampling frames in the ECRHS I (1991-1993) and followed up twice in the 20 years after the first assessment (ECRHS II: 1998-2002; ECRHS III: 2008-2013), for full protocols, see http://www.ecrhs.org). The study was approved by the local ethics committees in each region: Reykjavík, Iceland (The National Bioethics Committee, Reykjavík, Iceland); Umea, Uppsala, Gothenburg, Sweden (Regional Ethical Committee in Uppsala, Sweden); Erfurt, Hamburg, Germany (Ethic Committee of the Bavarian State Chamber of Physicians, Germany); Norwich, UK (Norwich District Ethics Committee); Ipswich, UK (Ipswich–East Suffolk Local Research Ethics Committee); Grenoble, France (Ethics committee Paris Bichat-Claude Bernard); Barcelona, Spain (Comité Ético de Investigación Clínica del Instituto Municipal de Asistencia Sanitaria, Barcelona, Spain); Albacete, Spain (Comité de Ética e Investigación de Complejo Hospitalario de Albacete, Spain); Oviedo, Spain (Comité Ético de Investigación Clínica Regional, Hospital Universitario Central de Asturias, Oviedo, Spain); Galdakao, Spain (Comité Ético de Investigación del Hospital de Galdakao, Spain); and Basel, Switzerland (Swiss Academy of Medical Sciences and the ethics committee of Basel).

1.2. Discovery cohort: NFBC1966 - Northern Finland Birth Cohort 1966

Study description

The Northern Finland Birth Cohort 1966 (NFBC 1966) is an unselected, population-based sample of all live births in 1966 (n = 12,058) in the provinces of Oulu and Lapland in Finland. Women with expected delivery dates in 1966 were recruited through maternity health centers.[2] In 1997, at offspring age of 31 years, all cohort participants with known addresses were sent a postal questionnaire on health and lifestyle and those living in Northern Finland or Helsinki area were invited to a clinical examination which included blood sampling. In total, both questionnaire, clinical and biological data were collected for 6,007 participants. DNA was successfully extracted for 5,753 participants from fasted blood samples[3]. In 2012, all individuals with known address in Finland were sent postal questionnaires and an invitation for clinical examination. Both questionnaire, clinical and biological data were collected for 5,539 participants. DNA methylation at 31 years was extracted for 807 randomly selected subjects of whom questionnaire, clinical and genetic data were available at both 31 and 46 years. Of them analyses here included 611 subjects with lung function (spirometry both at 31 and 46 years of age), other relevant covariate and methylation data.

This longitudinal, epidemiological research program is maintained within the Department of Health Sciences, Faculty of Medicine, University of Oulu, Finland (http://www.oulu.fi/nfbc/). Informed consent for the use of the data including DNA was obtained from all subjects. The study was approved by the ethics committees in Oulu (Finland) and Oxford (UK) universities in accordance with the Declaration of Helsinki.

Methylation of genomic DNA was quantified using the Infinium HumanMethylation450 BeadChip (Illumina, Inc.) array at age 31 years and The Infinium Methylation EPIC array (Illumina, Inc.) at age 46 years according to manufacturer's instructions. Bisulfite conversion of genomic DNA was performed using the EZ DNA methylation kit according to manufacturer's instructions (Zymo Research, Orange, CA). In NFBC1966 quality control and quantile normalization for DNA methylation data was adapted from the CPACOR pipeline.[4] Illumina Background Correction was applied to the intensity values, a detection p-value threshold was set at $p < 10^{-16}$, and samples with call rate < 98 % were excluded. Probes with call rate < 95 % were excluded from the analyses.

1.3. Discovery cohort: SAPALDIA - Swiss Study on Air Pollution Heart and Lung Disease in Adults

Study description

SAPALDIA was initiated in 1991 to specifically study the air pollution impact on respiratory health.[5, 6] It is a population-based cohort in Switzerland recruiting subjects aged 18 to 60 from population registries in eight communities, representing the three largest language groups (German, French, Italian) as well as different levels of air pollution and degrees of urbanization**.** Subjects underwent spirometry and answered a detailed questionnaire on respiratory health, allergies, smoking history, and lifestyle factors in the baseline (year 1991, SAPALDIA 1), follow-up (year 2002, SAPALDIA 2) and second follow-up (year 2010, SAPALDIA 3) examination. The study is in agreement with the Declaration of Helsinki. Participants provided prior written informed consent and ethical approval for the study was given by the Overall Regional Ethics Commission for Clinical Medicine (Swiss Academy of Medical Sciences) and by the respective cantonal ethical committee for each survey.

1.4. Replication cohort: ALSPAC - Avon Longitudinal Study of Parents and Children

Study description

The Avon Longitudinal Study of Parents and Children (ALSPAC) recruited 14,541 pregnant women with expected delivery dates between April 1991 and December 1992.[7, 8] Of these initial pregnancies, there were 14,062 live births and 13,988 children who were alive at 1 year of age. When the oldest children were approximately 7 years of age, an attempt was made to bolster the initial sample with eligible cases who had failed to join the study originally. As a result, when considering variables collected from the age of seven onwards (and potentially abstracted from obstetric notes) there are data available for more than the 14,541 pregnancies mentioned above. The study website contains details of all the data that are available through a fully searchable data dictionary (http://www.bris.ac.uk/alspac/researchers/data-access/data-dictionary).

DNA methylation data and QC and preprocessing

As part of the ARIES project (http://www.ariesepigenomics.org.uk), a sub-sample of 1,018 ALSPAC mother–child pairs had DNA methylation measured using the Infinium HumanMethylation450 BeadChip (Illumina, Inc.). DNA methylation data was measured in three samples per child, from cord blood and venous blood samples at age 7 and again at age 15 or 17 years. All DNA methylation wet-lab and preprocessing analyses were performed at the University of Bristol as part of the ARIES project and has been described in detail previously.[9]

In detail, peripheral blood was collected according to standard procedures, spun and frozen at -80˚C. DNA methylation analysis and data pre-processing were performed at the University of Bristol as part of the ARIES project (ariesepigenomics.org.uk). Following extraction, DNA was bisulfite converted using the Zymo EZ DNA MethylationTM kit (Zymo, Irvine, CA). Following conversion, the genome-wide methylation status of over 485,000 CpG sites was measured using the Illumina Infinium® HumanMethylation450k BeadChip assay according to the standard protocol. The arrays were scanned using an Illumina iScan and initial quality review was assessed using GenomeStudio (version 2011.1). The level of methylation is expressed as a "Beta" value (β-value), ranging from 0 (no cytosine methylation) to 1 (complete cytosine methylation). Samples from all time-points in ARIES were distributed across slides using a semi-random approach (sampling criteria were in place to ensure that all time-points were represented on each array) to minimize the possibility of confounding by batch effects. Samples failing quality control (average probe detection p-value ≥ 0.01) were repeated. As an additional quality control step genotype probes on the HumanMethylation450k were compared between samples from the same individual and against SNP-chip data to identify and remove any sample mismatches. Data were pre-processed in R (version 3.0.1) with the WateRmelon package according to the subset quantile normalization approach described by Touleimat & Tost in an attempt to reduce the non-biological differences between probes. We removed probes that had a detection P-value >0.05 for >5% of samples, probes on the X or Y chromosomes and SNPs (rs probes).

Proportions of cell types were estimated from DNA methylation data using the estimateCellCounts function in the minfi R package which is based on the method developed by Houseman et al. .[10, 11] This estimated the proportion of B cells, CD8 T cells, CD4 T cells, granulocytes, eosinophils, neutrophils, NK cells and monocytes at the 7.5 year methylation time-point and at the 16.5 year methylation time-point independently. Ten surrogate variables were generated and included in

models to adjust for technical batch in cross-sectional and prediction models, using the sva R package. In longitudinal models, twenty surrogate variables were used, ten from each of the two methylation time points. Asthma status at 7.5 years of age was defined from questionnaires completed by the mothers of the study children at approx. 7.5 years of age when they were asked if their study child have ever been diagnosed by a doctor with asthma. Mothers were also asked in the same questionnaire if their study child had taken any asthma medicine in the past 12 months. The mothers of the study children were asked the same questions about asthma doctor diagnosis and asthma medications in the past 12 months when study children were 14 years old.

Lung function and covariates

Spirometry was done in a research clinic at ages 8.5 and 15 years approximately by using methods described previously.[12] Lung function at 15 years was defined as the highest of 3 measures before administration of salbutamol (pre-salbutamol measures) and 15 minutes after receiving 400 mg of salbutamol (post-salbutamol measures) administered by using metered aerosol and a spacer.

Maternal education (proxy for social class) was classified for this study as 1"University education", 2"A-level" or 3"O-level or lower" based on questionnaires completed by the study mothers. We derived smoking status from questionnaires completed by the study children at approximately 16 years of age. Study participants were asked if they had ever smoked a cigarette. Those responding Yes to the smoking question were then asked about their smoking frequency, indicating either 1"I have only ever tried smoking cigarettes once or twice" , 2"I used to smoke sometimes but I never smoke cigarettes now", 3"I sometimes smoke cigarettes but I smoke less than one a week", 4"I usually smoke between one and six cigarettes a week", 5"I usually smoke between one and six cigarettes a week but not every day" and 6"I usually smoke one or more cigarettes every day".

Height and weight were measured at research clinic attendance at the same ages as lung function assessment was carried out. During clinic attendance, height was measured to the last complete mm using a Harpenden Stadiometer and weight was measured using a Tanita Body Fat Analyser (Model TBF 305; Tanita Europe Ltd, Amsterdam, The Netherlands).

1.5. Replication cohort: FTC - Finnish Twin Cohort

Study description

The Finnish Twin Cohort (FTC) study was initiated in 1974 to study genetic and environmental factors contributing to complex diseases and behavioral risks.[13] The study participants were recruited from the FinnTwin16 cohort,[13] a population-based, longitudinal study of five consecutive birth cohorts (1975–1979) of twins, their siblings, and their parents. The FinnTwin16 cohort was established in 1991, and the first assessments took place when the twins were 16 years of age, with four waves of follow-up when the twins were 17, 18.5, 24,[14] and 34 years,[13] on average. We studied young adult twin individuals who were selected by their responses to questions on weight and height at the age of 23–36 years to represent a wide range of intra-pair differences in body mass index (BMI).

The spirometric examinations were performed during 2004-2013 by a mass flow sensor (Vmax encore, Sensormedics, Palm Springs, CA, USA). The flow device was cleaned before calibration by the

device's automatic cleaning program, after which 0 calibration was performed. Flow calibration was then performed with a 3 liter pump between flow values 0.5 l/s and 6 l/s, after which a volume calibration was performed. During spirometry, patient was sitting with the nose closed with a clip, and at least 3 maximal flow volume curves were measured, the difference of best two FEV1 values or FVC values had to be less than 150 ml, and the expiration should last at least 6 seconds. If this was not fulfilled, additional measurements were performed. The spirometric results were given according to ERS/ATS recommendation from 2005.[15] Forced vital capacity (FVC), forced expiratory volume in one second (FEV1) and FEV1/FVC ratio were measured. The 2012 multiethnic reference values were used to compare the spirometric results.[16] The study subjects provided written, informed consent. The protocol was designed and performed according to the principles of the Helsinki Declaration and was approved by the Ethics Committee of the Hospital District of Helsinki and Uusimaa.

DNA methylation data

DNA extracted from white blood cells of 308 FinnTwin16 twins (112 MZ and 42 DZ pairs) aged 23-36, was used to generate DNA methylation data by Infinium HumanMethylation450 BeadChip (Illumina, Inc.). All DNA methylation wet-lab analyses were performed at the Norwegian Genomics Consortium, Oslo, Norway, and data preprocessing analyses were performed at the University of Helsinki, Finland, as part of the ongoing FTC projects, and has been described in detail previously.[17] For the current study, 110 MZ twins (55 MZ pairs) with both DNA methylation and spirometry data available from the same time point were selected.

1.6. Replication cohort: IOWBC – Isle Of Wight Birth Cohort

Study description

The IOWBC is a single-centre study designed to represent the community population. All children born on the Isle of Wight in a defined period (January 1989 to February 1990) were eligible for inclusion. The cohort was recruited through the 1509 women who gave birth to 1536 children on the IOW during the recruitment period. The children in the IOWBC have been seen on six occasions over the course of 26 years, at 1, 2, 4, 10, 18 and 26 years.[18] The focus of the IOWBC is to investigate the etiology and natural history of asthma and allergic disease manifestations in an unselected population during childhood and early adult life. Spirometry data was performed at follow-ups at age 10, 18 and 26. The spirometer used at each follow-up was Koko spirometer and software with a portable desktop device (both PDS Instrumentation, Louisville,KY, USA).[19]

DNA methylation data

Biologic sample collection of peripheral blood was obtained at follow-ups at the age 10 and 18 and the samples of a subgroup were used for DNA methylation typing. 817 samples were typed using the Illumina Infinium® HumanMethylation450k BeadChip assay according to the standard protocol.[19] Methylation data was processed using standard QC-pipelines (CPACOR, quantile normalization and ComBat) using Minfi and SVA R packages (R version 3.51).

1.7. Replication cohort: KORA - Cooperative Health Research in the Augsburg Region Study

Study description

The Cooperative Health Research in the Augsburg Region Study, KORA, aims to gain new insights into the causes, development and consequences of cardiovascular disease, diabetes as well as lung diseases and allergies.[20-22] The KORA S4 survey is an independent population-based sample from the general population living in the region of Augsburg, Southern Germany. KORA S4 was conducted in 1999/2001 and standardized examinations were applied in the survey (4261 participants). A total of 3080 subjects participated in a follow-up examination of S4 in 2006–08 (KORA F4), comprising individuals who, at that time, were aged 32–81 years. A subset of 1321 subjects, aged 44-64 years, underwent spirometry and were followed up in the KORA FF4 survey 7 years later (2013/2014). Of those, a total of 628 subjects had DNA methylation data from blood samples collected at KORA F4 and data available on spirometry from KORA F4 and KORA FF4.

DNA methylation data

DNA methylation was measured in DNA extracted from whole blood of the participants using the Infinium HumanMethylation450K BeadChip at the Helmholtz Zentrum München, Research Unit Molecular Epidemiology and Genome Analysis Centre. The bisulfite conversion and genome-wide methylation assessment were performed as previously described.[23, 24]

QC and preprocessing

Normalization of the methylation data was conducted following the CPACOR pipeline,[4] beginning with exclusion of 65 single nucleotide polymorphism markers and background correction using the R package minfi.[10] Probes were set to NA if the detection p-value ≥0.01 or number of beads ≤3. Samples were excluded if the detection rate was ≤0.95. Quantile normalization was then performed on the signal intensities. The methylation of a given cytosine was first calculated as a β-value, the ratio of the methylated signal intensity to the sum of the methylated and unmethylated signal intensities. Following normalization, CpG sites with a detection rate below 95% were excluded. To reduce possible impact of non-biological effects, we adjusted the methylation values for technical effects prior to analysis. In detail, principal component analysis was performed on the intensities of all (non-negative, autosomal) control probes after background correction. We then modeled the methylation values of each CpG site across all samples as a function of the first 20 principal components. Residuals of these models were used as “technically adjusted” methylation values for all analyses.[4]

1.8. Replication cohort: LBC1936 - Lothian Birth Cohort 1936

Study description

The Lothian Birth Cohort 1936 (LBC1936) comprises 1,091 community-dwelling individuals who agreed to participate in a longitudinal study of cognitive ageing starting at mean age about 70 years.[25-28] At age 11 years, almost all of them took part in the Scottish Mental Survey of 1947, which employed the Moray House Test No. 12, a test of general cognitive ability. At recruitment in older age (at age 70 years), between 2004 and 2007, subjects agreed to cognitive testing and other medical, physical and psychosocial assessments. Further waves of testing occurred at ages 73

(wave2), 76 (wave 3) and 79 years (wave 4). Lung function was measured at each wave using a Micro Medical Spirometer. For the purposes of this study spirometry measures at ages 70, 73 and 76 years were used.

DNA methylation data

Blood samples for methylation were taken from participants at each wave of testing. DNA methylation was measured at 485,512 sites using the Infinium HumanMethylation450 BeadChip array, at the Edinburgh Clinical Research Facility Genetics Core, Western General Hospital, Edinburgh. DNA methylation data, spirometry measures and covariates were available for 449 individuals at waves 1, 2 and 3.

1.9. Replication cohort: LifeLines Cohort

Study description and DNA methylation data

The LifeLines cohort study is a large Dutch population-based cohort study designed to investigate chronic diseases and healthy aging.[29, 30] Detailed information about LifeLines can be obtained at the official website (http://www.lifelines.net). A subgroup of 1,656 participants of the LifeLines cohort was non-random selected based on lung function, smoking status and exposure to environmental exposures. Whole blood samples collected for DNA extraction and DNA methylation level for each CpG site was measured using the IlluminaInfinium® Human Methylation 450K array (Illumina,Inc.) at the UMCG, Groningen, The Netherlands.

1.10. Replication cohort: NSPHS - Northern Sweden Population Health Study

Study description

The Northern Sweden Population Health Study (NSPHS) was initiated to provide a health survey of the population in a geographically remotes area and to study the medical consequences of lifestyle and genetics.[31, 32] According to the Sweden Census, on 31 December 2006, of 826 eligible inhabitants (aged 15 years or older) 740 subjects agreed to participate (90%) and 656 subjects contributed complete data, resulting in a final sample of 347 (53%) women and 95 (14.5%) individuals with a traditional lifestyle. The comprehensive collection of data included genealogy, socio-demography, body size, blood samples for clinical chemistry, medical history of participants and family members, and lifestyle. Spirometry was performed in a sitting position without nose clips using a Spida 5 spirometer (MicroMedical; http://www.medisave.co.uk). Three consecutive lung function measurements per participant were performed and the maximum value per measured lung function parameter was used for further analysis. Within the scope of this article, Peak Expiratory Flow (PEF), Forced Expiratory Volume (FEV), and Forced Vital Capacitiy (FVC) were analyzed. Years of spirometry: 2006 and 2009 (same time as blood samples were taken)

DNA methylation data

- Number of samples typed: 732
- Type of biologic sample used to extract DNA: Whole blood

- Type of Infinium HumanMethylation BeadChip (Illumina, Inc.) used /Array used: Illumina 450K
- Institution in which wet-lab and preprocessing analyses were performed : DNA methylation analyses were performed by the SNP & SEQ Technology Platform at Science for Life Laboratory (SciLifeLab), Sweden

1.11. Data availability statement

Statistical codes, and full discovery and replication EWAS effect estimates (meta-analysed and cohort-specific) are made publically available with no end date on the public repository DRYAD (http://datadryad.org/) at the time of publication. Access restrictions apply to the individual methylome data underlying the analysis. The consent given by cohort participants does not cover data sharing in public data repositories. Data requests for methodologically sound research proposal can be addressed through the cohorts' websites to the data access committee or directly to the principal investigators of the epidemiologic studies.

ECRHS	d.jarvis@imperial.ac.uk
NFBC1966	m.jarvelin@imperial.ac.uk
SAPALDIA	nicole.probst@swisstph.ch
ALSPAC	A.J.Henderson@bristol.ac.uk
FTC	miina.ollikainen@helsinki.fi
IOWBC	Details on data access are available at: http://www.allergyresearch.org.uk/studies/birth-cohort/#cohort-data-use. Contact Mr Stephen Potter: stephen.potter@iow.nhs.uk
KORA	Application for KORA data can be made via the KORA *P*roject *A*pplication *S*elf-*S*ervice *T*ool, KORA.PASST, at https://epi.helmholtz-muenchen.de/.
LBC1936	Sarah.Harris@igmm.ed.ac.uk; I.Deary@ed.ac.uk
LifeLines	h.m.boezen@umcg.nl
NSPHS	ulf.gyllensten@igp.uu.se

1.12. Childhood replication cohorts: Characteristics of ALSPAC and IOWBC

Table: Characteristics of childhood replication cohorts.

	ALSPAC Child	ALSPAC Teens	IOWBC Time point 1	IOWBC Time point 2
**N**	258	258	162	218
**Female, %**	43.41	43.41	43.8	52.3
**Age (years), mean (SD)**	8.54 (0.14)	15.35 (0.18)	10 (0)	18 (0)
**Height (cm), mean (SD)**	132.4 (5.8)	170.5 (8.8)	139.2 (6.4)	171.5 (9.6)
**Weight (kg), mean (SD)**	30.0 (5.6)	61.7 (11.3)	35.1 (7.2)	68.5 (13.8)
**Body mass index, (kg/m2) mean (SD)**	17.0 (2.3)	21.2 (3.1)	18.0 (2.8)	23.2 (4.1)
**Smoking status, %**				
Never smoker*	100	100	100	100
**Tobacco smoker exposure during childhood or in utero†, %**	60.0	60.0	56.2	72.9
**Education‡**	42.3	42.3	12;43;20;24;1	12;41;21;25;1
**FVC (L), mean (SD)**	1.9 (0.3)	3.8 (0.9)	2.3 (0.4)	4.7 (0.8)
**FEV1 (L), mean (SD)**	1.7 (0.3)	3.4 (0.8)	2.0 (0.3)	4.1 (0.8)
**FEV1/FVC, mean (SD)**	0.89 (0.06)	0.91 (0.07)	0.88 (0.06)	0.87 (0.07)
**Airflow obstruction (FEV1/FVC<0.7), %**	1.16	1.55	0	1.8
**Doctor-diagnosed asthma, %**	20.0	22.7	14.8	16.5
**Respiratory medication, % (% missing values)¶**	13.1 (2.3)	12.2 (17.4)	31.5 (0)	12.8 (3.2)

Footnote to childhood cohort characteristics table:

* Analysis restricted to non-smokers.

† ALSPAC: In utero tobacco smoke exposure is defined as mothers that reported any smoking at either 18wks OR 32 wks of pregnancy. Passive smoke exposure is defined as the child living in the same house as a smoker, as reported by the mothers at approximately 65 months of age. IOWBC: Tobacco smoke exposure reported for time point 1: 0 to 10 years of age and for time point 2: 0-18 years of age.

‡Education: Maternal Education used in ALSPAC [O-level or lower]; education at age of 18 used in IoW [very low;low;medium;high;other]

¶ Asthma medication use in the last 12 months.

2. Methods: Study design and participants

2.1. Study population

The discovery cohorts were three well-characterized longitudinal cohort studies which were part of the Aging Lungs in European Cohorts (ALEC) project. Briefly, two discovery cohorts were population-based studies specifically designed to investigate respiratory health, the European Community Respiratory Health Study (ECRHS) (Burney et al. 1994; Janson et al. 2001) and the Swiss Study on Air Pollution Heart and Lung Disease in Adults (SAPALDIA) (Ackermann-Liebrich et al. 1997; Martin et al. 1997) and the third discovery cohort was a birth cohort, the Northern Finland Birth Cohort 1966 (NFBC1966). As discovery dataset, we used the biological samples and the epidemiological data of two consecutive follow-up surveys roughly 10 years apart for ECRHS and SAPALDIA and 15 years apart for NFBC1966. The replication cohorts comprised five adult inception cohorts and two childhood birth cohorts. The adult replication cohorts included three with population-based design: the Cooperative Health Research in the Augsburg Region Study (KORA), the North Sweden Population Health Study (NSPHS)) and the LifeLines cohort study with enriched ascertainment of smokers (LifeLines); one study of adults with a birth cohort design: the Lothian Birth Cohort 1936 (LBC1936)), and one with twin cohort design: the Finnish Twin Cohort study (FTC). The childhood replication cohorts included two birth cohorts: the Avon longitudinal Study of Parents and Children (ALSPAC) and the Isle of Wight Birth Cohort (IOWBC) cohort. All cohorts comply with the Declaration of Helsinki and ethical approval had been obtained from the respective national and regional ethical review committees.

2.2. Figure S1: Selection of study participants in discovery cohorts and data availability in replication cohorts

Figure S1: Discovery cohorts: Flow chart of the selection of study participants. Replication cohorts: Contribution of each replication cohort to the meta-analyses based on data availability.

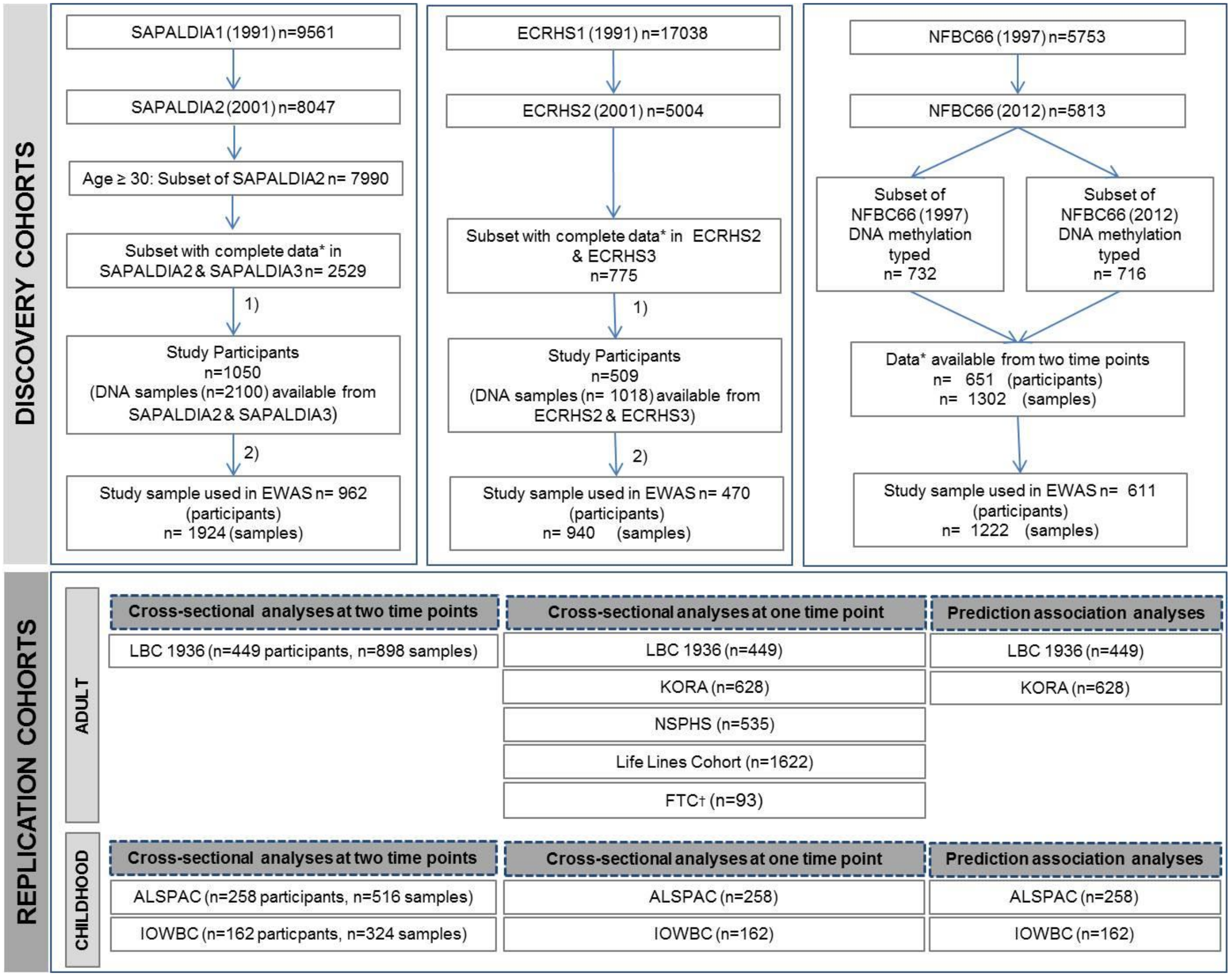

Footnote:

* Study participants with complete data on outcome, covariates, in particular smoking status and packyears information, genome wide genetic information and with peripheral blood samples available for DNA methylation typing. In the discovery cohorts a random sample of study participants aged 30 years or older at the first time point, with complete data on outcome and covariates, as well as blood samples available for DNA extraction at two consecutive follow-up surveys was selected. [1) Selection of study participants to be typed with Infinium® HumanMethylation 450K or 850K Bead Chip (Illumina) based on a random selection procedure. 2) Selection of study participants: DNA methylation data processing related sample quality control criteria (exclusion of samples if call rate <95% or sex mismatch based on XY probes; for SAPALDIA and NFBC1966 detection P-value > 10-16 and for ECRHS detection P-value > 0.05).]

†FTC did not contribute to the combined meta-analyses of cross-sectional associations (using data from the oldest time point available: time point 2 of ECRHS, NFBC1966, SAPALDIA, LBC1936 and time point 1 of KORA, LifeLines and NSPHS). The exclusion of FTC was due to the lower mean age (30.4 years) compared to the other adult cohorts' mean age (ECRHS (54.5 years), NFBC1966 (46.3 years), SAPALDIA (58.8 years), LBC1936 (76.3 years) and the single available time point for KORA (60.1 years), LifeLines (46.7 years) and NSPHS (55.1 years)).

2.3. Discovery and replication data availability

For the current analysis purposes the data availability across eight adult inception cohorts (three discovery and five replication cohorts) was maximal for a one time-point cross-sectional association analysis. Among the replication cohorts, only one adult birth cohort (LBC1936, n=449) and both childhood birth cohorts (ALSPAC, n=258 and IOWBC, n=162) had methylation information and epidemiological data available from two time points. The sample size for the replication of repeat cross-sectional associations in adults was limited to the LBC1936 data. The replication sample size for the prediction EWAS on change in lung function over follow-up time was limited to two cohorts (KORA, n= 628, and LBC1936, n=449). The data available on spirometry was obtained from two follow-ups was 7 years apart for KORA and 6 years apart for LBC1936 and DNA methylation data was obtained from blood samples collected at the time of first spirometry. The remaining replication cohorts had DNA methylation, spirometry and epidemiological data available from only one time point (LifeLines (n=1622), NSPHS (n=535) and FTC (n=93)).

2.4. Whole blood sample collection and DNA methylation typing in discovery cohorts

For discovery cohorts, biological samples of peripheral blood were collected using standard operating procedures at two time points on two consecutive follow-up surveys from which DNA was extracted. Genome-wide DNA methylation typing including DNA bisulphite conversion was performed at the Wellcome Trust Center for Human Genetics (Oxford, UK) for the samples of both time points of SAPALDIA and ECRHS, and for the second time point of NFBC1966. Quantification of DNA methylation across the genome using the Illumina® Infinium Human Methylation technology was obtained successfully using the 850K BeadChip for ECRHS on samples of 509 participants from both time points (1,018 samples) and for NFBC1966 samples collected at the second time point from 766 participant, as well as using the 450K BeadChip for SAPALDIA on samples of 984 participants from both time points (1,968 samples). For ECRHS and SAPALDIA, paired samples of the same participant from both time points were randomized across the methylation arrays to be typed in parallel in order to minimize batch effects. For NFBC, the samples collected at the first time point from 816 participants had been previously typed using the 450K BeadChip. The quality control (QC) and normalization procedures applied in the discovery cohorts are described in table at 2.6 of this Appendix. In total 4,568 samples of 2,259 participants were typed in the discovery cohorts. The methylation data was QC-processed, normalized and correction for technical batch effects were performed separately in each cohort. Cohort specific QC-steps were performed. A call rate of 95% was applied as selection criteria on marker and sample level; we had 2,043 samples for both time points (ECRHS: n=470, NFBC1966: n=611 and SAPALDIA: n= 962). For cohort-specific EWAS analyses, we used all autosomal markers available for each time point. Given the use of two different DNAme typing arrays (850K BeadChip and 450K BeadChip), all cohort-specific EWAS marker results were meta-analysed without restricting to a set of common markers.

2.5. QC- processing of DNA methylation data

We performed the methylation data processing within each cohort separately using R packages minfi,[10] RnBeads,[33] and CPACOR.[4] After standard QC cleaning steps regarding duplicates, sex inconsistency, low sample

quality (sample call rate >95%) for all three cohorts, ECRHS additionally excluded outlier samples beyond 1.5 interquartile range (IQR) and NFBC1966 excluded samples with their 1st PC score of the DNA methylation values outside the interval given by the mean +/- 4SD (see table at 2.6 of this Appendix). Call rate detection P-value was set to $P>10^{-16}$ for NFBC1966 and SAPALDIA, and P>0.05 for ECRHS. Dye-bias correction and absolute methylation level (β-values) were computed using the minfi R-package. Low marker quality exclusion for probes with call rate below 95% was applied, leading to the probe exclusion of 9.5% (n=80'264) and 10.0% (n=85'152) for ECHRS using the 850K BeadChip at first and second time point, respectively; 3.1% (n=14'486, using 450K BeadChip) and 1.72% (n=14'586, using 850K BeadChip) for NFBC1966 at first and second time point, respectively; 0.4% (n=1'894) and 0.37& (n=1'785) for SAPALDIA using the 450K BeadChip at first and second time point, respectively. Beta mixture quantile normalization (BMIQ)[34] of β-values was applied for SAPALDIA methylation data and quantile normalization[35] for ECRHS and NFBC1966. For technical bias correction the first 30 principal components derived from the control probes were used for ECRHS and SAPALDIA methylation data. We excluded methylation markers on sex chromosome and used all remaining markers for cohort-specific EWAS analyses (number of markers for time point 1: n=766'891 (ECRHS2), n=459'378 (NFBC1966 (1997)), n=471'970 (SAPALDIA2); number of markers for time point 1: n=762'003 (ECRHS2), n=832'569 (NFBC1966 (2012)), n=472'079 (SAPALDIA3)). For cohort-specific EWAS analyses, we used all autosomal markers available for each time point and cohort-specific EWAS marker results were meta-analysed without restriction to common markers.

2.6. Table: Methylation data quality control processing and sample exclusion in discovery cohorts.

	ECRHS2	ECRHS3	NFBC1966 (1997)	NFBC1966 (2012)	SAPALDIA2	SAPALDIA3
samples successfully typed	509	509	816	766	984	987
duplicates excluded	0	0	9	8	0	0
95% sample call rate	14	2	67	40	0	0
sex mismatch	7	11	7	1	1	4
outliers*	1	3	1	1	na	na
remaining QC-ed samples	487	493	732	716	983	983

* outlier definition was cohort specific, either >1.5 IQR for ECRHS and SAPALDIA and 1st PC score of the DNA methylation values outside mean +/- 4SD for NFBC1966.

2.7. *Spirometry and epidemiological data collection*

Detailed description of data collection and study design has been previously published for the discovery cohorts: ECRHS,[36, 37] SAPALDIA,[38-40] and NFBC1966,[41] and for the replication cohorts: KORA,[42] LBC1936,[25, 27, 28] LifeLines,[29, 30] NSPHS,[31] ALSPAC,[8, 9]IOWBC,[18] and FTC.[43]

2.8. Table: Use of spirometers in discovery cohorts.

The cohorts used spirometers of different brands and models and change in spirometers between surveys did occur. Spirometric comparability across time points was addressed within cohorts.[38]

Spirometer brand and model	SAPALDIA 2 time point 1	SAPALDIA 3 time point 2	ECRHS 2 time point 1	ECRHS 3 time point 2	NFBC1966 age31 time point 1	NFBC1966 age46 time point 2
SensorMedics 2200	100%	--	--	--	--	--
EasyOne ndd	--	100%	--	--	--	--
SensorMedics 2400	--	--	37.7%	--	--	--
Jaeger Pneumotach	--	--	13.4%	--	--	--
Biomedin Spirometer	--	--	48.9%	--	--	--
NDD EasyOne	--	--	--	100%	--	--
Vitalograph P Model	--	--	--	--	100%	
MasterScreen Pneumo	--	--	--	--	--	100%

3. Methods: Statistical analysis

In the discovery cohorts, two types of smoking score were generated to test their combined effect. Briefly, an ALEC custom SI specific for each lung function outcome (FEV_1, FEV_1/FVC or FVC) was generated from a subset of CpGs selecting known smoking-related markers from the ALEC discovery repeat cross-sectional EWAS 100 top association signals; second a lung-function-gene-SI including smoking associated CpGs located in 18 GWAS-identified lung function candidate genes.

3.1. *Statistical models*

Residuals model

$$residu_{DNAm\,i} = DNAm_{\,i} - (\beta_0 + \sum_{j=1}^{30} \beta_j PC\_cp_{ij})$$

Linear model formulas of 12 statistical models for cross-sectional associations (three outcomes (FEV1/FVC, FEV1, FVC), two time points (T1 and T2) and two covariate adjustments (M_{base}, M_{smok})):

Linear regression model, mathematical formula

$$y_i = \beta_0 + \sum_{j=1}^{p} \beta_j x_{ij} + e_i$$

i=1,2,…,n (n=subjects)

Model for cross-sectional associations, M_{base} adjustment:

$$LF = \beta_0 + \beta_1 residu_{DNAm} + \beta_2 age + \beta_3 age^2 + \beta_4 height + \beta_5 \left(height - mean(height)\right)^2 + \beta_6 sex + \beta_{7_0} area_1 + \cdots + \beta_{7_k} area_k + \beta_8 bmi + \beta_{9_1} educ_1 + \beta_{9_2} educ_2 + \beta_{10}\ Bcell + \beta_{11} CD4T + \beta_{12} CD8T + \beta_{13} Eos + \beta_{14} Mono + \beta_{15} Neu + \beta_{16} NK + \beta_{17} sex * age + \beta_{18} sex * age^2 + \beta_{19} sex * height + \beta_{20} sex * \left(height - mean(height)\right)^2 + e_i$$

All variables are participant's characteristics of the same time point. At T1 all covariates and LF parameter are from T1. At T2, all covariates and LF parameters are from T2.

Model for cross-sectional associations, M_{smok} adjusted:

$$LF = \beta_0 + \beta_1 residu_{DNAm} + \beta_2 age + \beta_3 age^2 + \beta_4 height + \beta_5 \left(height - mean(height)\right)^2 + \beta_6 sex + \beta_{7_0} area_1 + \cdots + \beta_{7_k} area_k + \beta_8 bmi + \beta_{9_1} educ_1 + \beta_{9_2} educ_2 + \beta_{10}\ Bcell + \beta_{11} CD4T + \beta_{12} CD8T + \beta_{13} Eos + \beta_{14} Mono + \beta_{15} Neu + \beta_{16} NK + \beta_{17} sex * age + \beta_{18} sex * age^2 + \beta_{19} sex * height + \beta_{20} sex * \left(height - mean(height)\right)^2 + \beta_{21_1} Smok\_status_1 + \beta_{21_2} Smok\_status_2 + \beta_{22} packyrs + e_i$$

Linear model formulas of 6 statistical models for prediction associations (three outcomes (FEV1/FVC, FEV1, FVC) and two covariate adjustments (M_{base}, M_{smok})):

Linear model for prediction association:

The model is additionally adjusted for the respective lung function parameter at baseline. All covariates are baseline characteristics.

$$\frac{y2_i - y1_i}{T2 - T1} = \beta_0 + \beta_1\ y1_i + \sum_{j=2}^{p} \beta_j x1_{ij} + e_i$$

$$\text{“}annual\ change\ in\ LF\text{”} = \frac{(LF_2 - LF_1)}{(T2-T1)_{in\ year}}\ where\ LF_1 and\ LF_2 are\ the\ Lung\ Function\ parameters\ at\ baseline\ and\ follow-up\ measured\ at\ time\ T1\ and\ T2, respectively.$$

Linear model for prediction associations, M_{base} adjusted:

$$\frac{(LF_2 - LF_1)}{(T2 - T1)_{in\ year}} = \beta_0 + \beta_1 residu_{DNAm} + \beta_2 LF_1 + \beta_3 age + \beta_4 age^2 + \beta_5 height + \beta_6 \left(height - mean(height)\right)^2 + \beta_7 sex + \beta_{8_0} area_1 + \cdots + \beta_{8_k} area_k + \beta_9 bmi + \beta_{10_1} educ_1 + \beta_{10_2} educ_2 + \beta_{11}\ Bcell + \beta_{12} CD4T + \beta_{13} CD8T + \beta_{14} Eos + \beta_{15} Mono + \beta_{16} Neu + \beta_{17} NK + \beta_{18} sex * age + \beta_{19} sex * age^2 + \beta_{20} sex * height + \beta_{21} sex * \left(height - mean(height)\right)^2 + e_i$$

Model for cross-sectional associations, M_{smok} adjusted:

$$\frac{(LF_2 - LF_1)}{(T2 - T1)_{in\ year}} = \beta_0 + \beta_1 residu_{DNAm} + \beta_2 LF_1 + \beta_3 age + \beta_4 age^2 + \beta_5 height + \beta_6 \big(height - mean(height)\big)^2 + \beta_7 sex + \beta_{8_0} area_1 + \cdots + \beta_{8_k} area_k + \beta_9 bmi + \beta_{10_1} educ_1 + \beta_{10_2} educ_2 + \beta_{11}\ Bcell + \beta_{12} CD4T + \beta_{13} CD8T + \beta_{14} Eos + \beta_{15} Mono + \beta_{16} Neu + \beta_{17} NK + \beta_{18} sex * age + \beta_{19} sex * age^2 + \beta_{20} sex * height + \beta_{21} sex * \big(height - mean(height)\big)^2 + \beta_{21_1} Smok\_status_1 + \beta_{21_2} Smok\_status_2 + \beta_{22} packyrs + e_i$$

<u>Linear mixed regression model, mathematical formula</u>

Repeated cross-sectional analysis corresponds to linear mixed model with random intercept γ_{0i} :

$$\begin{cases} y_{ij} = \beta_0 + \sum_{k=1}^{p} \beta_k x_{kij} + \gamma_{0i} + e_{ij} \\ i = 1, \ldots, n\ j = 1,2 \\ \gamma_{0i} \sim N(0, \sigma^2_{\gamma_0}) \\ e_{ij} \sim N(0, \sigma^2_e) \\ (\gamma_{0i}, \ldots, \gamma_{0n}) indd (\gamma_{0i}, \ldots, \gamma_{0n}) \end{cases}$$

Model for repeat cross-sectional associations, M_{base} adjusted:

$$LFj = \beta_0 + \gamma_{0j} + \beta_1 residu_{jDNAm} + \beta_2 age_j + \beta_3 age^2{}_j + \beta_4 height_j + \beta_5 \big(height_j - mean(height)_j\big)^2 + \beta_6 sex + \beta_{7_0} area_{1j} + \cdots + \beta_{7_k} area_{kj} + \beta_8 bmi_j + \beta_{9_1} educ_{1j} + \beta_{9_2} educ_{2j} + \beta_{10}\ Bcell_j + \beta_{11} CD4T_j + \beta_{12} CD8T_j + \beta_{13} Eos_j + \beta_{14} Mono_j + \beta_{15} Neu_j + \beta_{16} NK_j + \beta_{17} sex * age_j + \beta_{18} sex * age^2{}_j + \beta_{19} sex * height_j + \beta_{20} sex * \big(height_j - mean(height)_j\big)^2 + e_{ij} \qquad j = 1,2$$

Model for repeat cross-sectional associations, M_{smok} adjusted:

$$LFj = \beta_0 + \gamma_{0j} + \beta_1 residu_{jDNAm} + \beta_2 age_j + \beta_3 age^2{}_j + \beta_4 height_j + \beta_5 \big(height_j - mean(height)_j\big)^2 + \beta_6 sex + \beta_{7_0} area_{1j} + \cdots + \beta_{7_k} area_{kj} + \beta_8 bmi_j + \beta_{9_1} educ_{1j} + \beta_{9_2} educ_{2j} + \beta_{10}\ Bcell_j + \beta_{11} CD4T_j + \beta_{12} CD8T_j + \beta_{13} Eos_j + \beta_{14} Mono_j + \beta_{15} Neu_j + \beta_{16} NK_j + \beta_{17} sex * age_j + \beta_{18} sex * age^2{}_j + \beta_{19} sex * height_j + \beta_{20} sex * \big(height_j - mean(height)_j\big)^2 + \beta_{21_1} Smok\_status_{1j} + \beta_{21_2} Smok\_status_{2j} + \beta_{22} packyrs_{ij} + e_{ij} \qquad j = 1,2$$

3.2. *Generation of smoking scores*

For the discovery cohorts, two types of smoking indices (SI) were constructed according to an algorithm based on the deviation from the reference group mean of the measured DNAme across a subset of CpGs in a given participant.[44] To build smoking indices specific to each lung function outcome in the discovery cohorts, we used the technical bias-adjusted residuals of the measured DNAme. Never smokers were defined as the reference group to derive the mean and standard deviation (μc and σc) used in the index equation. For each CpG of the selected subset, the mean DNAme, μ*c*, and its standard deviation, σ*c*, across the group of never smokers (reference group) was computed. In detail, the smoking index was defined across a subset of a number of CpGs (N) in a given participant (SI(*s*)) using the DNAme measured in the biological sample (s) by summing the difference in DNAme level at a given CpG (β*cs*) from the mean reference DNA methylation (μc) divided by the standard deviation in the reference group (σc) and by taking the direction hyper- or hypomethylation the smoking-associated CpG into account (*wc,* hypermethylation =+1 and hypomethylation=-1). The direction of the effect of smoking on DNAme was derived from Joehanes et al[45].

$$\mathrm{SI}(s) = \frac{1}{n}\sum_{c}^{n} w_c \frac{\beta_{cs} - \mu_c}{\sigma_c},$$

Using this algorithm, an SI can be constructed for all participants irrespective of their smoking status. Two different subsets of CpGs were selected to test their combined effect in a SI score, the Mediation-SI (i) and the lung-function-gene-SI (ii)

i) Mediation- SI: In a recent report 10 CpGs had been identified to be associated with smoking applying an independent EWAS in the LifeLines cohort study and confirmed by mediation analysis to be mediators of smoking on lung function [46]. We used these 10 CpGs for constructing a mediation SI. The mediator CpGs were marked in the single CpG association result tables

ii) Lung-function-gene-SI: A smoking index based on the previously identified Bonferroni-corrected smoking associated (p<0.05) CpGs located in 18 GWAS-identified lung function candidate genes, namely *ADAM19, ARMC2, C10orf11, CDC123, CFDP1, FLJ20184, HDAC4, HTR4, LRP1, MECOM, MFAP2, PPT2, RARB, RHOBTB3, TGFB2, TLE3, TNS1, ZNF323/ZKSCAN3* [45]. One CpG per locus was selected, choosing the CpG most consistently associated with smoking as reported previously [45] to contribute to the candidate smoking index score (see table S24).

3.3. Annotation of genomic loci of replicated CpGs

Annotation to genomic location was achieved by permutations testing. To determine whether enrichment occurred more often than expected by chance we drew 10000 random sets with matched SD structure as the lung funciton associated loci from the Illumina 450K arrays probes. For each set, we recorded the overlap to the genomic feature under test, creating a distribution that reflected the overlap of a random permuted set of 450K probes with the same standard squared deviation (SD) structure to the genomic feature. We obtained enrichment p-values

empirically from distribution of overlaps generated by the permuted sets and the observed overlap in the lung function associated loci, and performed a Fisher exact test by cross tabulation of the mean overlaps from the permuted sets versus the observed overlap in lung function associated loci. The SD structure of the 450k probes was recorded in NFBC1966. Subsequently, the 450K probes were divided into 10 SD bins. The 10000 permuted sets were created containing the same number of probes per SD bin as the credible set of loci.

We assessed the overlap of our lung function specific marker to histone modification H3K4, H3K27me3 and the chromatin state model reported by Roadmap.[47] DNAse hypersensitivity sites as well as transcription factor binding sites data were retrieved from ENCODE project.[48] Chromosomal contact points and domains (HiC) were downloaded from GEO and concordant experiments were undertaken as previously described.[49] To assess the overlap to SNP signatures we used the NHGRI-EBI GWAS catalogue (downloaded June 2016). SNPs per trait were pruned across studies using a 1MB window. We restricted this analysis to traits with 50 or more independent variants. To define enrichment in regions relative to the transcription start site of a gene and CpG island we used Illumnina's 450K manifest file.

3.4. Pathway analyses of replicated CpGs

Genes from the 57 confirmed sentinel CpGs, replicated in independent cohorts, were selected for further analysis. The annotation has been used provided by Illumina and any missing genes were populated using Snipper v1.2 (https://csg.sph.umich.edu/boehnke/snipper/). The gene list was then used for enrichment in KEGG (Kyoto Encyclopedia of Genes and Genomes)[50] and GO (Gene Ontology) databases[51] using topGO and topKEGG function in the missMethyl package.[52] Additionally, the Ingenuity Pathway Analysis system - IPA® (QIAGEN Inc., https://www.qiagenbioinformatics.com/products/ingenuity-pathway-analysis) was used to identify global canonical pathways.[53]

3.5. Smoking enrichment analysis

We performed an enrichment analysis to test if CpGs previously identified as being associated with smoking were statistically overrepresented among the top lung function associated CpG markers of the discovery meta-analyses EWAS. We defined a CpG marker to be a smoking CpG if it was part of a previously reported set of CpG markers (n = 18,760) associated with smoking behavior at a FDR-corrected P-value<0.05.[45] The test statistics (t-values) were retrieved from each EWAS result and tested for the enrichment of the smoking-related CpG sites. The Weighted Kolmogorov Smirnov (WKS) test was used to assess if the test statistics on a certain set of loci differed from those in random loci of the same size.[54] The WKS is a better alternative to the commonly used gene set enrichment analysis[55] for examining an enrichment of custom curated CpG marker sets, as this method may be less biased towards identifying enrichment of large genes with increased number of probes represented on the arrays.

3.6. Replication of mediation analysis in SAPALDIA

We conducted mediation analysis for each of the 10 CpGs used to compute the Mediation-SI, using R package "mediation". SAPALDIA samples at time point 2 were analyzed (n = 962). Mediation model was fitted by linear regression of technical variable adjusted residuals of the methylation level on smoking status (ever vs never-smoker)

after adjustment for age, sex, body mass index, education, study center, and cell composition. Outcome model was fitted by linear regression of lung function on the technical variable adjusted residuals of the methylation level after adjustment for the same covariates of base model. The total effect of smoking, the effect of changing smoking status from never to ever-smoker, would be then decomposed into two causal mediation effect and direct effect. The causal mediation effect denotes the effect of changing technical variable adjusted residuals by one unit, conditional on the smoking status. The population average of the causal mediation effect and the direct effect, adjusted for the base model covariates, was estimated by quasi-Bayesian Monte Carlo simulations.

3.7. Two-sample Mendelian randomization study of the replicated CpGs

Using the publicly available database, Accessible Resource for integrative Epigenomic Studies (ARIES), we identified methylation quantitative trait loci (mQTL) in peripheral blood of participants of the ALSPAC study, at four different life stages (birth, childhood, adolescence, middle age) for 13 of the 57 replicated CpGs.[56] To look-up in a two-sample Mendelian randomization (MR) approach whether the identified mQTL-associated single nucleotide polymorphisms (SNPs) were associated with FEV_1 or FVC, we used the publicly available curated genome-wide association study database (MR-Base).[57] The alleles were harmonized to ensure that SNP-exposure effect correspond to the same allele for SNP-outcome effect at the adult time point (termed middle age in the ARIES data). MR effect estimates were calculated using Wald ratio and the resulting effect estimate represents the change in outcome per unit increase in the exposure. The SNP-outcome effect sizes were selected based on outcome FEV_1 and FVC. All analyses were done in R/3.4.4 environment using “Two Sample MR” package.

4. Results: Sentinel CpG marker selection for replication

4.1. Table S1: List of sentinel CpGs for replication, all participants

Table S1: Selection of sentinel CpGs for replication for FEV_1, FEV_1/FVC or FVC in all participants of the discovery cohorts, EWAS meta-analyses, basic covariate-adjusted (M_{base}) and additionally smoking adjusted (M_{smok}) associations are presented in brackets. CpG markers with P-value <5x10-7 were selected.

								FEV1				FEV1/FVC				FVC			
**CpG ID**	**chr**	**position**	***Locus***	**sign**	**best rank (rank Msmok)**	**Model, base covairates**	**Msmok (Model, smoking adjusted)**	**EWAS Cross-sectional Time point 1**	**EWAS Cross-sectional Time point 2**	**EWAS Repeat cross-sectional analyses (Both time points)**	**Prediction EWAS on change in lung function**	**EWAS Cross-sectional Time point 1**	**EWAS Cross-sectional Time point 2**	**EWAS Repeat cross-sectional analyses (Both time points)**	**Prediction EWAS on change in lung function**	**EWAS Cross-sectional Time point 1**	**EWAS Cross-sectional Time point 2**	**EWAS Repeat cross-sectional analyses (Both time points)**	**Prediction EWAS on change in lung function**
cg04885881	1	11123118		+++	14	x						3.51E-07	6.42E-09	8.51E-09					
cg21393163	1	12217629	*TNFRSF 1B*	+++	16	x					3.01E-07								
cg27537125	1	25349681	*RUNX3*	+++	7	x					2.04E-08								
cg21140898	1	51442318		+++	17	x							3.04E-08						
cg09935388	1	92947588	*GFI1*	+++	19	x								3.60E-07					
cg19266329	1	145456128		+++	1 (1)	x	x		5.44E-11 (1.83E-07)								1.03E-08 (2.29E-07)		
cg11231349	1	162050656	*NOS1AP*	??+	47	x			1.92E-07				2.99E-07						
cg03547355	1	227003060		+++	40	x			1.22E-07										
cg20853880	2	10184444	*KLF11*	+++	20	x							1.23E-07						
cg22475025	2	43864340	*PLEKHH2*	++-	2		x				3.47E-07								
cg22040631	2	129153820		??+	30	x			4.85E-08										
cg18826637	2	145116633		+++	11	x			8.92E-10										
cg05135521	2	161188335	*RBMS1*	+++	37	x			8.53E-08										
cg02514318	2	197201183	*HECW2*	+++	63	x			4.78E-07										
cg27241845	2	233250370	*ALPPL2*	+++	20	x			7.77E-09		2.85E-08								
cg17087741	2	233283010	*ALPPL2*	+++	6	x							5.13E-12						
cg03329539	2	233283329	*ALPPL2*	+++	9	x			2.36E-07				1.77E-10	7.41E-08					
cg05951221	2	233284402	*ALPPL2*	?++	11	x					5.81E-08								
cg21566642	2	233284661	*ALPPL2*	+++	3	x			1.77E-11		3.67E-13	2.77E-07	3.47E-17	1.39E-11	2.93E-08				
cg01940273	2	233284934	*ALPPL2*	+++	2	x			5.77E-11		1.27E-12		8.11E-18	3.10E-11	1.05E-07				

cg01377124	2	237172609	*ASB18*	--+	1 (1)	x	x								1.01E-09 (2.70E-09)				
cg11610350	3	64253705		+++	1		x							1.63E-07					
cg16990174	3	72496875	*RYBP*	+++	16	x								3.09E-07					
cg19859270	3	98251294	*GPR15*	+++	29	x			3.68E-08				8.07E-08						
cg22870429	3	114027859	*TIGIT*	+++	23	x			1.52E-08										
cg13457961	3	186501085	*SNORD2*	---	3		x				4.71E-07								
cg01598596	3	187464648	*BCL6*	+++	58	x			3.48E-07										
cg14855367	3	191048308	*UTS2D*	+++	28	x							3.12E-07						
cg00741986	4	2748332	*TNIP2*	+++	60	x			4.06E-07										
cg08763102	4	3079751	*HTT*	+++	11	x								1.19E-07					
cg24086068	4	77356008	*SHROOM3*	+-+	3	x			7.80E-09								1.59E-07		
cg01899089	5	369969	*AHRR*	-++	15	x								3.08E-07					
cg05575921	5	373378	*AHRR*	+++	1	x			9.33E-14	1.25E-07	2.01E-15	5.12E-10	3.96E-21	3.59E-16	1.59E-08				3.97E-07
cg26703534	5	377358	*AHRR*	+++	15	x					5.87E-08		1.33E-08						
cg25648203	5	395444	*AHRR*	+++	8	x			3.64E-08		7.15E-09		5.02E-11						
cg21161138	5	399360	*AHRR*	+++	5	x				2.01E-08	1.43E-08	2.73E-07	6.36E-14	1.62E-10					
cg05673882	5	74862702	*POLK*	+++	10	x			3.31E-10										
cg23205886	5	138611766	*SNHG4*	+++	4	x			2.07E-09								2.55E-07		
cg05487895	5	139080952		---	2		x										2.79E-07		
cg07222133	5	179499488	*RNF130*	?-+	6 (3)	x	x								2.00E-07 (3.95E-07)				
cg01882991	6	6677756		+++	22	x			1.22E-08										
cg15930777	6	12343201		+++	13	x		4.88E-07											
cg14753356	6	30720108		+++	4	x						8.01E-08	2.04E-07						
cg24859433	6	30720203		+++	12	x						3.80E-07							
cg15342087	6	30720209	*FLOT1*	+++	8	x			6.23E-11		2.04E-07		4.18E-09	1.62E-07					7.53E-08
cg05593667	6	35490744		+?+	51	x			2.26E-07										
cg03149958	6	36326677	*ETV7*	+++	1 (3)	x	x		5.44E-12 (9.31E-09)		1.39E-07						5.19E-08 (3.78E-07)		2.77E-10 (1.92E-08)
cg03957124	6	37016869		+++	33	x			6.01E-08										
cg21282907	6	74289980		+++	10	x			4.60E-09				1.65E-09						
cg12761472	6	119031922		+++	32	x			5.28E-08										
cg00073460	6	149806502	*ZC3H12D*	+++	27	x			3.60E-08										

cg06762457	6	149806635	*ZC3H12D*	+++	25	x			2.59E-08		3.58E-07								
cg03856024	6	158039220	*ZDHHC14*	??+	22 (1)	x	x						1.52E-07 (3.89E-07)						
cg08549335	7	30387954	*ZNRF2*	+++	16	x							3.16E-09				4.74E-07		
cg26521259	7	43435880	*HECW1*	?++	7(1)	x	x					2.10E-07 (1.17E-07)							
cg10691866	7	65817282	*TPST1*	+++	59	x			3.89E-07										
cg25949550	7	145814306	*CNTNAP2*	+++	24	x							1.95E-07						
cg01651915	8	55795551	*XKR4*	+++	4	x			1.56E-07										3.86E-07
cg13353574	8	61326925		+++	18	x								3.51E-07					
cg20090859	8	80590435		?--	2 (2)	x	x									2.08E-07 (2.07E-07)			
ch.8.91748119F	8	91678943		+++	10 (1)		x				3.47E-08 (4.29E-08)								
cg07292140	8	94210841		??+	27	x							3.06E-07						
cg19589396	8	103937374		+++	62	x			4.51E-07										
cg13064897	8	135747178		+++	6 (2)	x	x					2.00E-07 (3.57E-07)							
cg12075928	8	141801307	*PTK2*	+++	46	x			1.82E-07										
cg05329578	9	2241688		+++	17	x								3.39E-07					
cg02716826	9	33447032	*SUGT1P1*	+++	55	x			2.53E-07										
cg13891189	9	116164001	*ALAD*	+++	8	x										2.45E-07			
cg13657200	9	117266029	*DFNB31*	+++	49	x			2.07E-07										
cg14366110	9	133779382	*FIBCD1*	+++	5(2)	x	x								1.24E-07 (1.66E-07)				
cg04813697	10	22920025	*PIP4K2A*	+++	43	x			1.70E-07										
cg25953130	10	63753550	*ARID5B*	+++	5	x			2.74E-09								2.79E-07		
cg00210249	10	71135679	*HK1*	---	18	x					3.68E-07								
cg03450842	10	80834947	*ZMIZ1*	+++	23	x							1.80E-07						
cg18879828	11	46942432		+++	64	x			4.99E-07										
cg21611682	11	68138269	*LRP5*	+++	16	x							1.63E-08	1.78E-07					
cg11660018	11	86510915	*PRSS23*	+++	6	x			3.21E-08			1.29E-08	5.14E-09	7.12E-11					
cg23771366	11	86510998	*PRSS23*	+++	3	x				2.07E-07			2.69E-09	1.37E-11					
cg21990700	12	7260776	*C1RL*	+++	12	x			1.18E-09										
cg07986378	12	11898284	*ETV6*	+++	8	x					2.11E-08								
cg06826457	12	12867669		+++	53	x			2.46E-07										

cg26165146	12	27484656	*ARNTL2*	+++	5	x						1.93E-07							
cg20059012	12	53613154	*RARG*	+++	45	x			1.77E-07										
cg02583484	12	54677008	*HNRNPA 1*	+++	34	x			6.76E-08										
cg00666749	13	21637118	*LATS2*	+++	41	x			1.50E-07										
cg16708465	13	95933097	*ABCC4*	+++	38	x			9.83E-08										
cg12033216	14	59130157		+++	57	x			3.24E-07										
cg13976502	14	74227875	*C14orf43*	+++	35	x			7.06E-08										
cg16288101	14	88621538		+++	19	x			5.80E-09										
cg25292882	15	39431467		+++	31	x			5.07E-08										
cg22952142	15	68549178		+++	20	x								4.03E-07					
cg00310412	15	74724918	*SEMA7A*	+++	14	x			2.08E-09				3.10E-08	1.27E-07					
cg06505619	16	698072	*WDR90*	+++	54	x			2.49E-07										
cg05557932	16	3929351	*CREBBP*	+++	36	x			7.10E-08										
cg16391678	16	30485597	*ITGAL*	+++	21	x							3.31E-07						
cg01243823	16	50732212	*NOD2*	+++	48	x			2.05E-07										
cg27367615	16	86229910		+++	2		x							3.31E-07					
cg01747591	16	89703612	*DPEP1*	+++	6	x											4.20E-07		
cg09395195	17	29645782	*EVI2A*	+++	50	x			2.24E-07										
cg19572487	17	38476024	*RARA*	+++	7	x			6.04E-11					2.49E-09			4.89E-11		
cg20458044	17	57904327	*TMEM49*	+++	61	x			4.38E-07										
cg18181703	17	76354621	*SOCS3*	+++	39	x			1.04E-07										
cg03636183	19	17000585	*F2RL3*	+++	4	x			5.38E-13	1.75E-07	5.64E-11	7.09E-08	1.47E-15	5.25E-13					
cg07709627	19	30156658	*PLEKHF1*	---	1 (1)	x	x											2.23E-08 (2.38E-08)	
cg26768816	19	30302793	*CCNE1*	--+	1 (1)	x	x									3.46E-08 (3.84E-08)			
cg07626482	19	47289503	*SLC1A5*	+++	18	x			5.39E-09										
cg03707168	19	49379127	*PPP1R15 A*	+++	29	x							4.38E-07						
cg16201146	20	19191526		+++	21	x								4.97E-07					
cg12303084	20	45985741	*ZMYND8*	+++	14	x			2.72E-07		1.66E-07								
cg23110422	21	40182073	*ETS2*	+++	44	x			1.74E-07										
cg01127300	22	38614796		+++	9	x			1.85E-10										

4.2. Table S2: List of sentinel CpGs for replication, never smokers

<u>Table S2:</u> Selection of sentinel CpGs for replication for FEV_1, FEV_1/FVC or FVC in never smokers of the discovery cohorts. CpG markers with P-value <5x10-7 were selected.

						FEV1				FEV1/FVC				FVC			
**CpG ID**	**chr**	**position**	***Locus***	**sign**	**rank**	**EWAS Cross-sectional Time point 1**	**EWAS Cross-sectional Time point 2**	**EWAS Repeat cross-sectional analyses (Both time points)**	**Prediction EWAS on change in lung function**	**EWAS Cross-sectional Time point 1**	**EWAS Cross-sectional Time point 2**	**EWAS Repeat cross-sectional analyses (Both time points)**	**Prediction EWAS on change in lung function**	**EWAS Cross-sectional Time point 1**	**EWAS Cross-sectional Time point 2**	**EWAS Repeat cross-sectional analyses (Both time points)**	**Prediction EWAS on change in lung function**
cg25758394	1	3623859	*TP73*	??+	1		1.727E-09				2.687E-08				1.435E-07		
cg22508172	1	24069723	*TCEB3*	---	2											1.166E-07	
cg12752420	1	43751363	*C1orf210*	+++	4										1.961E-07		
cg10212705	1	154297848	*ATP8B2*	---	3											1.961E-07	
cg18938392	1	157248950		+++	2							2.874E-08					
cg05785298	1	204654622	*LRRN2*	-+-	6						9.099E-08						
cg11216682	2	131113867	*PTPN18*	+-+	2								9.054E-08				
cg15981995	3	169487311	*ARPM1*	??-	5						5.042E-08	4.286E-07					
cg18664508	3	169487465	*ARPM1*	---	2		1.917E-08				2.929E-08						
cg04030659	6	22570704	*HDGFL1*	---	4											3.66E-07	
cg27235034	6	29976917	*HLA-J*	-++	3					3.535E-07							
cg12647932	6	70576740	*COL19A1*	---	1					3.669E-08							
cg17838734	6	83073924	*TPBG*	---	1			7.973E-09									
cg20098854	8	898407		---	1												1.316E-07
cg25633955	8	1616622	*DLGAP2*	---	3										3.865E-07		
cg19931644	8	12623485		+++	3				2.757E-07								
cg13562246	8	33368277	*C8orf41*	+++	8						1.928E-07						
cg04460372	9	130661175	*ST6GALNAC6*	+++	2	3.544E-07											
cg14366110	9	133779382	*FIBCD1*	---	1							1.718E-10	4.237E-09				
cg05831672	10	103543172	*NPM3*	---	1											3.929E-08	
cg04774364	10	106100810		+++	2												1.618E-07
cg00911551	12	8234647	*NECAP1*	---	1	2.431E-07											
cg25668058	12	115183175		---	2									2.097E-07			
cg07922154	14	68087339	*ARG2*	+++	2			9.626E-08									
cg04975143	14	106438118	*ADAM6*	---	1									1.784E-07			
cg05622686	16	58501611	*NDRG4*	+++	2										2.977E-07		
cg08447479	16	75589467	*TMEM231*	+++	1				3.28E-07								
cg20278790	20	57583474	*CTSZ*	-++	3		2.507E-07			2.81E-07	1.618E-07						2.722E-07

5. Results: Cross-sectional associations with each lung function measure, without smoking adjustment

Cross-sectional association with each lung function measure at time point 1 and at time point 2, separately: discovery, replication and combined EWAS meta-analyses in all participants are presented in table S3 for FEV_1/FVC; table S4 for FEV_1 and table S5 for FVC.

The top association signals observed in the repeat cross-sectional associations with each lung function measure is presented in table S6.

5.1. Table S3 (FEV_1/FVC), Table S4 (FEV_1) and Table S5 (FVC)

Table S3: Discovery and replication meta-analyses of cross-sectional association with **FEV_1/FVC** in all participants*, base model covariate adjusted EWAS (M_{base}†) separately at two time points. Combined meta-analyses of cross-sectional associations obtained using data from time point T2 of ECRHS, NFBC1966, SAPALDIA, LBC1936 and from time point T1 of KORA, LifeLines and NSPHS (excluding FTC replication cohort due to the younger mean age).*See table in See table in EXCEL file: Additional_Tables_Imbodenetal.xlsx*

Table S4: Discovery and replication meta-analyses of cross-sectional association with **FEV_1** in all participants*, base model covariate adjusted EWAS (M_{base}†) separately at two time points. Combined meta-analyses of cross-sectional associations obtained using data from time point T2 of ECRHS, NFBC1966, SAPALDIA, LBC1936 and from time point T1 of KORA, LifeLines and NSPHS (excluding FTC replication cohort due to the younger mean age).*See table in EXCEL file: See table in EXCEL file: Additional_Tables_Imbodenetal.xlsx*

Table S5: Discovery and replication meta-analyses of cross-sectional association with **FVC** in all participants*, base model covariate adjusted EWAS (M_{base}†) separately at two time points. Combined meta-analyses of cross-sectional associations obtained using data from time point T2 of ECRHS, NFBC1966, SAPALDIA, LBC1936 and from time point T1 of KORA, LifeLines and NSPHS (excluding FTC replication cohort due to the younger mean age).
See table in EXCEL file: Additional_Tables_Imbodenetal.xlsx

Footnote to table S3, table S4 and table S5:
* Presentation of CpG markers showing meta-analysis P-value < $5x10^{-7}$ at combined meta-analysis. CpGs consistent in direction of the cross-sectional associations at both time points were for FEV_1/FVC: cg05575921, cg21161138, cg26703534 and cg25648203 (*AHRR*), cg03636183 (*F2RL3*), cg21566642, cg01940273 and cg03329539 (in vicinity of *ALPPL2)*, cg23771366 and cg11660018 (*PRSS23*), cg15342087 (*IER3*), cg19572487 (*RARA*), cg24859433, and cg14753356, cg15342087 (*FLOTL1*) and cg04885881 (*SRM*); for FEV_1: cg03636183 (*F2RL3*), cg11660018 (*PRSS23*), cg19572487 (*RARA*), cg23771366 (*PRSS23*), cg03149958 (*ETV7*), cg18181703 (*SOCS3*), cg05673882 (*POLK*), cg01127300, cg04813697 (*PIP4K2A*), cg21990700 (C1RL), cg06826457, cg25953130 (*ARID5B*), cg01882991 (intergenic), cg16288101 (intergenic), cg12761472 (intergenic), cg11231349 (*NOS1AP*); and none for FVC.
† Base model (M_{base}) EWAS were covariate adjusted for age, age squared, height, squared deviation from the mean of height, sex and interaction terms of age, age squared, height and squared deviation of height with sex, education (low, medium, high), body mass index, spirometer type, study center as well as cell composition.
‡ Smoking CpGs defined on the reported FDR corrected P-value <0.05 for association reported with smoking status and direction of effects.[45]
¶ For combined meta-analysis: FTC was excluded from this meta-analysis, given the smaller sample size and lower mean age (30.4 yrs) compared to the other adult cohorts (ECRHS (mean age: 54.5 yrs), NFBC1966 (46.3 yrs), SAPALDIA (58.8 yrs), LBC1936 (76.3 yrs) and the single available time point for KORA (60.1 yrs), LifeLines (46.7 yrs) and NSPHS (55.1 yrs)).

5.2. Figure S2: Quantile-Quantile plot of cross-sectional EWAS on FEV_1 and FVC

Figure S2: Quantile-Quantile plots of cross-sectional covariate-adjusted EWAS (M_{base}*) on FEV1 and on FVC at first and second time point, all participants. Increase in numbers of signals with aging. Meta-analyses were performed without genomic control. For FEV_1, we identified 34 CpGs at time point 2 compared to none at time point 1 to be statistically significant (inflation factor λ for time point 1 (λ = 1.14) and for time point 2 (λ = 1.14)). For FVC, we identified three CpGs at time point 2 compared to none at time point 1 to be statistically significant (inflation factor λ for time point 1 (λ = 1.09) and for time point 2 (λ = 1.02)).

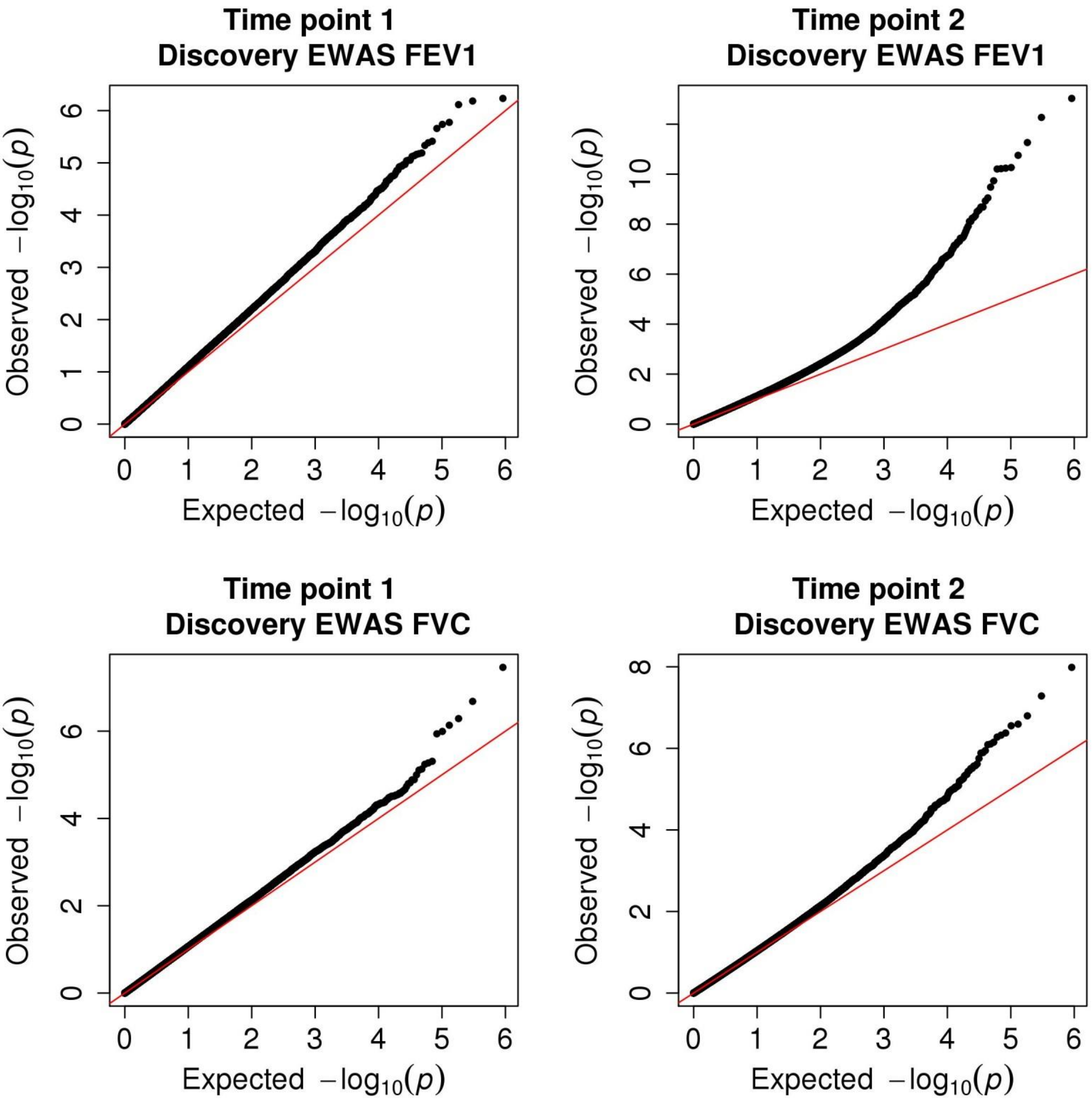

Footnote to Figure S2:

*Base model (M_{base}): EWAS were covariate adjusted for age, age squared, height, squared deviation from the mean of height, sex and interaction terms of age, age squared, height and squared deviation of height with sex, education (low, medium, high), body mass index, spirometer type, study center as well as cell composition.

6. Results: EWAS$_{repeat}$ - Repeat cross-sectional EWAS in the same study participants – combining the data from both time points

The discovery EWAS$_{repeat}$ was undertaken using mixed linear regression with a random intercept on the study participant to assess the data from both time points in the same model. The EWAS$_{repeat}$ results are presented only in the online supplement (Figure Box1 and Table S6, this Appendix). For FEV_1, we found seven CpGs associated either in discovery EWAS meta-analysis or in the combined meta-analysis of four cohort studies to be associated with a $P<5x10^{-7}$. Three CpGs did formally replicate in the LCB36 replication cohort (P<0.0071, Bonferroni correction for 7 tests). All three replicated differentially methylated markers had previously been identified as smoking-related CpGs: cg05575921, in the *AHRR* gene replicating at $P_{replication}$= 0.0033 ($P_{combined}$=$1.86x10^{-9}$), cg21566642 in the *ALPPL2* gene (2q37.1) at $P_{replication}$= 0.0033 ($P_{combined}$=$1.86x10^{-9}$) and cg04813697 in the *PIP4K2A* gene at $P_{replication}$= 0.0006 ($P_{combined}$=$4.61x10^{-7}$). Results (not shown) see Box3 Figure "Manhattan and Q-Q plots of EWAS$_{repeat}$"). For FEV_1/FVC, there were 23 CpG markers found to be associated at P-value < 5x 10-7 and six replicated formally at a P<0.0021 (Bonferroni correction for 23 tests). Again all replicated CpGs were known smoking-related CpGs. In addition to the five prominent differentially methylated markers (cg21566642 and cg01940273 in the *ALPPL2* gene (2q37.1), cg05951221 in the *AHRR* gene, and cg03636183 in the gene *F2RL3),* we identified cg09935388 in gene *GFI1* replicating at $P_{replication}$=0.0001 ($P_{combined}$=$2.54x10^{-9}$).

For FVC, only one CpG marker (cg07709627 in gene *PLEKHF1*) reached the genome-wide selection threshold for replication in the repeated cross-sectional analyses, but it did not replicate. This method performed poorer as fewer statistically significant CpGs were identified compared to the cross-sectional EWAS using data from the second time point. Comparing the results of the repeated cross-sectional EWAS with the results of two cross-sectional EWAS, we noted for some CpGs consistent associations for both approaches. For other CpGs it seemed that fixed effect linear approach at two time points revealed more easily consistent and statistically significant associations compared to the mixed linear models likely due to increased sample size of the replication cohort for the cross-sectional analyses.

Box1 Figure: EWAS$_{repeat}$ Manhattan and QQ plots, in all participants

__Figure:__ Manhattan and Quantile-Quantile plots of repeat cross-sectional EWAS (Mbase*) combining data from both time points, all participants A) on FEV_1 (λ=1.23); B) FEV_1/FVC (λ=1.13); and C) FVC (λ=1.13).

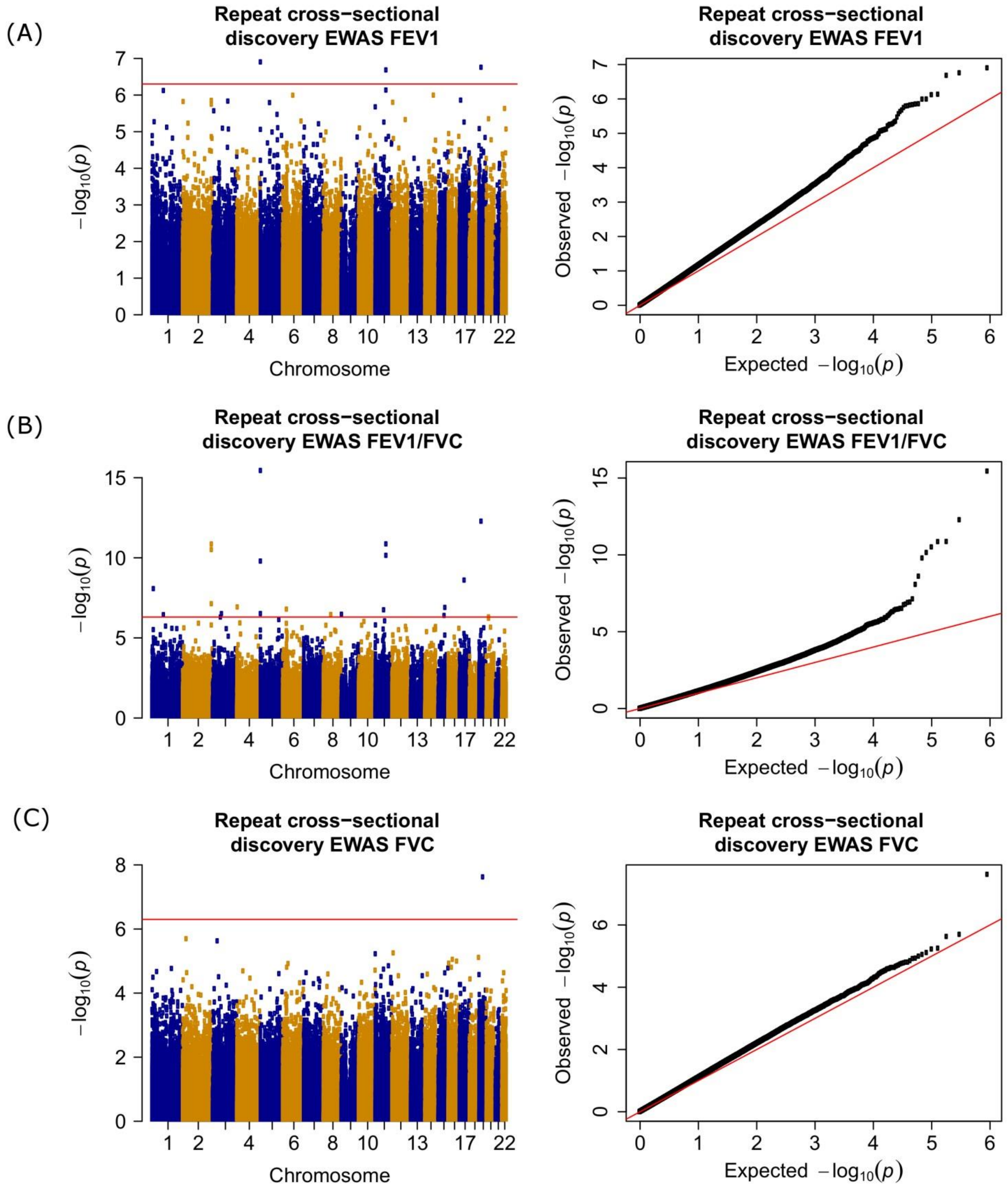

Footnote:

*Base model (M_{base}): EWAS were covariate adjusted for age, age squared, height, squared deviation from the mean of height, sex and interaction terms of age, age squared, height and squared deviation of height with sex, education (low, medium, high), body mass index, spirometer type, study center as well as cell composition.

6.1. Table S6: Repeat cross-sectional associations with lung function in all participants ($EWAS_{repeat}$, M_{base})

Table S6: Discovery, replication and combined EWAS meta-analyses of repeat cross-sectional association with lung function in all participants*, base model covariate adjusted EWAS (M_{base}†). Covariate-adjusted mixed linear regressions with a random intercept on the subject were undertaken using data from both time points (repeat cross-sectional analysis ($EWAS_{repeat}$)).

					**Discovery (ECRHS/NFBC/SAPALDIA)**				**Replication (LCB1936)**				**Combined meta-analysis (ECRHS/NFBC/SAPALDIA/LBC1936)**			
**CpG ID**	**chr**	**position**	***Locus***	**smoking CpGs¶**	**beta (SE)**	**P-value meta-analysis**	**direction of effect**	**P-value study hetero-geneity**	**beta (SE)**	**P-value of replication ‡**	**direction of effect**	**Repli-cated ‡**	**beta (SE)**	**P-value meta-analysis**	**direction of effect (four studies)**	**P-value study hetero-geneity**
**FEV1**																
cg21566642	2	233284661	*ALPPL2*	yes	0.391(0.081)	1.41E-06	+++	0.220	0.497(0.165)	0.0027	+	no	0.411(0.073)	**1.59E-08**	++++	0.339
cg05575921	5	373378	*AHRR*	yes	0.362(0.068)	**1.25E-07**	+++	0.408	0.478(0.163)	0.0033	+	no	0.379(0.063)	**1.86E-09**	++++	0.527
cg04813697	10	22920025	*PIP4K2A*	yes	0.459(0.124)	0.0002	+++	0.460	0.502(0.146)	**0.0006**	+	**yes**	0.477(0.095)	**4.61E-07**	++++	0.659
cg23771366	11	86510998	*PRSS23*	yes	0.743(0.143)	**2.07E-07**	+++	0.362	0.33(0.223)	0.1402	+	no	0.623(0.121)	**2.36E-07**	++++	0.216
cg16288101	14	88621538		yes	0.53(0.108)	1.01E-06	+++	0.101	0.329(0.173)	0.0575	+	no	0.473(0.092)	**2.56E-07**	++++	0.136
cg19572487	17	38476024	*RARA*	yes	0.525(0.116)	5.50E-06	+++	0.281	0.457(0.174)	0.0084	+	no	0.504(0.096)	**1.58E-07**	++++	0.450
cg03636183	19	17000585	*F2RL3*	yes	0.543(0.104)	**1.75E-07**	+++	0.987	0.484(0.192)	0.0117	+	no	0.53(0.091)	**6.88E-09**	++++	0.992
**FEV1/FVC**					**beta (SE)**	**P-value meta-analysis**	**direction of effect**	**P-value study hetero-geneity**	**beta (SE)**	**P-value of replication ‡**	**direction of effect**	**Repli-cated ‡**	**beta (SE)**	**P-value meta-analysis**	**direction of effect**	**P-value study hetero-geneity**
cg04885881	1	11123118		yes	0.116(0.02)	**8.51E-09**	+++	0.408	0.072(0.054)	0.1792	+	no	0.111(0.019)	**4.55E-09**	++++	0.497
cg09935388	1	92947588	*GFI1*	yes	0.063(0.012)	**3.60E-07**	+++	0.463	0.165(0.043)	**0.0001**	+	**yes**	0.07(0.012)	**2.54E-09**	++++	0.077
cg11231349	1	162050656	*NOS1AP*	yes	0.128(0.027)	2.56E-06	??+	1.000	0.085(0.042)	0.0447	+	no	0.116(0.023)	**4.58E-07**	??++	0.396
cg03329539	2	233283329	*ALPPL2*	yes	0.095(0.018)	**7.41E-08**	+++	0.182	0.116(0.067)	0.0816	+	no	0.096(0.017)	**1.63E-08**	++++	0.320
cg21566642	2	233284661	*ALPPL2*	yes	0.082(0.012)	**1.39E-11**	+++	0.275	0.162(0.04)	**5.58E-05**	+	**yes**	0.088(0.012)	**2.29E-14**	++++	0.101
cg01940273	2	233284934	*ALPPL2*	yes	0.105(0.016)	**3.10E-11**	+++	0.791	0.215(0.055)	**7.95E-05**	+	**yes**	0.113(0.015)	**7.72E-14**	++++	0.233
cg11610350	3	64253705			0.135(0.027)	5.06E-07	+++	0.481	0.101(0.074)	0.1718	+	no	0.131(0.025)	**2.12E-07**	++++	0.648
cg16990174	3	72496875	*RYBP*		0.106(0.021)	**3.09E-07**	+++	0.978	-0.003(0.052)	0.9491	-	no	0.091(0.019)	2.26E-06	+++-	0.275
cg08763102	4	3079751	*HTT*	yes	0.151(0.028)	**1.19E-07**	+++	0.284	-0.048(0.069)	0.4879	-	no	0.122(0.026)	3.73E-06	+++-	0.022
cg01899089	5	369969	*AHRR*	yes	0.125(0.024)	**3.08E-07**	-++	0.006	0.033(0.051)	0.5185	+	no	0.108(0.022)	9.63E-07	-+++	0.005
cg05575921	5	373378	*AHRR*	yes	0.083(0.01)	**3.59E-16**	+++	0.520	0.201(0.038)	**1.48E-07**	+	**yes**	0.091(0.01)	**2.65E-20**	++++	0.017
cg21161138	5	399360	*AHRR*	yes	0.13(0.02)	**1.62E-10**	+++	0.891	0.117(0.053)	0.0286	+	no	0.128(0.019)	**1.44E-11**	++++	0.963
cg24859433	6	30720203		yes	0.142(0.031)	3.58E-06	+++	0.514	0.173(0.079)	0.0283	+	no	0.146(0.029)	**3.15E-07**	++++	0.691
cg15342087	6	30720209	*FLOT1*	yes	0.181(0.035)	**1.62E-07**	+++	0.719	0.221(0.075)	0.0034	+	no	0.188(0.031)	**2.22E-09**	++++	0.827

cg13353574	8	61326925		yes	0.134(0.026)	**3.51E-07**	+++	0.210	-0.026(0.06)	0.6673	-	no	0.109(0.024)	6.94E-06	+++-	0.029
cg05329578	9	2241688		yes	0.074(0.015)	**3.39E-07**	+++	0.937	0.026(0.043)	0.5457	+	no	0.069(0.014)	**4.95E-07**	++++	0.747
cg21611682	11	68138269	*LRP5*	yes	0.136(0.026)	**1.78E-07**	+++	0.943	0.055(0.074)	0.4517	+	no	0.127(0.025)	**2.29E-07**	++++	0.761
cg11660018	11	86510915	*PRSS23*	yes	0.143(0.022)	**7.12E-11**	+++	0.927	0.207(0.061)	**0.0008**	+	**yes**	0.151(0.021)	**3.55E-13**	++++	0.778
cg23771366	11	86510998	*PRSS23*	yes	0.15(0.022)	**1.37E-11**	+++	0.391	0.159(0.058)	0.0058	+	no	0.151(0.021)	**2.89E-13**	++++	0.594
cg22952142	15	68549178			0.071(0.014)	**4.03E-07**	+++	0.813	0.002(0.035)	0.9627	+	no	0.062(0.013)	2.32E-06	++++	0.286
cg00310412	15	74724918	*SEMA7A*	yes	0.145(0.028)	**1.27E-07**	+++	0.348	0.123(0.074)	0.0970	+	no	0.142(0.026)	**3.19E-08**	++++	0.534
cg19572487	17	38476024	*RARA*	yes	0.11(0.018)	**2.49E-09**	+++	0.980	0.11(0.051)	0.0324	+	no	0.11(0.017)	**2.38E-10**	++++	0.998
cg03636183	19	17000585	*F2RL3*	yes	0.113(0.016)	**5.25E-13**	+++	0.752	0.254(0.049)	**2.75E-07**	+	**yes**	0.126(0.015)	**3.40E-17**	++++	0.046
cg16201146	20	19191526		yes	0.133(0.027)	**4.97E-07**	+++	0.786	-0.057(0.06)	0.3436	-	no	0.102(0.024)	2.44E-05	+++-	0.032
**FVC**					**beta (SE)**	**P-value meta-analysis**	**direction of effect**	**P-value study hetero-geneity**	**beta (SE)**	**P-value of replication ‡**	**direction of effect**	**Repli-cated ‡**	**beta (SE)**	**P-value meta-analysis**	**direction of effect**	**P-value study hetero-geneity**
cg07709627	19	30156658	*PLEKHF1*		-4.071 (0.729)	**2.38E-08**	---	0.170	1.559 (1.01)	0.1224	+	no	-2.14 (0.591)	0.0003	---+	2.53E-05

<u>Footnote table S6:</u>

* Presentation of CpG markers showing meta-analysis P-value < $5x10^{-7}$ in the combined meta-analysis.

† Base model (M_{base}) EWAS were covariate adjusted for age, age squared, height, squared deviation from the mean of height, sex and interaction terms of age, age squared, height and squared deviation of height with sex, education (low, medium, high), body mass index, spirometer type, study center as well as cell composition.

‡ Replication was defined for association with FEV_1 if replication P-value<0.00067 (multiple testing correction for 74 tests), with FEV_1/FVC if replication P-value<0.0011 (multiple testing correction for47 tests) and with FVC if replication P-value<0.0031 (multiple testing correction for 16 tests).

¶ Smoking CpGs defined on the reported FDR corrected P-value <0.05 for association reported with smoking status and direction of effects.[45]

7. Results: Effect of smoking adjustment on the cross-sectional EWAS (Ms_{mok}):

After applying smoking adjustment, we identified generally fewer genome-wide significant associations. Interestingly, the top five CpGs were still smoking related methylation markers, but the strength of their association with lung function was greatly diminished e.g. cg23771366 (*PRSS23)* had P=4.61x10^{-8} (M_{smok}) versus P=5.38x10^{-27} (M_{base}), cg05575921 (*AHRR)* had P=2.69x10^{-11} (M_{smok}) versus P=7.22^{-50} (M_{base}), cg03636183 (*F2RL3*) had P=1.02x10^{-8} (M_{smok}) versus P=4.5x10^{-43} (M_{base}). In contrast, the CpG not modified by smoking behavior, cg13064897 on chr8 at 135Mb, did not show an important alteration of the association with FEV_1/FVC after smoking adjustment. The increase in signal strength of the associations observed at time point 2 remained notable even after smoking adjustment (See Figure S4, next page).

7.1. Figure S3: Quantile-Quantile plots of smoking adjusted EWAS, in all participants.

<u>Figure S3:</u> Quantile-Quantile plots of cross-sectional covariate-adjusted EWAS (M_{smok}*) at first and second time point, in all participants on A) FEV_1 (inflation factor λ for time point 1 (λ = 1.02) and for time point 2 (λ = 1.04)); B) FEV_1/FVC (inflation factor λ for time point 1 (λ = 1.07) and for time point 2 (λ = 0.98)); and C) FVC (inflation factor λ for both time points (λ = 1.00)).

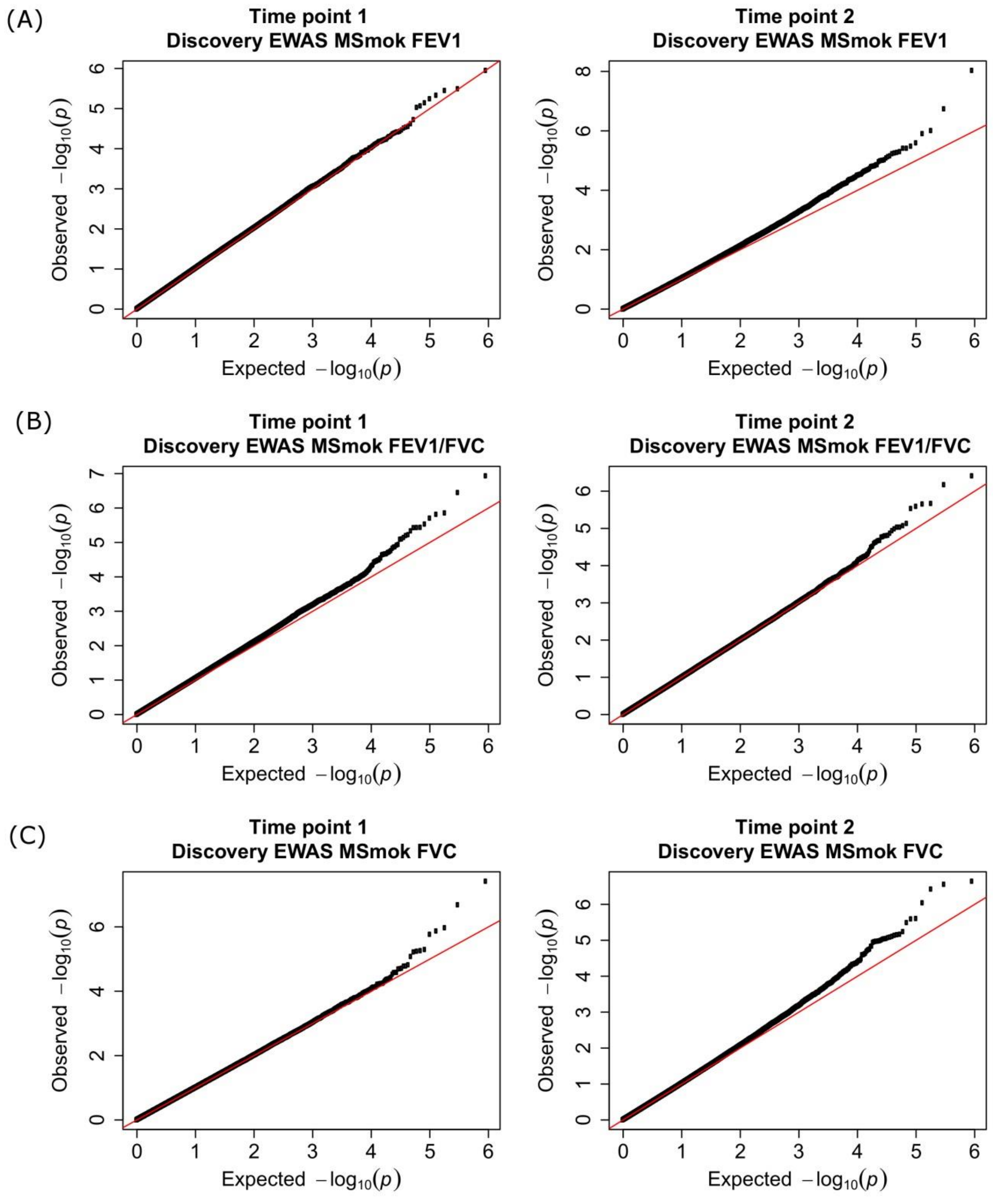

<u>Footnote to Figure S3:</u>

*Smoking adjusted model (M_{smok}): EWAS were adjusted for smoking covariates (history of smoking intensity as pack years smoked up to the time point of data collection for regressions and for smoking status (current, former and never smoker)) in addition to the base model covariate adjustments (age, age squared, height, squared deviation from the mean of height, sex and interaction terms of age, age squared, height and squared deviation of height with sex, education (low, medium, high), body mass index, spirometer type, study center as well as cell composition.

7.2. cg16288101: Smoking adjusted meta-analyses of cross-sectional associations (M_{smok}), in all participants.

Table S7: Meta-analyses of cross-sectional association with FEV_1, FEV_1/FVC, and FVC in all participants*, smoking adjusted EWAS (M_{smok}†). Meta-analyses of cross-sectional associations obtained using data from time point T2 of ECRHS, NFBC1966, SAPALDIA, LBC1936 and from time point T1 of KORA, LifeLines and NSPHS.

					Discovery meta-analysis at time point 2 (ECRHS,NFBC1966, SAPALDIA)				Replication meta-analysis (KORA,LBC1936, LifeLines, NSPHS, FTC)				Combined meta-analysis (ECRHS,NFBC1966, SAPALDIA, KORA,LBC1936, LifeLines, NSPHS)			
CpG ID	chr	positions	Locus	known smoking CpGs	beta (SE)	P-value meta-analysis	direction of effects	P-value between study hetero-geneity	beta (SE)	P-value meta-analysis	direction of effects	P-value between study hetero-geneity	beta (SE)	P-value meta-analysis	direction of effects	P-value between study hetero-geneity
**FEV1**																
cg07626482	19	47289503	*SLC1A5*	yes	1.826 (0.41)	8.23E-06	+++	0.690	1.086 (0.331)	0.0010	+-++-	0.259	1.612 (0.258)	4.32E-10	+++++++	0.919
cg03149958	6	36326677	*ETV7*	yes	1.319 (0.23)	9.31E-09	+++	0.339	0.337 (0.237)	0.1554	+-++-	0.303	0.896 (0.164)	4.32E-08	+++++++	0.069
cg05575921	5	373378	*AHRR*	yes	0.496 (0.146)	0.0007	+++	0.791	0.468 (0.123)	0.0001	++++-	0.356	0.496 (0.094)	1.27E-07	+++++++	0.758
cg18181703	17	76354621	*SOCS3*	yes	1.038 (0.248)	2.93E-05	+++	0.939	0.558 (0.2)	0.0052	++++-	0.512	0.819 (0.158)	2.14E-07	+++++++	0.894
**FEV1/FVC**																
cg05575921	5	373378	*AHRR*	yes	0.094 (0.021)	9.32E-06	+++	0.167	0.091 (0.018)	6.63E-07	+++++	0.993	0.094 (0.014)	2.21E-11	+++++++	0.726
cg03636183	19	17000585	*F2RL3*	yes	0.092 (0.03)	0.0022	+++	0.223	0.127 (0.026)	1.27E-06	++++-	0.570	0.117 (0.02)	1.02E-08	+++++++	0.343
cg21566642	2	233284661	*ALPPL2*	yes	0.084 (0.023)	0.0002	+++	0.862	0.091 (0.021)	1.57E-05	++++-	0.487	0.09 (0.016)	1.42E-08	+++++++	0.863
cg01940273	2	233284934	*ALPPL2*	yes	0.126 (0.031)	3.88E-05	+++	0.353	0.104 (0.029)	0.0003	+++++	0.630	0.12 (0.022)	2.75E-08	+++++++	0.620
cg23771366	11	86510998	*PRSS23*	yes	0.104 (0.035)	0.0034	+++	0.189	0.156 (0.03)	2.78E-07	+++++	0.993	0.13 (0.024)	4.61E-08	+++++++	0.598
cg03329539	2	233283329	*ALPPL2*	yes	0.107 (0.041)	0.0092	+++	0.737	0.143 (0.033)	1.30E-05	++++-	0.660	0.134 (0.026)	3.92E-07	+++++++	0.903
**FVC**																
cg03149958	6	36326677	*ETV7*	yes	1.315 (0.259)	3.78E-07	+++	0.484	0.654 (0.276)	0.0179	+-++-	0.161	1.08 (0.189)	1.07E-08	+++++++	0.401
cg08549335	7	30387954	*ZNRF2*	yes	0.953 (0.205)	3.24E-06	+++	0.441	0.359 (0.132)	0.0067	+++++	0.932	0.64 (0.121)	1.12E-07	+++++++	0.434

Footnote to table S7:
* Presentation of CpG markers showing meta-analysis P-value < $5x10^{-7}$ in the combined meta-analysis.
† Smoking model (M_{smok}) EWAS were covariate adjusted for age, age squared, height, squared deviation from the mean of height, sex and interaction terms of age, age squared, height and squared deviation of height with sex, education (low, medium, high), body mass index, spirometer type, study center as well as cell composition and additionally for adjusted for smoking covariates: history of smoking intensity as pack years smoked up to the time point of data collection for regressions and for smoking status (current, former and never smoker).

8. Results: Prediction of DNAme1 association with lung function (EWAS$_{predict}$)

8.1. Table S8: Prediction EWAS (M$_{base}$) for change in FEV$_1$ and for FVC, in all participants.

<u>Table S8:</u> Prediction EWAS meta-analyses* on annual change in lung function on FEV$_1$ and FVC, in all participants, base model adjustment (Mbase†). Replicated‡ associations indicated show replication P-value in bold.

						**Discovery (ECRHS/NFBC/SAPALDIA)**			**Replication (KORA, LBC1936)**				**Combined meta-analysis (ECRHS/NFBC/SAPALDIA/KORA,LBC1936)**		
**CpG ID**	**chr**	**position**	**Locus**	**smoking CpGs¶**	**smoking ¶ direction of effects**	**P-value meta-analysis**	**direction of effect**	**P-value (het)**	**P-value**	**direction of effect**	**replicated**	**P-value (het)**	**P-value meta-analysis**	**direction of effect (five studies)**	**P-value (het)**
**change in FEV1**									**P-value replication <0.00067‡**						
cg05575921	5	373378	*AHRR*	yes	6.1e-22 (-----------+----)	2.01E-15	+++	0.535	**4.89E-07**	++	**yes**	0.334	**1.96E-20**	+++++	0.318
cg01940273	2	233284934	*ALPPL2*	yes	9.8e-30 (----------------)	1.27E-12	+++	0.139	**6.32E-05**	++	**yes**	0.488	**5.63E-16**	+++++	0.263
cg21566642	2	233284661	*ALPPL2*	yes	4.5e-21 (----------------)	3.67E-13	+++	0.107	**0.0004**	++	**yes**	0.596	**7.16E-16**	+++++	0.307
cg03636183	19	17000585	*F2RL3*	yes	5.7e-17 (----------------)	5.64E-11	+++	0.511	**1.70E-05**	++	**yes**	0.139	**1.08E-14**	+++++	0.265
cg05951221	2	233284402	*ALPPL2*	yes	6.8e-23 (----------------)	5.81E-08	?++	0.377	**0.0002**	++	**yes**	0.447	**8.54E-11**	?++++	0.520
cg25648203	5	395444	*AHRR*	yes	2.7e-11 (----------------)	7.15E-09	+++	0.716	0.0040	++		0.018	**1.16E-10**	+++++	0.165
cg27241845	2	233250370	*ALPPL2*	yes	2.7e-10 (-----------+----)	2.85E-08	+++	0.077	0.0044	++		0.011	**4.44E-10**	+++++	0.021
cg26703534	5	377358	*AHRR*	yes	7.2e-18 (-----------+----)	5.87E-08	+++	0.477	0.0106	++		0.158	**2.15E-09**	+++++	0.466
cg21161138	5	399360	*AHRR*	yes	7.9e-13 (----------------)	1.43E-08	+++	0.742	0.0520	+-		0.010	**2.72E-09**	++++-	0.101
cg27537125	1	25349681	*RUNX3*	yes	5.5e-16 (-----------+----)	2.04E-08	+++	0.001	0.0554	++		0.187	**5.25E-09**	+++++	0.002
cg03329539	2	233283329	*ALPPL2*	yes	9.7e-16 (-----------+----)	3.59E-06	+++	0.169	0.0015	++		0.172	**2.49E-08**	+++++	0.206
cg12303084	20	45985741	*ZMYND8*	yes	4.2e-15 (---+------------)	1.66E-07	+++	0.454	0.0595	++		0.281	**4.05E-08**	+++++	0.469
cg21393163	1	12217629	*TNFRSF1B*	yes	3.8e-12 (----------------)	3.01E-07	+++	0.949	0.0484	++		0.767	**5.06E-08**	+++++	0.959
cg15342087	6	30720209	*FLOT1*	yes	3.9e-14 (-----------+----)	2.04E-07	+++	0.851	0.0816	++		0.045	**5.66E-08**	+++++	0.299
ch.8.91748119F	8	91678943				4.29E-08	+++	0.089	0.2817	++		0.060	**9.18E-08**	+++++	0.026
cg07986378	12	11898284	*ETV6*	yes	3.3e-07 (-----------+-+--)	2.11E-08	+++	0.364	0.4656	++		0.808	**2.03E-07**	+++++	0.136
cg00210249	10	71135679	*HK1*	yes	5.4e-06 (++++++++++++++++)	3.68E-07	---	0.738	0.5329	--		0.757	1.08E-06	-----	0.532
cg03149958	6	36326677	*ETV7*	yes	1.6e-08 (----------------)	1.39E-07	+++	0.228	0.9343	+-		0.657	1.54E-06	++++-	0.099
cg06762457	6	149806635	*ZC3H12D*	yes	1.1e-06 (-----------+----)	3.58E-07	+++	0.259	0.8538	+-		0.002	2.26E-05	++++-	4.44E-04
**change in FVC**									**P-value replication <0.0031‡**						
cg03149958	6	36326677	*ETV7*	yes	1.6e-08 (----------------)	**2.77E-10**	+++	0.325	0.7705	++		0.937	**3.37E-09**	+++++	0.125
cg05575921	5	373378	*AHRR*	yes	6.1e-22 (-----------+----)	**3.97E-07**	+++	0.025	0.3186	++		0.315	**3.68E-07**	+++++	0.055
cg03636183	19	17000585	*F2RL3*	yes	5.7e-17 (----------------)	**2.90E-07**	+++	0.080	0.4964	-+		0.146	5.72E-07	+++-+	0.062
cg15342087	6	30720209	*FLOT1*	yes	3.9e-14 (-----------+----)	**7.53E-08**	+++	0.026	0.9616	-+		0.698	8.98E-07	+++-+	0.016
cg01651915	8	55795551	*XKR4*	yes	2.1e-07 (----?------+-+--)	**3.86E-07**	+++	0.757	0.6393	+-		0.053	2.40E-06	++++-	0.091

Footnote to table S8:

* Prediction association of DNA methylation at first time point (DNAme1) with annual change in lung function during follow-up, defined as lung function at second time point – lung function at first time point divided by the time of follow-up in years. Presentation of CpG markers showing meta-analysis P-value < $5x10^{-7}$ at discovery or replication level. CpGs shown sorted by statistical significance of combined meta-analysis results.

† Base model (M_{base}) EWAS were covariate adjusted for age, age squared, height, lung function at time point 1 (FEV_1 or FVC respectively), squared deviation from the mean of height, sex and interaction terms of age, age squared, height and squared deviation of height with sex, education (low, medium, high), body mass index, spirometer type, study center as well as cell composition.

‡ Replication was defined for association with FEV_1 if replication P-value<0.00067 (multiple testing correction for 74 tests), and with FVC if replication P-value<0.0031 (multiple testing correction for 16 tests).

¶ Smoking CpGs defined on the reported FDR corrected P-value <0.05 for association reported with smoking status and reported direction of effects for association with smoking.[45]

8.2. Figure S4: Plots of prediction EWAS on FEV_1 and FVC

<u>Figure S4:</u> Manhattan and Quantile-Quantile plots of covariate-adjusted prediction* EWAS (M_{base}†) in all participants A) on FEV_1 (λ = 1.26); and B) on FVC (λ = 1.23).

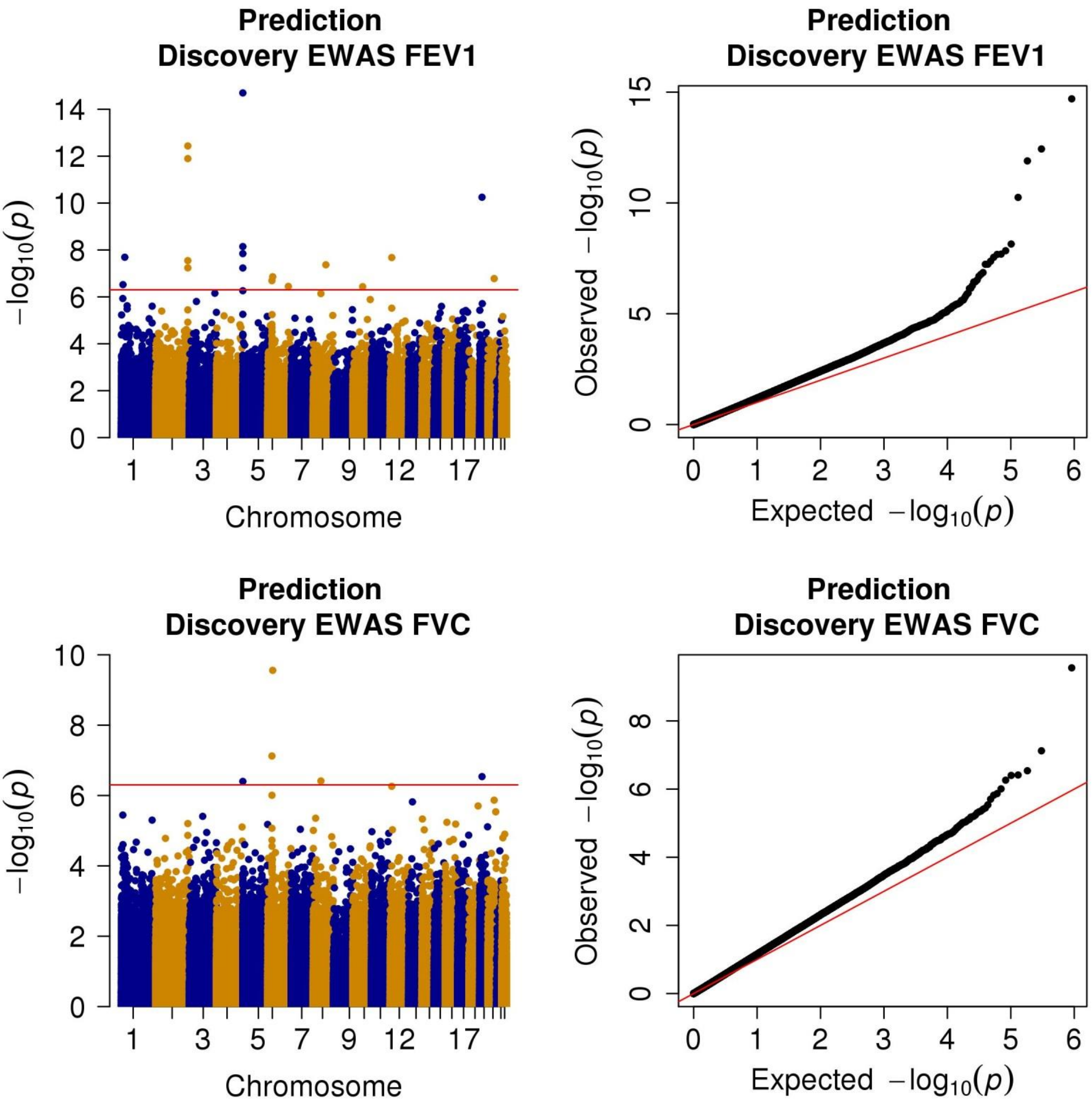

<u>Footnote to Figure S4:</u>

* Prediction association of DNA methylation at first time point (DNAme 1) with annual change in lung function during follow-up

†Base model (M_{base}): EWAS were covariate adjusted for age, age squared, height, lung function at time point 1 (FEV_1 or FVC respectively), squared deviation from the mean of height, sex and interaction terms of age, age squared, height and squared deviation of height with sex, education (low, medium, high), body mass index, spirometer type, study center as well as cell composition.

9. Results: EWAS meta-analyses in never smokers

9.1. Figure S5: Quantile-Quantile plots for cross-sectional associations, in never smokers.

<u>Figure S5:</u> Quantile-Quantile plots of cross-sectional covariate-adjusted EWAS (M_{base}*) at first and second time point, in never smokers A) on FEV_1 (inflation factor λ for time point 1 (λ = 1.09) and for time point 2 (λ = 1.05));B) FEV_1/FVC (inflation factor λ for time point 1 (λ = 1.11) and for time point 2 (λ = 0.96)); and C) FVC (inflation factor λ for time point 1 (λ = 1.08) and for time point 2 (λ = 1.03)).

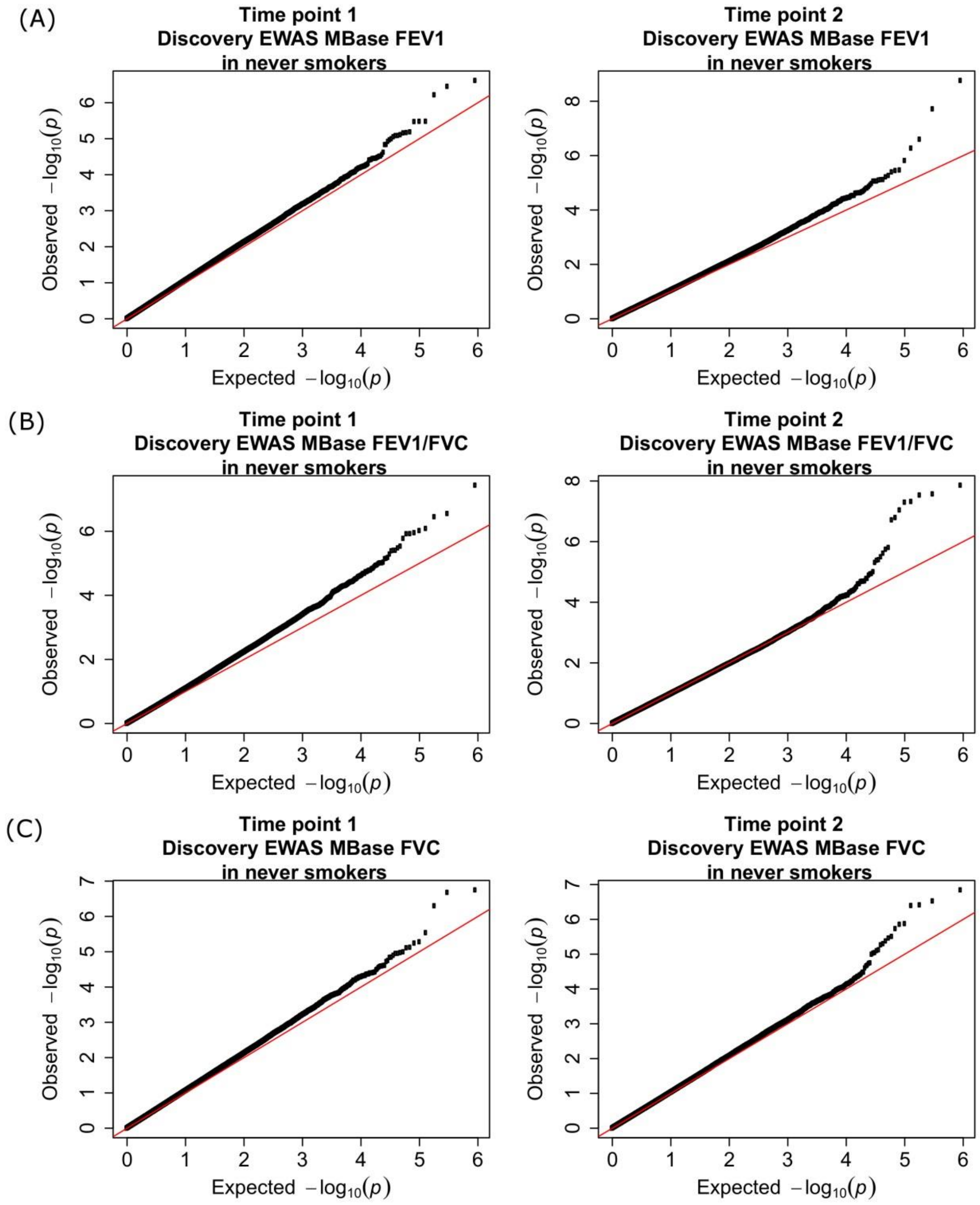

<u>Footnote to Figure S5:</u>

*Base model (M_{base}): EWAS were covariate adjusted for age, age squared, height, squared deviation from the mean of height, sex and interaction terms of age, age squared, height and squared deviation of height with sex, education (low, medium, high), body mass index, spirometer type, study center as well as cell composition.

9.2. Table S9: Cross-sectional associations, in never smokers.

<u>Table S9:</u> Detailed results in never smokers: cross-sectional associations with lung function at time point 1 and at time point 2, separately: discovery, replication and combined EWAS meta-analyses, base model covariate adjusted EWAS (M_{base}†). Meta-analyses of cross-sectional associations obtained using data from time point T2 of ECRHS, NFBC1966, SAPALDIA, LBC1936 and from time point T1 of KORA, LifeLines and NSPHS.
See table in EXCEL file: Addtional_Tables_Imbodenetal.xlsx

<u>Footnote to table S9:</u>
* Presentation of CpG markers showing meta-analysis P-value < $5x10^{-7}$ in the combined meta-analysis.
† Base model (M_{base}) EWAS were covariate adjusted for age, age squared, height, squared deviation from the mean of height, sex and interaction terms of age, age squared, height and squared deviation of height with sex, education (low, medium, high), body mass index, spirometer type, study center as well as cell composition.
‡ Smoking CpGs defined on the reported FDR corrected P-value <0.05 for association reported with smoking status and direction of effects.[45]

9.3. Table: EWAS$_{repeat}$ in never smokers:

In the repeat cross-sectional EWAS cg14366110 was the top hit ($P_{discovery}$=1.72 $x10^{-10}$) for association with FEV_1/FVC, yet this association did not replicate in the LBC1936 sample ($P_{replication}$= 0.454) though the direction of the effect was consistent and the $P_{combined}$ = 4.63 $x10^{-10}$ remained statistically epigenome-wide significant.

Table: EWAS$_{repeat}$ in never smokers: Discovery, replication and combined EWAS meta-analyses of repeat cross-sectional association with lung function in never smokers*, base model covariate adjusted EWAS (M_{base}†).

					Discovery (ECRHS/NFBC/SAPALDIA)				Replication (LCB1936)				Combined meta-analysis (ECRHS/NFBC/SAPALDIA/LBC1936)			
CpG ID	chr	position	Locus	known smoking CpG‡	beta (SE)	P-value meta-analysis	direction of effect	P-value study hetero-geneity	beta (SE)	P-value replication <0.00067	direction of effect	P-value study hetero-geneity	beta (SE)	P-value meta-analysis	direction of effect	P-value study hetero-geneity
**FEV1**																
cg17838734	6	83073924	*TPBG*	no	-2.507 (0.435)	**7.97E-09**	---	0.685	0.408 (0.502)	0.4169	+	na	-1.259 (0.329)	0.0001	---+	1.68E-04
cg19931644	8	12623485		no	0.917 (0.178)	**2.76E-07**	+++	0.695	0.121 (0.218)	0.5803	+	na	0.598 (0.138)	1.50E-05	++++	0.033
cg07922154	14	68087339	*ARG2*	no	1.098 (0.206)	**9.63E-08**	+++	0.299	0.193 (0.292)	0.5077	+	na	0.798 (0.168)	2.13E-06	++++	0.032
**FEV1/FVC**																
cg18938392	1	157248950		no	0.08 (0.014)	**2.87E-08**	+++	0.825	-0.009 (0.035)	0.7893	-	na	0.067 (0.013)	**4.59E-07**	+++-	0.121
cg15981995	3	169487311	*ARPM1*	no	-0.952 (0.188)	**4.29E-07**	??-	1.000	0.39 (0.309)	0.2063	+	na	-0.588 (0.161)	0.0003	??-+	2.06E-04
cg14366110	9	133779382	*FIBCD1*	no	-0.255 (0.04)	**1.72E-10**	---	0.904	-0.078 (0.104)	0.4539	-	na	-0.232 (0.037)	**4.63E-10**	----	0.440
**FVC**																
cg05831672	10	103543172	*NPM3*	no	-4.404 (0.802)	**3.93E-08**	---	0.871	-0.759 (1.345)	0.5727	-	na	-3.449 (0.689)	5.491E-07	----	0.127
cg22508172	1	24069723	*TCEB3*	no	-4.431 (0.836)	**1.17E-07**	---	0.582	-0.634 (1.22)	0.6033	-	na	-3.218 (0.69)	3.09E-06	----	0.053
cg10212705	1	154297848	*ATP8B2*	no	-5.875 (1.129)	**1.96E-07**	---	0.277	0.784 (3.445)	0.8200	+	na	-5.229 (1.073)	1.10E-06	---+	0.114
cg04030659	6	22570704	*HDGFL1*	no	-2.095 (0.412)	**3.66E-07**	---	0.442	0.419 (0.531)	0.4299	+	na	-1.15 (0.326)	0.0004	---+	0.001

Footnote table EWAS$_{repeat}$ in never smokers:

* Presentation of CpG markers showing meta-analysis P-value < $5x10^{-7}$ in the combined meta-analysis.

† Base model (M_{base}) EWAS were covariate adjusted for age, age squared, height, squared deviation from the mean of height, sex and interaction terms of age, age squared, height and squared deviation of height with sex, education (low, medium, high), body mass index, spirometer type, study center as well as cell composition.

‡ Smoking CpGs defined on the reported FDR corrected P-value <0.05 for association reported with smoking status and direction of effects.[45]

9.4. Table S10: Prediction EWAS, in never smokers.

<u>**Table S10:**</u> Prediction EWAS meta-analysis* on change in lung function in never smokers only, base model adjustment (M_{base}†).

					**Discovery (ECRHS/NFBC/SAPALDIA)**				**Replication (KORA/LCB1936)**				**Combined meta-analysis (ECRHS/NFBC/SAPALDIA/ KORA/LCB1936)**			
**CpG ID**	**chr**	**position**	**Locus**	**smoking CpGs**	**beta (SE)**	**P-value meta-analysis**	**direction of effect**	**P-value study hetero-geneity**	**beta (SE)**	**P-value replication**	**direction of effect**	**P-value study hetero-geneity**	**beta (SE)**	**P-value meta-analysis**	**direction of effect**	**P-value study hetero-geneity**
**FEV1**																
cg21393163	1	12217629	*TNFRSF1B*	yes	0.08 (0.025)	0.0013	+++	0.347	0.122 (0.054)	0.0242	++	0.443	0.087 (0.022)	0.0001	+++++	0.523
cg08447479	16	75589467	*TMEM231*	no	0.40 (0.078)	**3.28E-07**	+++	0.727	-0.135 (0.114)	0.2357	--	0.950	0.229 (0.065)	0.0004	+++--	0.004
**FEV1/FVC**																
cg14366110	9	133779382	*FIBCD1*	no	0.018 (0.003)	**4.24E-09**	++-	0.152	0.01 (0.013)	0.4390	++	0.414	0.017 (0.003)	**3.639E-09**	++-++	0.315
cg11216682	2	131113867	*PTPN18*	no	-0.018 (0.003)	**9.05E-08**	+-+	0.114	-0.006 (0.016)	0.6795	--	0.666	-0.017 (0.003)	**1.086E-07**	+-+--	0.282
**FVC**																
cg20098854	8	898407		no	-0.085 (0.016)	**1.32E-07**	---	0.108	0.059 (0.061)	0.3394	-+	0.291	-0.076 (0.016)	1.16E-06	----+	0.030
cg04774364	10	106100810		no	0.188 (0.036)	**1.62E-07**	+++	0.566	0.003 (0.071)	0.9635	+-	0.557	0.151 (0.032)	2.59E-06	++++-	0.145
cg20278790	20	57583474	*CTSZ*	no	0.24 (0.047)	**2.72E-07**	+++	0.004	-0.011 (0.094)	0.9071	+-	0.775	0.19 (0.042)	5.36E-06	++++-	0.002

<u>Footnote to table S10:</u>

* Prediction association of DNA methylation at first time point (DNAme 1) with annual change in lung function during follow-up, defined as lung function at second time point – lung function at first time point divided by the time of follow-up in years. Presentation of CpG markers showing meta-analysis P-value < $5x10^{-7}$ at discovery or replication level.

† Base model (M_{base}) EWAS were covariate adjusted for age, age squared, height, lung function at time point 1 (FEV_1, FVC or FEV_1/FVC respectively), squared deviation from the mean of height, sex and interaction terms of age, age squared, height and squared deviation of height with sex, education (low, medium, high), body mass index, spirometer type, study center as well as cell composition.

10. Results: Functional characterization of replicated CpGs

10.1. **Table S11:** 43 differentially methylated loci associated with lung function

Table S11: 43 Loci associated with lung function differentially methylated loci associated with lung function based on 57 replicated CpGs.

CpG ID	CHR	POSITION	NEAREST GENE	DISTANCE TO GENE	Strand	annotation analysis
cg04885881	1	11123118	*SRM*	-3027	F	used
cg21393163	1	12217629	*TNFRSF1B*	9429	R	used
cg27537125	1	25349681	*RUNX3*	-92911	F	used
cg21140898	1	51442318	*CDKN2C*	-2009	F	used
cg09935388	1	92947588	*GFI1*	0	F	used
cg18826637	2	145116633	*ZEB2*	25307	F	used
cg27241845	2	233250370	*ALPPL2*		R	pruned
cg17087741	2	233283010	*ALPPL2*		F	pruned
cg03329539	2	233283329	*ALPPL2*		F	pruned
cg05951221	2	233284402	*ALPPL2*		F	pruned
cg21566642	2	233284661	*ALPPL2*	-9237	R	used
cg01940273	2	233284934	*ALPPL2*		R	pruned
cg19859270	3	98251294	*GPR15*	0	R	used
cg00741986	4	2748332	*TNIP2*	0	R	used
cg08763102	4	3079751	*HTT*		F	pruned
cg24086068	4	77356008	*SHROOM3*	243	R	used
cg01899089	5	369969	*AHRR*		F	pruned
cg05575921	5	373378	*AHRR*	0	F	used
cg26703534	5	377358	*AHRR*		F	pruned
cg25648203	5	395444	*AHRR*		R	pruned
cg21161138	5	399360	*AHRR*		R	pruned
cg05673882	5	74862702	*POLK*	0	F	used
cg14753356	6	30720108	*intergenic*		R	pruned
cg24859433	6	30720203	*intergenic*		R	pruned
cg15342087	6	30720209	*FLOT1*	-7882	R	used
cg05593667	6	35490744	*TULP1*	-10097	F	used
cg03149958	6	36326677	*ETV7*	7292	R	used
cg00073460	6	149806502	*ZC3H12D*		F	pruned
cg06762457	6	149806635	*ZC3H12D*	-487	F	used
cg08549335	7	30387954	*ZNRF2*	0	R	used
cg25949550	7	145814306	*CNTNAP2*	0	F	used
cg19589396	8	103937374	*AZIN1*	-60977	F	used
cg12075928	8	141801307	*PTK2*	0	F	used
cg02716826	9	33447032	*AQP3*	0	R	used
cg04813697	10	22920025	*PIP4K2A*	0	R	used
cg25953130	10	63753550	*ARID5B*	0	F	used
cg00210249	10	71135679	*HK1*	0	R	used
cg03450842	10	80834947	*ZMIZ1*	0	F	used
cg21611682	11	68138269	*LRP5*	0	F	used
cg11660018	11	86510915	*PRSS23*		F	pruned
cg23771366	11	86510998	*PRSS23*	491	R	used
cg21990700	12	7260776	*C1RL*	0	R	used
cg07986378	12	11898284	*ETV6*	0	R	used

cg06826457	12	12867669	*P27_CRE*	2528	R	used
cg02583484	12	54677008	*HNRNPA1*	0	F	used
cg13976502	14	74227875	*ELMSAN1*	0	F	used
cg00310412	15	74724918	*SEMA7A*	0	R	used
cg16391678	16	30485597	*ITGAL*	0	F	used
cg01243823	16	50732212	*NOD2*	0	R	used
cg19572487	17	38476024	*RARA*	0	R	used
cg18181703	17	76354621	*SOCS3*	0	R	used
cg03636183	19	17000585	*F2RL3*	0	R	used
cg07626482	19	47289503	*SLC1A5*	0	F	used
cg03707168	19	49379127	*PPP1R15A*	0	F	used
cg12303084	20	45985741	*ZMYND8*	-267	F	used
cg23110422	21	40182073	*ETS2*	0	F	used
cg01127300	22	38614796	*TMEM184B*	500	F	used

10.2. Table S12: Functional annotation: Transcription binding sites

Table S12: Enrichment for transcription binding site of transcription factors

Transcription Factor	N expected	N observed	p-value	FDR p-value
RELA	2.5863	15	<1.00E-04	0.002
EP300	4.3584	18	<1.00E-04	0.004
POLR2A	12.787	29	<1.00E-04	0.012
EBF1	1.5989	10	<1.00E-04	0.012
IKZF1	0.3509	7	<1.00E-04	0.015
FOXM1	1.4265	9	<1.00E-04	0.021
POU2F2	1.6033	9	<1.00E-04	0.021
STAT3	1.8665	9	<1.00E-04	0.021
TBL1XR1	1.5904	9	<1.00E-04	0.021
RUNX3	2.4005	10	1.00E-04	0.039
PAX5	1.76	8	<1.00E-04	0.039

10.3. Table S13: Functional annotation: Chromatin State Models

Table S13: Enrichement for chromatin state model reported by Roadmap.

Roadmap ID	Chromatin State model	N expected	N observed	p-value	FDR p-value
E128 [NHLF Lung Fibroblast Primary Cells]	3 [Transcr. at gene 5′ and 3′]	0.4589	5	1.00E-04	0.06249932
E096 [Lung]	7 [Enhancers]	5.5423	14	7.00E-04	0.06356541
E017 [IMR90 fetal lung fibroblasts Cell Line]	7 [Enhancers]	2.989	9	0.0026	0.06542811
E034 [Primary T cells from peripheral blood]	2 [Flanking active TSS]	4.2305	12	2.00E-04	0.07681021
E128 [NHLF Lung Fibroblast Primary Cells]	7 [Enhancers]	2.9174	8	0.0083	0.10937309
E001 [ES-I3 Cells]	7 [Enhancers]	4.7668	11	0.0052	0.11846625
E088 [Fetal Lung]	6 [Genic enhancers]	0.3166	4	2.00E-04	0.12499918
E017 [IMR90 fetal lung fibroblasts Cell Line]	3 [Transcr. at gene 5′ and 3′]	0.505	4	0.0022	0.12499918

10.4. Table S14: Functional annotation: Canonical Pathways

Table S14: Canonical pathways (p-value < 0.05) from Ingenuity Pathway Analysis system.

Ingenuity Canonical Pathways	P-vaule	FDR	Ratio	Focused Genes
Aryl Hydrocarbon Receptor Signaling	0.004	0.16	0.022	AHRR,RARA,CDKN1B
Spermidine Biosynthesis I	0.005	0.16	0.500	SRM
Th2 Pathway	0.005	0.16	0.021	RUNX3,SOCS3,GFI1
Role of Oct4 in Mammalian Embryonic Stem Cell Pluripotency	0.005	0.16	0.044	ETS2,RARA
Semaphorin Signaling in Neurons	0.006	0.16	0.039	PTK2,SEMA7A
Regulation of Cellular Mechanics by Calpain Protease	0.007	0.16	0.036	PTK2,CDKN1B
Th1 and Th2 Activation Pathway	0.008	0.16	0.017	RUNX3,SOCS3,GFI1
Cell Cycle: G1/S Checkpoint Regulation	0.010	0.16	0.030	CDKN2C,CDKN1B
Agrin Interactions at Neuromuscular Junction	0.010	0.16	0.030	PTK2,ITGAL
Trehalose Degradation II (Trehalase)	0.011	0.16	0.200	HK1
Molecular Mechanisms of Cancer	0.012	0.16	0.010	PTK2,LRP5,CDKN2C,CDKN1B
VDR/RXR Activation	0.013	0.17	0.026	LRP5,CDKN1B
Cyclins and Cell Cycle Regulation	0.014	0.17	0.025	CDKN2C,CDKN1B
Small Cell Lung Cancer Signaling	0.016	0.17	0.024	PTK2,CDKN1B
IL-7 Signaling Pathway	0.017	0.17	0.023	PTK2,CDKN1B
Crosstalk between Dendritic Cells and Natural Killer Cells	0.018	0.17	0.023	TNFRSF1B,ITGAL
GDP-glucose Biosynthesis	0.020	0.18	0.111	HK1
Glucose and Glucose-1-phosphate Degradation	0.023	0.18	0.100	HK1
IGF-1 Signaling	0.025	0.18	0.019	PTK2,SOCS3
UDP-N-acetyl-D-galactosamine Biosynthesis II	0.025	0.18	0.091	HK1
Type I Diabetes Mellitus Signaling	0.025	0.18	0.019	SOCS3,TNFRSF1B
Paxillin Signaling	0.026	0.18	0.018	PTK2,ITGAL
HGF Signaling	0.028	0.18	0.018	PTK2,ETS2
Rac Signaling	0.029	0.18	0.017	PTK2,PIP4K2A
PTEN Signaling	0.030	0.18	0.017	PTK2,CDKN1B
RhoA Signaling	0.032	0.18	0.016	PTK2,PIP4K2A
Role of Macrophages, Fibroblasts and Endothelial Cells in Rheumatoid Arthritis	0.032	0.18	0.010	SOCS3,LRP5,TNFRSF1B
Telomere Extension by Telomerase	0.034	0.18	0.067	HNRNPA1
IL-6 Signaling	0.035	0.18	0.016	SOCS3,TNFRSF1B
Th1 Pathway	0.035	0.18	0.015	RUNX3,SOCS3
G_12/13 Signaling	0.038	0.19	0.015	PTK2,F2RL3
Inflammasome pathway	0.045	0.20	0.050	NOD2
Type II Diabetes Mellitus Signaling	0.048	0.20	0.013	SOCS3,TNFRSF1B
Polyamine Regulation in Colon Cancer	0.049	0.20	0.046	AZIN1
D-myo-inositol-5-phosphate Metabolism	0.049	0.20	0.013	SOCS3,PIP4K2A

10.5. Table S15: Functional annotation: Pathway analysis using KEGG

Table S15: Pathway analysis by topKEGG showing top 10 pathways.

Here, Pathway corresponds to the KEGG pathway being tested; N corresponds to number of genes in KEGG term; DE corresponds to the number of genes differentially methylated; P.DE corresponds to the p-values for overrepresentation of the KEGG terms; FDR corresponds to the false discovery rate corrected p-value.

Pathway	N	DE	P.DE	FDR
Transcriptional misregulation in cancer	180	4	1.02E-05	0.003
Pathways in cancer	515	5	2.91E-05	0.005
Regulation of actin cytoskeleton	207	3	0.0005	0.05
Type II diabetes mellitus	46	2	0.0009	0.07
Central carbon metabolism in cancer	65	2	0.002	0.09
TNF signaling pathway	108	2	0.002	0.09
Leukocyte transendothelial migration	110	2	0.002	0.09
Small cell lung cancer	93	2	0.002	0.09
Insulin signaling pathway	137	2	0.004	0.15
Cell adhesion molecules (CAMs)	139	2	0.005	0.16

10.6. Table S16: Functional annotation: GO term enrichment analysis

Table S16: GO enrichment analysis by topGO. Here, Term = the gene ontology term being tested; Ont = Ontology (here, BP = biological process) N = number of genes in GO term; DE = number of genes differentially methylated; P.DE = p-values for overrepresentation of the KEGG terms; FDR = False discovery rate

Term	Ontology	N	DE	P.DE	P_{FDR}
regulation of immune response	BP	800	9	6.28E-06	0.133
immune system process	BP	2268	14	1.82E-05	0.192
regulation of immune system process	BP	1238	10	5.23E-05	0.264
vitellogenesis	BP	4	2	6.86E-05	0.264
regulation of transcription from RNA polymerase II promoter	BP	1770	13	7.28E-05	0.264
positive regulation of immune response	BP	593	7	7.49E-05	0.264
positive regulation of immune system process	BP	853	8	0.0001	0.373
transcription from RNA polymerase II promoter	BP	1957	13	0.0002	0.435
cytoplasm organization	BP	10	2	0.0002	0.522
negative regulation of cellular macromolecule biosynthetic process	BP	1262	10	0.0003	0.540

11. Results: Enrichment analysis of smoking-related CpGs in discovery EWAS.

11.1. Table S17: Overrepresentation of smoking-related CpGs among the lung function associated CpG markers.

Table S17: Enrichment analysis results testing for overrepresentation of smoking-related CpGs among the lung function or prediction of change in lung function* associated CpG markers. P-values from Weighted Kolmogorov Smirnov Test (WKS).[54] P-values < 0.05 are considered statistically significant and shown in bold. Prior to WKS testing we excluded cross-reactive probes.[58]

		FEV1	FVC	FEV1/FVC
Cross-sectional time point 1	All, M_{base}†	**0.00036**	0.99	**0.00036**
	All, M_{smok}‡	**0.00053**	0.81	**0.00036**
	Never smokers, M_{base}†	**0.00036**	0.088	**0.00036**
Cross-sectional Time point 2	All, M_{base}†	**0.00036**	**0.00036**	**0.00036**
	All, M_{smok}‡	**0.00036**	**0.00082**	**0.0011**
	Never smokers, M_{base}†	**0.00036**	**0.0048**	0.23
Prediction Association*	All, M_{base}†	**0.00036**	**0.00036**	**0.00036**
	All, M_{smok}‡	0.71	0.078	0.25
	Never smokers, M_{base}†	0.65	**0.0026**	**0.049**

Footnote to table S17:

*Prediction association of DNA methylation at first time point (DNAme1) with annual change in lung function during follow-up, defined as lung function at second time point – lung function at first time point divided by the time of follow-up in years. Prediction models were additional adjusted for lung function at time point 1 (FEV_1, FVC or FEV_1/FVC respectively).

† Base model (M_{base}) EWAS were covariate adjusted for age, age squared, height, squared deviation from the mean of height, sex and interaction terms of age, age squared, height and squared deviation of height with sex, education (low, medium, high), body mass index, spirometer type, study center as well as cell composition.

‡ Smoking model EWAS (M_{smok}) were additionally adjusted for smoking covariates: history of smoking intensity as pack years smoked up to the time point of data collection for regressions and for smoking status (current, former and never smoker).

12. Results: Associations of sentinel CpGs with lung function in childhood cohorts (ALSPAC, IOWBC)

12.1. Table S18: Associations of sentinel CpGs with FEV_1, FEV_1/FVC, and FVC in childhood birth cohorts, ALSPAC and IOWBC

Table S18: Adjusted* associations of sentinel replication CpG markers† with FEV_1, FEV_1/FVC, and FVC in childhood birth cohorts, IOWBC and ALSPAC. The list of CpGs represent markers with suggestive evidence‡ for association in children (P≤0.05) with cross-sectional¶ lung function, FEV_1 (L), FEV_1/FVC and FVC (L), and prediction of annual change, FEV_1 (L/year), FEV_1/FVC(year^{-1}) and FVC (L/year). Boxed coefficients beta(SE) indicate consistency with meta-analyses results observed in adults for direction of effect (outcome and model specific comparison of childhood results with adult meta-analyses combining discovery and replication cohort results). Underlined CpGs showed successfully replication in adults. Dark grey-boxed CpGs showed associations observed in adult never smokers.

						cross-sectional time point 1				cross-sectional time point 2				repeat cross-sectional				prediction in annual change			
CpG ID	chr	position	Locus	known smoking CpG	Best P-value in adults**	beta (SE)	P-value meta-analysis	sign	P-value between study hetero-geneity	beta (SE)	P-value meta-analysis	sign	P-value between study hetero-geneity	beta (SE)	P-value meta-analysis	sign	P-value between study hetero-geneity	beta (SE)	P-value meta-analysis	sign	P-value between study hetero-geneity
**FEV1**																					
cg27537125	1	25349681	*RUNX3*	yes	5.28E-13	1.399 (0.695)	**0.044**	-+	0.16	1.532 (1.564)	0.327	++	0.54	1.384 (0.873)	0.113	++	0.83	-0.073 (0.177)	0.680	--	0.45
cg03547355	1	227003060		yes	1.54E-10	-0.353 (0.217)	0.104	--	0.34	-0.709 (0.456)	0.120	--	0.44	-0.551 (0.235)	**0.019**	--	0.79	0.044 (0.079)	0.578	++	0.72
cg27241845	2	233250370	*ALPPL2*	yes	1.63E-21	-0.115 (0.098)	0.241	--	0.41	-0.291 (0.269)	0.278	--	0.91	-0.309 (0.15)	**0.040**	--	0.45	0.059 (0.041)	0.147	++	0.71
cg01940273	2	233284934	*ALPPL2*	yes	3.30E-34	-0.131 (0.243)	0.591	--	0.91	-1.161 (0.513)	**0.024**	--	0.23	-0.656 (0.292)	**0.025**	--	0.47	-0.003 (0.083)	0.967	+-	0.01
cg00741986	4	2748332	*TNIP2*	yes	6.52E-10	0.054 (0.198)	0.785	+-	0.46	-1.187 (0.54)	**0.028**	--	0.67	-0.454 (0.228)	**0.046**	--	0.81	-0.112 (0.066)	0.091	--	0.09
cg25648203	5	395444	*AHRR*	yes	2.87E-17	-0.609 (0.257)	**0.018**	--	0.47	0.139 (0.683)	0.839	+-	0.78	-0.212 (0.327)	0.517	--	0.67	0.045 (0.096)	0.637	-+	0.27
cg23205886	5	138611766	*SNHG4*	yes	5.63E-11	0.483 (0.229)	**0.035**	++	0.50	0.108 (0.661)	0.870	++	0.85	-0.117 (0.303)	0.700	-+	0.48	0.077 (0.084)	0.355	++	0.59
cg19931644	8	12623485			2.75E-07	-0.299 (0.144)	**0.037**	-+	0.10	0.017 (0.334)	0.960	-+	0.61	-0.054 (0.22)	0.807	-+	0.11	-0.011 (0.049)	0.826	-+	0.51
cg21990700	12	7260776	*C1RL*	yes	1.06E-11	0.222 (0.29)	0.445	+-	0.25	1.463 (0.619)	**0.018**	++	0.16	0.547 (0.405)	0.178	-+	0.05	0.083 (0.092)	0.368	++	0.76
cg16708465	13	95933097	*ABCC4*	yes	1.15E-08	0.036 (0.269)	0.893	-+	0.53	1.066 (0.629)	0.090	++	0.30	0.700 (0.344)	**0.042**	++	0.44	-0.06 (0.102)	0.553	--	0.80
cg07922154	14	68087339	*ARG2*		9.63E-08	0.193 (0.203)	0.341	++	0.87	1.187 (0.586)	**0.043**	++	0.69	0.17 (0.294)	0.563	++	0.63	0.078 (0.078)	0.314	++	0.44
cg13976502	14	74227875	*C14orf43*	yes	7.86E-12	-0.518 (0.234)	**0.027**	--	0.47	-0.254 (0.574)	0.658	--	0.88	-0.183 (0.314)	0.561	-+	0.10	0.003 (0.081)	0.968	+-	0.83
cg00310412	15	74724918	*SEMA7A*	yes	4.15E-15	-0.177 (0.266)	0.506	--	0.59	-0.905 (0.688)	0.188	--	0.45	-0.872 (0.327)	**7.69E-03**	--	0.42	0.063 (0.098)	0.524	+-	0.32
cg05557932	16	3929351	*CREBBP*	yes	2.99E-08	-0.733 (0.371)	**0.048**	--	0.95	0.547 (0.657)	0.406	++	0.84	0.359 (0.427)	0.400	+-	0.37	-0.032 (0.103)	0.754	-+	0.02
cg20278790	20	57583474	*CTSZ*		1.62E-07	0.361 (0.262)	0.167	++	0.02	-1.442 (0.708)	**0.042**	--	0.43	-0.566 (0.384)	0.141	-+	0.04	0.202 (0.101)	**0.045**	+-	0.02
cg01127300	22	38614796		yes	1.19E-12	-0.354 (0.181)	**0.050**	--	1.00	-0.244 (0.387)	0.527	--	0.54	-0.164 (0.218)	0.452	--	0.69	-0.021 (0.066)	0.751	-+	0.24
**FEV1/FVC**																					
cg18664508	3	169487465	*ARPM1*		1.92E-08	-0.213 (0.249)	0.394	--	0.98	-0.38 (0.317)	0.230	+-	0.25	-0.449 (0.195)	**0.022**	--	0.89	0 (0.035)	1.000	+-	0.94
cg15930777	6	12343201			4.88E-07	0.078 (0.061)	0.201	++	0.87	0.14 (0.058)	**0.015**	++	0.52	0.114 (0.04)	**4.05E-03**	++	0.51	-0.017 (0.01)	0.093	--	0.49
cg15342087	6	30720209	*FLOT1*	yes	5.44E-24	-0.036 (0.119)	0.765	-+	0.22	0.292 (0.135)	**0.030**	++	0.53	0.099 (0.068)	0.144	++	0.22	-0.01 (0.02)	0.624	+-	0.34
cg14366110	9	133779382	*FIBCD1*		1.72E-10	0.295 (0.121)	**0.014**	++	0.95	-0.113 (0.117)	0.335	--	0.77	0.053 (0.068)	0.443	++	0.57	-0.036 (0.019)	0.061	--	0.93
cg09884077	15	23086698	*NIPA1*		1.38E-08	-0.504 (0.296)	0.088	--	0.24	-0.748 (0.287)	**9.03E-03**	--	0.90	-0.471 (0.203)	**0.021**	--	0.17	-0.019 (0.042)	0.658	+-	0.18
cg22952142	15	68549178			4.03E-07	0.125 (0.056)	**0.025**	+-	0.05	0.109 (0.061)	0.074	++	0.34	0.141 (0.043)	**1.08E-03**	++	0.33	-0.009 (0.009)	0.302	-+	0.06
cg27367615	16	86229910			3.31E-07	-0.05 (0.052)	0.336	--	0.65	-0.084 (0.058)	0.144	--	0.86	-0.079 (0.035)	**0.023**	--	0.89	0.003 (0.008)	0.746	-+	0.47
**FVC**																					
cg23205886	5	138611766	*SNHG4*	yes	5.63E-11	0.547 (0.276)	**0.048**	++	0.28	-0.439 (0.729)	0.547	+-	0.14	-0.2 (0.332)	0.547	--	0.57	0.072 (0.095)	0.445	+-	0.56
cg25633955	8	1616622	*DLGAP2*		3.87E-07	0.143 (0.192)	0.457	++	0.30	-1.416 (0.686)	**0.039**	--	0.66	-0.134 (0.289)	0.642	--	0.44	0.082 (0.073)	0.261	++	0.16
cg25953130	10	63753550	*ARID5B*	yes	1.55E-07	0.032 (0.163)	0.845	+-	0.76	0.071 (0.373)	0.849	++	0.94	-0.084 (0.225)	0.710	+-	0.17	0.126 (0.056)	**0.025**	++	0.72
cg05831672	10	103543172	*NPM3*		3.93E-08	1.23 (1.294)	0.342	++	0.57	3.154 (2.414)	0.191	++	0.62	3.009 (1.485)	**0.043**	++	0.93	-0.028 (0.34)	0.936	-+	0.10
cg01747591	16	89703612	*DPEP1*		4.20E-07	-0.408 (0.408)	0.317	--	0.84	-1.985 (0.842)	**0.019**	--	0.40	-1.422 (0.474)	**2.71E-03**	--	0.17	-0.101 (0.149)	0.497	-+	0.02
cg20278790	20	57583474	*CTSZ*		1.62E-07	0.649 (0.317)	**0.041**	++	0.04	-1.5 (0.768)	0.051	-+	0.24	-0.425 (0.424)	0.316	-+	0.09	0.227 (0.114)	**0.047**	+-	0.17

Footnote to table S18:

* Associations were adjusted for base model covariate adjustment (M_{base}): age, age squared, height, squared deviation from the mean of height, sex and interaction terms of age, age squared, height and squared deviation of height with sex, education (low, medium, high), body mass index, spirometer type, study center as well as cell composition. Prediction models were additionally adjusted for lung function at time point 1 (FEV_1, FEV_1/FVC or FVC respectively).

† Sentinel CpGs: Lung function-specific replication markers were selected for their association with $P<5x10^{-7}$ in discovery meta-analysis from the entire samples of all participants or from the subsample of never smokers.

‡ Associations in children replication sample showing nominal P≤0.05 (shaded light grey) are presented in the table.

¶ Cross-sectional associations tested: linear regression of cross-sectional association of DNA methylation marker with lung function parameter separately at each time point, (time point 1, time point 2) and mixed linear regression with random intercept on the participant of repeated cross-sectional association combining data from both time points in one model.

** The best P-value for association of the lung function-specific replication CpG with the corresponding lung function parameter observed in adults, either with FEV_1, FEV_1/FVC or FVC (as presented in Appendix all participants and never smokers (table S2; table S3, table S4, table S5).

13. Results: ALEC discovery meta-analysis look-up results of CpGs previously associated with respiratory traits

13.1. Table S19: Results for cross-sectional adjusted associations (M_{base}) with FEV_1, FEV_1/FVC and FVC at time point 2

<u>**Table S19:**</u> ALEC discovery meta-analysis look-up results for cross-sectional adjusted* associations at time point 2, base covariate adjustment (M_{base}).

See table in EXCEL file: Additional_Tables_Imbodenetal.xlsx

<u>*Footnote to table S19:*</u>

* Base model (M_{base}) EWAS were covariate adjusted for age, age squared, height, squared deviation from the mean of height, sex and interaction terms of age, age squared, height and squared deviation of height with sex, education (low, medium, high), body mass index, spirometer type, study center as well as cell composition.

14. Results: Two-sample Mendelian Randomization look-up in publicly available databases

We found genetic instruments for 13 of the 57 replicated CpGs and finally only for eight CpGs a two sample MR on cross-sectional lung function could be completed. Results for FEV_1 are presented in table S20. There was evidence for causal effect of cg23771366, cg11660018 (*PRSS23*) and cg21990700 (*C1RL*) and cg00073460 (*ZC3H12D*) on FEV_1 percent predicted and of cg00073460 (*ZC3H12D*) and cg24086068 (*SHROOM3*) on FVC. Of the four top loci associated with FEV_1/FVC (*AHRR*, *F2RL3*, *ALPPL2* and *PRSS23*, see table 2) we could only identify MR-genetic instruments for *ALPPL2* and *PRSS23* and only the association of *PRSS23* with lung function could be obtained from the MRbase resource.

The top CpG of the MR look-up showing causal association with cross-sectional lung function is also one of the three dominant smoking-related CpGs which remained significantly associated with lung function (FEV_1/FVC) after smoking adjustment though the strength of the association was greatly diminished: cg23771366 (*PRSS23)* had P=4.61x10^{-8} (β(SE)= 0.130(0.024); M_{smok} see table S7) versus P=5.38x10^{-27} (β(SE)= 0.233(0.022); M_{base} see table 2). Both other dominant smoking-related CpGs which also remained significant after smoking adjustment (cg05575921 (*AHRR)* having P=2.69x10^{-11} (M_{smok}) versus P=7.22^{-50} (M_{base}) and cg03636183 (*F2RL3*) having P=1.02x10^{-8} (M_{smok}) versus P=4.5x10^{-43} (M_{base})) were not available in the mQTL database and thus their causal association with lung function remains to be investigated.

14.1. Table S20: Two-sample MR results for FEV_1 predicted percentage and for FVC

<u>**Table S20A:**</u> Two-sample Mendelian Randomization for association with FEV_1 predicted percentage*

CpG ID/Exposure	instrument	chr	position	Locus	Beta†	SE	P-value	P-value$_{FDR}$‡	CpG part of Mediation-SI	CpG associated with (EWAS results)
cg23771366	rs67939314	11	86510998	***PRSS23***	0.06001	0.01358	**9.92E-06**	**4.76E-04**	no	Cross-sectional FEV1/FVC<br>Cross-sectional FEV1
cg11660018	rs36061072	11	86510915	***PRSS23***	0.0484	0.01464	**9.48E-04**	**0.015**	no	Cross-sectional FEV1/FVC<br>Cross-sectional FEV1
cg21990700	rs3782925	12	7260776	***C1RL***	-0.04477	0.01431	**1.76E-03**	**0.018**	no	Cross-sectional FEV1
cg00073460	rs12660849	6	149806502	***ZC3H12D***	-0.04814	0.01681	**4.18E-03**	**0.029**	no	Cross-sectional FEV1
cg24086068	rs17001890	4	77356008	*SHROOM3*	0.03555	0.0153	0.020	0.054	no	Cross-sectional FEV1<br>Cross-sectional FVC
cg09935388	rs115427247	1	92947588	*GFI1*	0.03309	0.01665	0.047	0.094	yes	Cross-sectional FEV1/FVC
cg05593667	rs7755718	6	35490744	*TULP1*	0.01534	0.008361	0.067	0.114	no	Cross-sectional FEV1

Footnote to table S20A:

* **FEV_1:** Using UKB-a:235 dataset on genome-wide association results on forced expiratory volume in 1-second (FEV_1) predicted percentage in European Population (n= 11,0423), Data from http://www.nealelab.is/blog/2017/9/11/details-and-considerations-of-the-uk-biobank-gwas.

† beta: effect estimate represents the change in outcome per unit increase in the methylation of the CpG. SE: standard error

‡FDR: False discovery rate adjusted P-value for multiple testing correction.

Table S20B: Two-sample Mendelian Randomization for association with FVC*

CpG ID/Exposure	instrument	chr	position	Locus	Beta†	SE	P-value	$P\text{-value}_{FDR}$‡	CpG part of Mediation-SI	CpG associated with (EWAS results)
cg00073460	rs12660849	6	149806502	***ZC3H12D***	-0.02163	0.007787	5.47E-03	**0.030**	no	Cross-sectional FEV1
cg24086068	rs17001890	4	77356008	***SHROOM3***	0.01939	0.007095	6.28E-03	**0.030**	no	Cross-sectional FEV1<br>Cross-sectional FVC
cg21990700	rs3782925	12	7260776	*C1RL*	-0.01461	0.006627	0.027	0.064	no	Cross-sectional FEV1
cg05593667	rs7755718	6	35490744	*TULP1*	0.008383	0.003879	0.031	0.064	no	Cross-sectional FEV1
cg11660018	rs36061072	11	86510915	*PRSS23*	0.007944	0.006823	0.244	0.366	no	Cross-sectional FEV1/FVC<br>Cross-sectional FEV1
cg09935388	rs115427247	1	92947588	*GFI1*	-0.003923	0.007859	0.618	0.760	yes	Cross-sectional FEV1/FVC
cg23771366	rs67939314	11	86510998	*PRSS23*	0.000751	0.006306	0.905	0.910	no	Cross-sectional FEV1/FVC<br>Cross-sectional FEV1

Footnote to table S20B:

* **FVC:** Using UKB-a:336 dataset on genome-wide association results on forced vital capacity (FVC) in European Population (n= 30,7638), Data from http://www.nealelab.is/blog/2017/9/11/details-and-considerations-of-the-uk-biobank-gwas.

† beta: effect estimate represents the change in outcome per unit increase in the methylation of the CpG. SE: standard error

‡FDR: False discovery rate adjusted P-value for multiple testing correction.

15. Results: Mediation analysis and Mediation smoking index (Mediation-SI)

15.1. Table S21: List of smoking effect mediating candiate CpGs and CpG list of mediation smoking index (Mediation-SI)

Table S21: List of CpGs* contributing to the Mediation-SI, sorted by P-value for association with smoking (Smoking P_{FDR}). The previously reported candidate CpG for mediation were looked-up in the meta-analyzed repeat cross-sectional covariate M_{base}-adjusted EWAS meta-analysis in discovery cohorts (ECRHS, NFBC1966 and SAPALDIA) combining data on methylation and spirometry of both time points.

CpG ID*	chr	position	Locus	Smoking P_{FDR}†	Smoking direction of effects†	Rank in $EWAS_{repeat}$‡	FEV1/FVC $EWAS_{repeat}$‡ P-value	FEV1/FVC $EWAS_{repeat}$‡ direction of effects
cg01940273	2	233'284'934	*ALPPL2*	9.80E-30	----------------	5	3.10E-11	+++
cg05951221	2	233'284'402	*ALPPL2*	6.80E-23	----------------	34	1.59E-06	??+
cg05575921	5	373'378	*AHRR*	6.10E-22	-----------+----	1	3.59E-16	+++
cg21566642	2	233'284'661	*ALPPL2*	4.50E-21	----------------	4	1.39E-11	+++
cg06126421	6	30'720'080	*IER3*	1.70E-20	----------------	31	1.25E-06	??+
cg03636183	19	17'000'585	*F2RL3*	5.70E-17	----------------	2	5.25E-13	+++
cg09935388	1	92'947'588	*GFI1*	7.00E-14	-----------+----	19	3.60E-07	+++
cg21161138	5	399'360	*AHRR*	7.90E-13	----------------	7	1.62E-10	+++
cg24859433	6	30'720'203	*NA*	2.20E-09	----------------	61	3.58E-06	+++
cg22994830	7	623'846	*PRKAR1B*	0.0001	+++++++++++-++--	414635	0.488	-+-

Footnote to table S21:

* set of ten CpG markers previously identified to be associated with smoking and to mediate the effect of smoking on lung function [46]. Two CpGs (cg05951221 and cg06126421) were present only on 450K array and thus missing in NFBC1966 data from time point 2 and in ECRHS data from both time points. Values for Mediation-SI score for missing data for cg05951221 and cg06126421 were imputed to mean of never-smokers in each cohort.

† Smoking CpGs defined on the reported FDR corrected P-value <0.05 for association reported with smoking status and reported direction of effects for association with smoking. [45]

‡ $EWAS_{repeat}$: Discovery meta-analysis of repeat cross-sectional association combining data from time point1 and time point 2 using mixed linear regression with a random intercept on the study participant.

15.2. Table S22: Replication of mediation analyses on FEV1 and on FVC in SAPALDIA

table S22: Mediation* analysis results for the effect of smoking status on FEV1 and on FVC .

CpG	ACME			ADE			Total effect			Proportion		
	Estimate	95%CI	p	Estimate	95%CI	p-value	Estimate	95%CI	p	Estimate	95%CI	p
**FEV1**												
cg01940273	-0.0563	[-0.0826, -0.0306]	**<0.001**	0.0051	[-0.0600, 0.0729]	0.89	-0.0511	[-0.1127, 0.0133]	0.104	1.0043	[-4.6356, 7.6423]	0.104
cg03636183	-0.0627	[-0.0905, -0.0368]	**<0.001**	0.0118	[-0.0515, 0.0754]	0.712	-0.0508	[-0.1113, 0.0057]	0.074	1.1768	[-4.5880, 13.3634]	0.074
cg05575921	-0.0768	[-0.1086, -0.0463]	**<0.001**	0.0264	[-0.0426, 0.0934]	0.432	-0.0504	[-0.1121, 0.0082]	0.098	1.3890	[-9.4984, 15.421]	0.098
cg05951221	-0.0587	[-0.0907, -0.0284]	**<0.001**	0.0087	[-0.0571, 0.0756]	0.794	-0.0499	[-0.1115, 0.0106]	0.108	1.0660	[-6.8504, 9.6637]	0.108
cg06126421	-0.0512	[-0.0793, -0.0273]	**<0.001**	0.0014	[-0.0603, 0.0642]	0.980	-0.0497	[-0.1090, 0.0098]	0.110	0.9142	[-6.2106, 6.7037]	0.110
cg09935388	-0.0257	[-0.0428, -0.0097]	**<0.001**	-0.0243	[-0.0835, 0.0378]	0.430	-0.0500	[-0.1069, 0.0135]	0.100	0.4855	[-1.8394, 4.0124]	0.100
cg21161138	-0.0398	[-0.0625, -0.0202]	**<0.001**	-0.0105	[-0.0748, 0.0505]	0.722	-0.0503	[-0.1110, 0.0079]	0.082	0.7532	[-3.1370, 6.8135]	0.082
cg21566642	-0.0681	[-0.0989, -0.0400]	**<0.001**	0.0193	[-0.0451, 0.0833]	0.600	-0.0489	[-0.1079, 0.0138]	0.106	1.2615	[-5.6624, 13.9565]	0.106
cg22994830	-0.0011	[-0.0054, 0.0013]	0.516	-0.0481	[-0.1100, 0.0085]	0.116	-0.0493	[-0.1110, 0.0078]	0.112	0.0131	[-0.1594, 0.2437]	0.568
cg24859433	-0.0264	[-0.0449, -0.0094]	**<0.0010**	-0.0223	[-0.0838, 0.0405]	0.484	-0.0487	[-0.1053, 0.0176]	0.128	0.4841	[-2.5739, 6.4572]	0.128
**FVC**												
cg01940273	-0.0424	[-0.0701, -0.0162]	**<0.001**	0.0228	[-0.0483, 0.0954]	0.508	-0.0197	[-0.0889, 0.0481]	0.562	0.8896	[-15.335, 16.3599]	0.562
cg03636183	-0.0451	[-0.0723, -0.0178]	**<0.001**	0.0260	[-0.0429, 0.0948]	0.444	-0.0191	[-0.0847, 0.0474]	0.536	1.0267	[-14.3319, 26.8314]	0.536
cg05575921	-0.0555	[-0.0872, -0.0242]	**<0.001**	0.0374	[-0.0324, 0.1110]	0.308	-0.0181	[-0.0854, 0.0470]	0.556	1.2442	[-15.3577, 28.1417]	0.556
cg05951221	-0.0467	[-0.0808, -0.0164]	**0.002**	0.0266	[-0.0441, 0.0993]	0.450	-0.0201	[-0.0825, 0.0441]	0.528	1.0274	[-14.6674, 17.8390]	0.530
cg06126421	-0.0437	[-0.0706, -0.0194]	**0.002**	0.0255	[-0.0422, 0.0953]	0.468	-0.0182	[-0.0822, 0.0439]	0.574	0.9103	[-17.7712, 19.6406]	0.572
cg09935388	-0.0178	[-0.0346, -0.0011]	**0.036**	0.0000	[-0.0666, 0.0691]	0.996	-0.0178	[-0.0813, 0.0484]	0.582	0.3412	[-7.2647, 7.4008]	0.602
cg21161138	-0.0245	[-0.0481, -0.0030]	**0.024**	0.0078	[-0.0622, 0.0776]	0.776	-0.0167	[-0.0835, 0.0506]	0.616	0.4701	[-10.8600, 14.6598]	0.620
cg21566642	-0.0499	[-0.0805, -0.0188]	**<0.001**	0.0295	[-0.0412, 0.1000]	0.424	-0.0204	[-0.0851, 0.0442]	0.580	0.9974	[-28.0538, 20.7039]	0.580
cg22994830	-0.0007	[-0.0050, 0.0018]	0.630	-0.0201	[-0.0898, 0.0439]	0.570	-0.0208	[-0.0899, 0.0440]	0.558	0.0036	[-0.6289, 0.4025]	0.864
cg24859433	-0.0259	[-0.0473, -0.0076]	**0.012**	0.0070	[-0.0607, 0.0766]	0.816	-0.0189	[-0.0853, 0.0471]	0.576	0.5115	[-8.4278, 7.0896]	0.584

Footnote to table S22

* performed using R package mediation [59].

† previously reported candidate CpG for mediation of smoking effect on lung function [46].

For analogous results for FEV1 or FVC see online supplementary table S22.

Abbreviations: ACME – average causal mediation effect; ADE – average direct effect.

15.3. Figure S6: Association of Mediation-SI with FEV_1 and comparison with association of packyears SI with FEV_1

__Figure S6:__ Distribution and association* of Mediation-SI with FEV_1, with 95% confidence interval. (A) Boxplot of Mediation-SI (median: 0.3 and range: -1.7 to 5.2) in all participants of SAPALDIA. (B) Boxplot of packyears (median: 2.0 and range: 0 to 145.9) in all participants of SAPALDIA

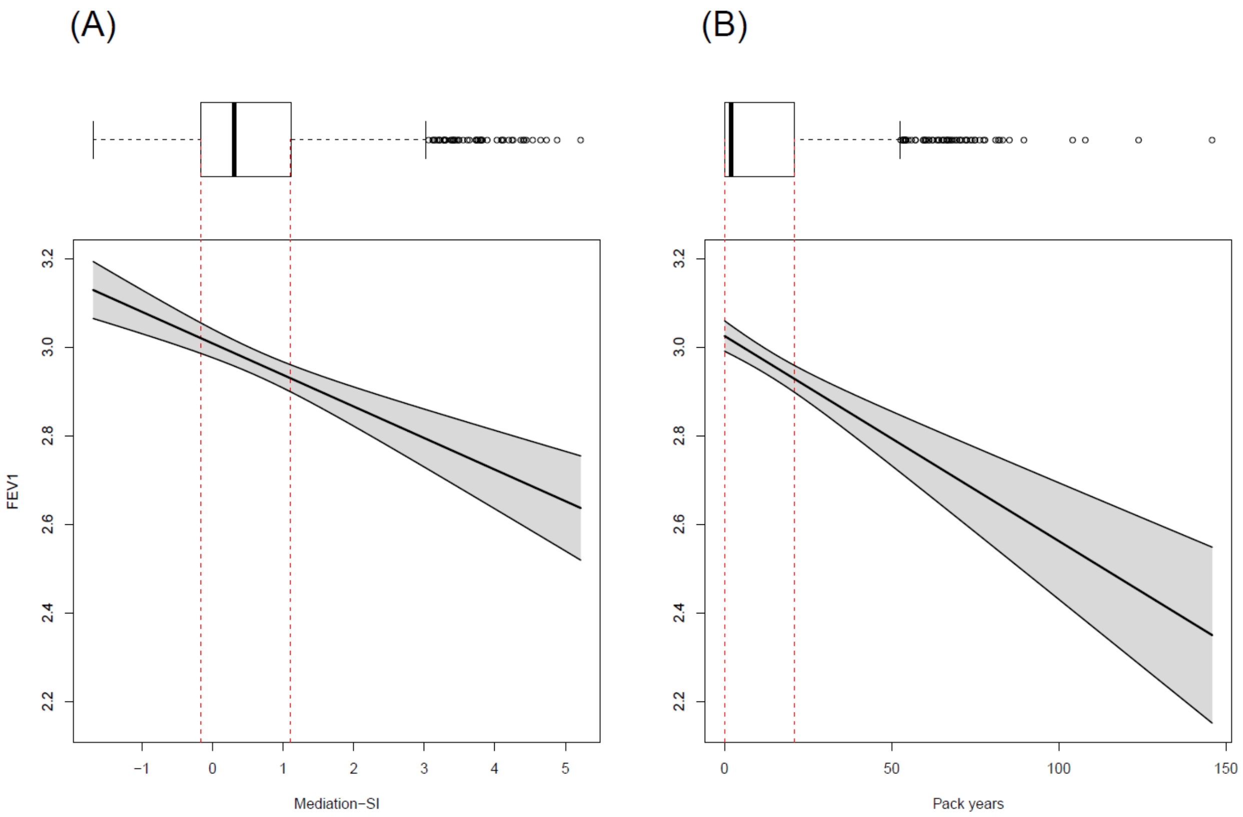

__Footnote to Figure S6:__

*Associations were adjusted for the base model (M_{base}): age, age squared, height, squared deviation from the mean of height, sex and interaction terms of age, age squared, height and squared deviation of height with sex, education (low, medium, high), body mass index, spirometer type, study center as well as cell composition. The M_{base}-adjusted model explained 71.0% of the variance in the outcome. The M_{base}-adjusted model additionally adjusted for the Mediation-SI explained 71.9% of the FEV1 variance (total adjusted R^2 = 0.719) of which 3.2% of the variance was specifically explained by the SI variable. This was comparable to the variance explained by the M_{base}-adjusted model additionally adjusted for packyears and smoking status corresponding to the M_{smok} model (R^2 = 0.721, and with 3.3% of the variance specifically explained by the packyears variable). Model including both smoking adjustments (M_{smok} and additionally Mediation-SI) explained 72.4% of the FEV1 variance.

15.4. Figure S7: Association of Mediation-SI with FVC and comparison with association of packyears with FVC

Figure S7: Distribution and association* of Mediation-SI with FVC, with 95% confidence interval. (A) Boxplot of Mediation-SI (median: 0.3 and range: -1.7 to 5.2) in all participants of SAPALDIA. (B) Boxplot of packyears (median: 2.0 and range: 0 to 145.9) in all participants of SAPALDIA

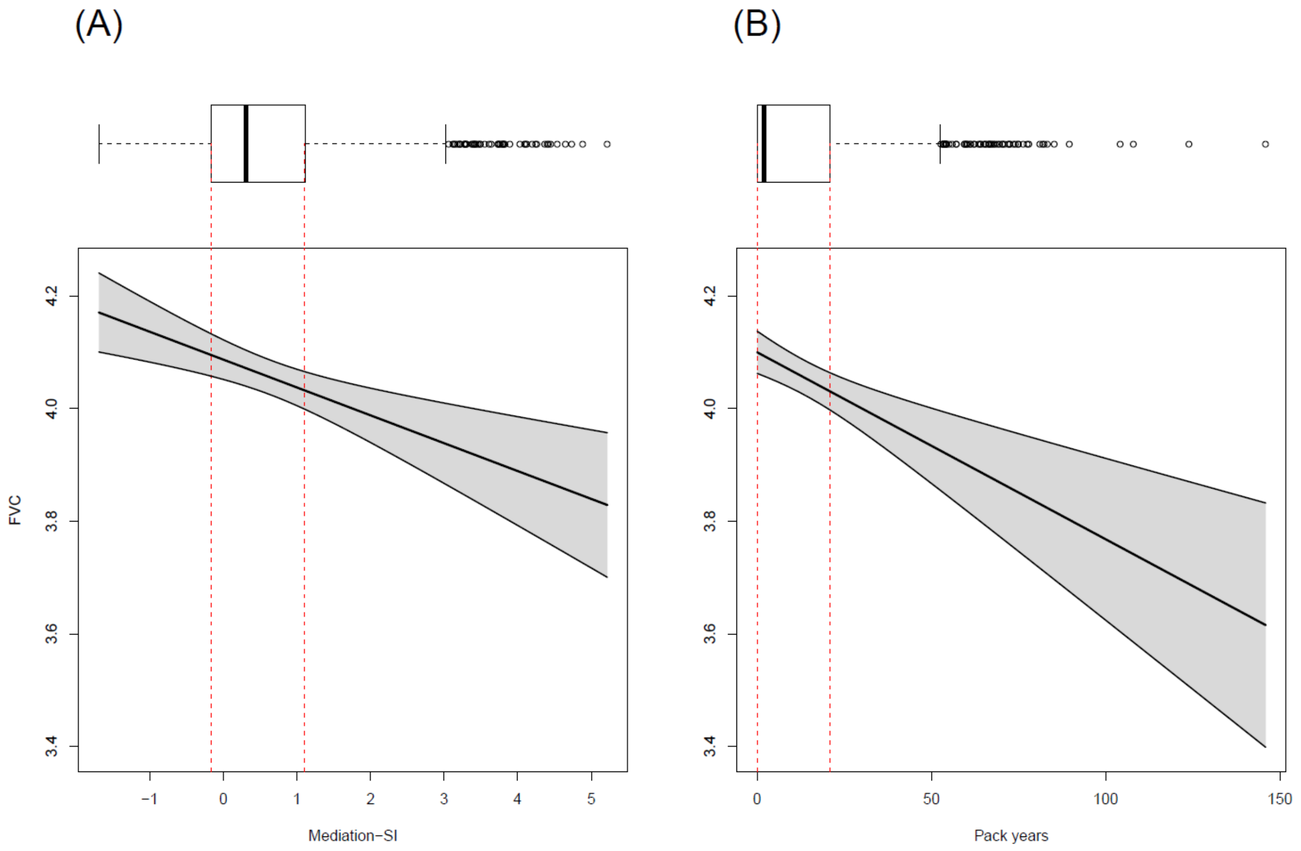

Footnote to Figure S7:

*Associations were adjusted for the base model (M_{base}): age, age squared, height, squared deviation from the mean of height, sex and interaction terms of age, age squared, height and squared deviation of height with sex, education (low, medium, high), body mass index, spirometer type, study center as well as cell composition. The M_{base}-adjusted model explained 78.8% of the variance in the outcome. The M_{base}-adjusted model additionally adjusted for the Mediation-SI explained 79.1% of the FVC variance (total adjusted R^2 = 0.791) of which 1.2% of the variance was specifically explained by the SI variable. This was comparable to the variance explained by the M_{base}-adjusted model additionally adjusted for packyears and smoking status corresponding to the M_{smok} model (adjusted R2 = 0.792, and with 0.5% of the variance specifically explained by the packyears variable). Model including both smoking adjustments (Msmok and additionally Mediation-SI) explained 79.3% of the FVC variance (total adjusted R^2 = 0.793).

15.5. Table S23: Mediation-SI association with FEV1 and with FVC by smoking strata

Table S23: Meta-analyses* of Mediation-SI on the cross-sectional association with lung function, FEV_1 (L) and FVC (L), time point 2, separately, and longitudinally predicting the change in lung function during follow-up, FEV_1 (ml/year) and FVC (ml/year), base model adjustment (M_{base}†), in all study participant, ever and never smokers.

	Cross-sectional meta-analysis at time point 2				Prediction on change in lung function			
	beta (SE)	P-value¶	Direction¶	P-value between study hetero-geneity	beta (SE)	P-value¶	Direction¶	P-value between study hetero-geneity
**FEV1**								
All	-0.0650 (0.0089)	2.32E-13	---	0.31	-0.0038 (0.0005)	9.74E-14	---	0.24
Ever smokers	-0.0861 (0.0116)	1.02E-13	---	0.34	-0.0037 (0.0007)	9.74E-08	---	0.22
Never smokers	-0.0241 (0.0283)	0.394	--+	0.055	-0.0047(0.0012)	1.15E-04	--+	0.12
**FVC**								
All	-0.0252 (0.0101)	0.0125	-+-	0.0066	-0.0034 (0.0007)	4.10E-07	---	0.021
Ever smokers	-0.0426 (0.0132)	0.0013	-+-	0.031	-0.0041 (0.0009)	5.68E-06	---	0.12
Never smokers	-0.0207 (0.0323)	0.522	-++	0.031	-0.0021 (0.0015)	0.151	---	0.14

Footnotes to table S23:

*Cohort-specific association results for Mediation-SI were meta-analysed. The 10 CpGs contributing Mediation-SI are shown in online supplement table S21). Note: DNAme predictors used were technical bias-adjusted, normalized residuals, thus effect size of the association (beta) are not directly comparable to elsewhere reported effect sizes using normalized %-methylation as predictor.

† Base model covariate adjustment (M_{base}): age, age squared, height, squared deviation from the mean of height, sex and interaction terms of age, age squared, height and squared deviation of height with sex, education (low, medium, high), body mass index, spirometer type, study center as well as cell composition. Prediction models were additionally adjusted for FEV_1/FVC at time point 1.

‡ P-value of meta-analysis: P<0.008 was considered statistically significant, Bonferroni correction for 6 tests per lung function outcome. Order of cohorts for direction of effects: ECRHS, NFBC1966, SAPALDIA.

Abbreviations: beta – coefficient of association; chr – chromosome; SE – standard error.

15.6. Figure S8: Mediation-SI association with FEV_1 in ever - and never smokers

<u>Figure S8:</u> Forest plots of cohort-specific results and meta-analyses of the association of the Mediation-SI with FEV_1 and change in FEV_1 in ever - and never smokers in the discovery cohorts. Associations run applying base model adjustment (M_{base}*).

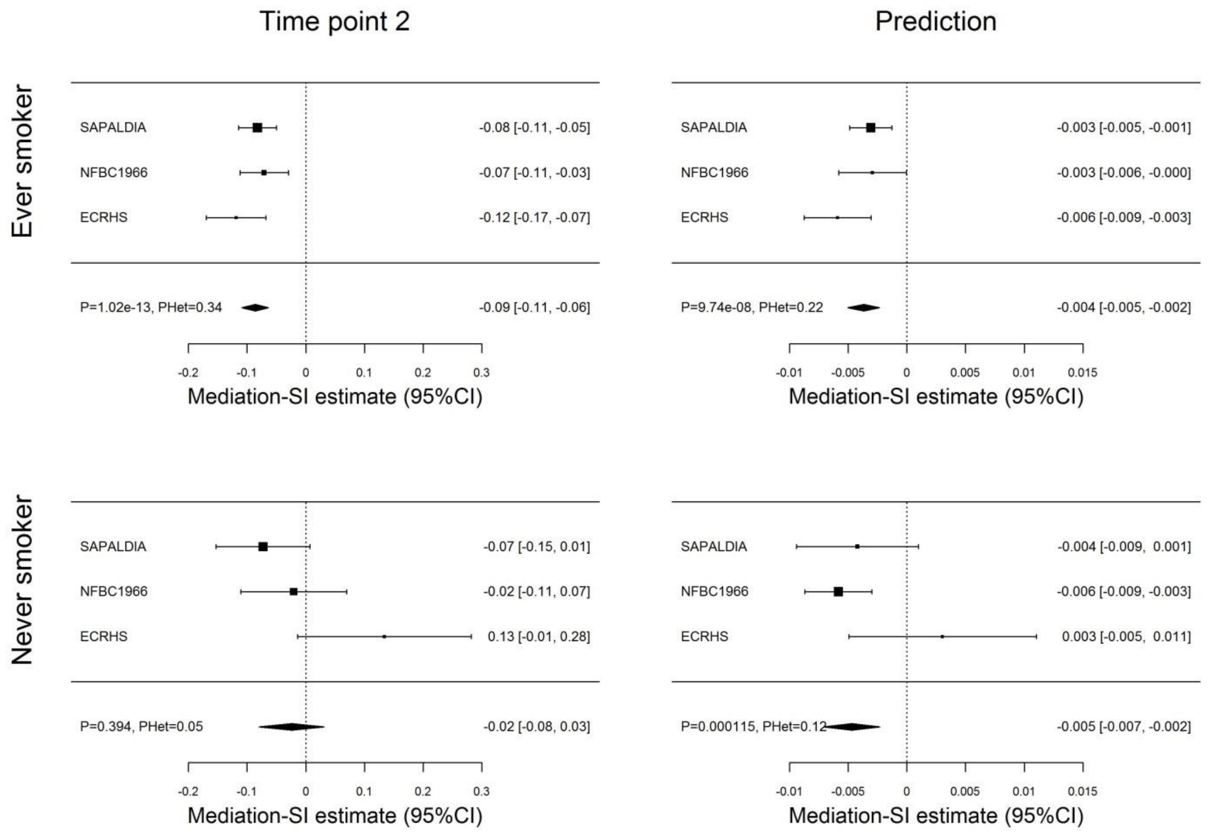

<u>Footnote to Figure S8:</u>

*Base model (M_{base}): EWAS were covariate adjusted for age, age squared, height, squared deviation from the mean of height, sex and interaction terms of age, age squared, height and squared deviation of height with sex, education (low, medium, high), body mass index, spirometer type, study center as well as cell composition. Prediction models were additionally adjusted for FEV_1 at time point 1.

16. Results: Scatter plots of percent methylation with lung function outcomes of 57 replicated CpGs

16.1. Figure S9: Scatter plots visualizing the relationship between percent methylation (β-value, X-axis) with FEV1/FVC (ratio, y-axis) in SAPALDIA at time point 2

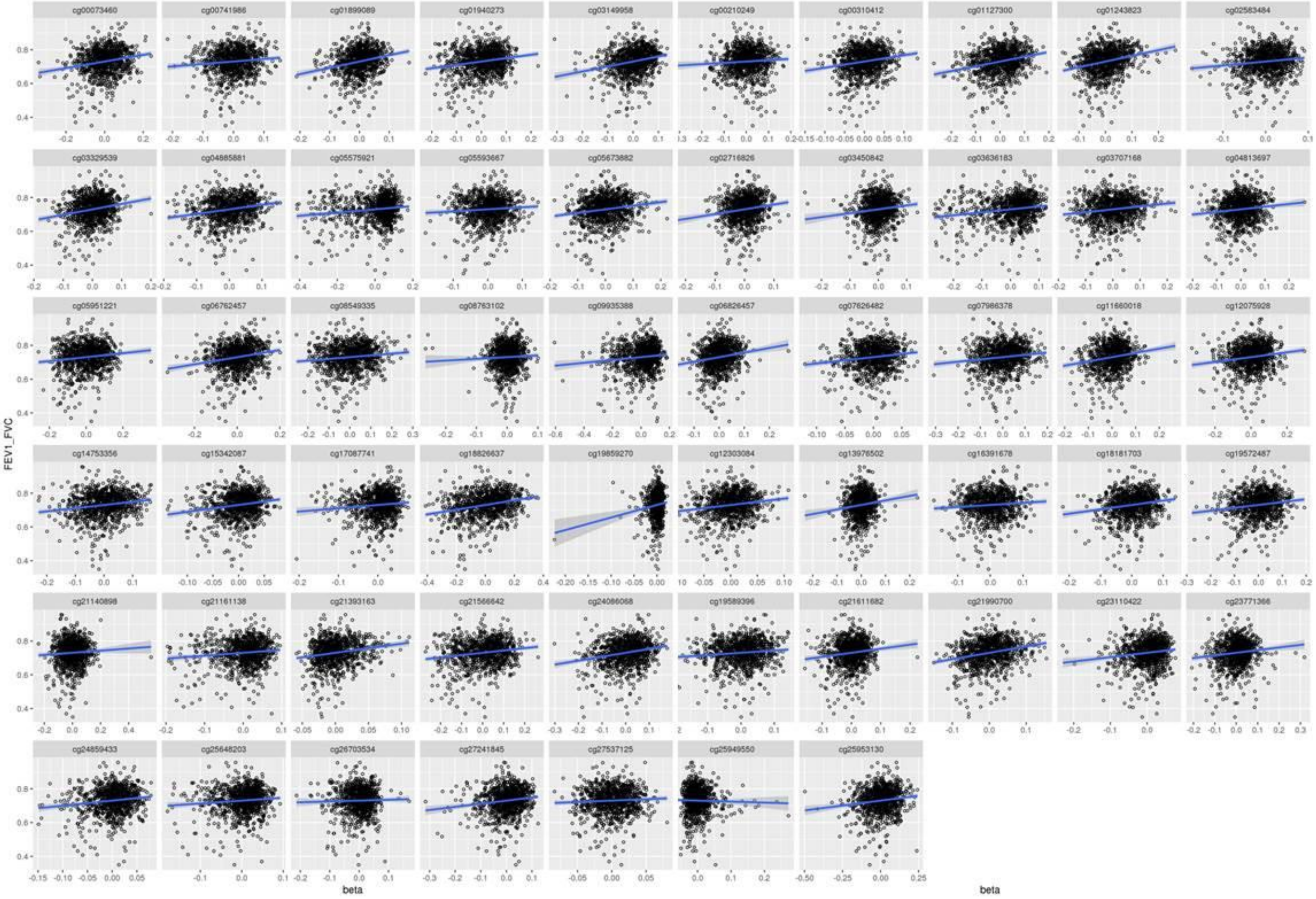

16.2. Figure S10: Scatter plots visualizing the relationship between percent methylation (β-value, X-axis) with FEV1 (L, y-axis) in SAPALDIA at time point 2

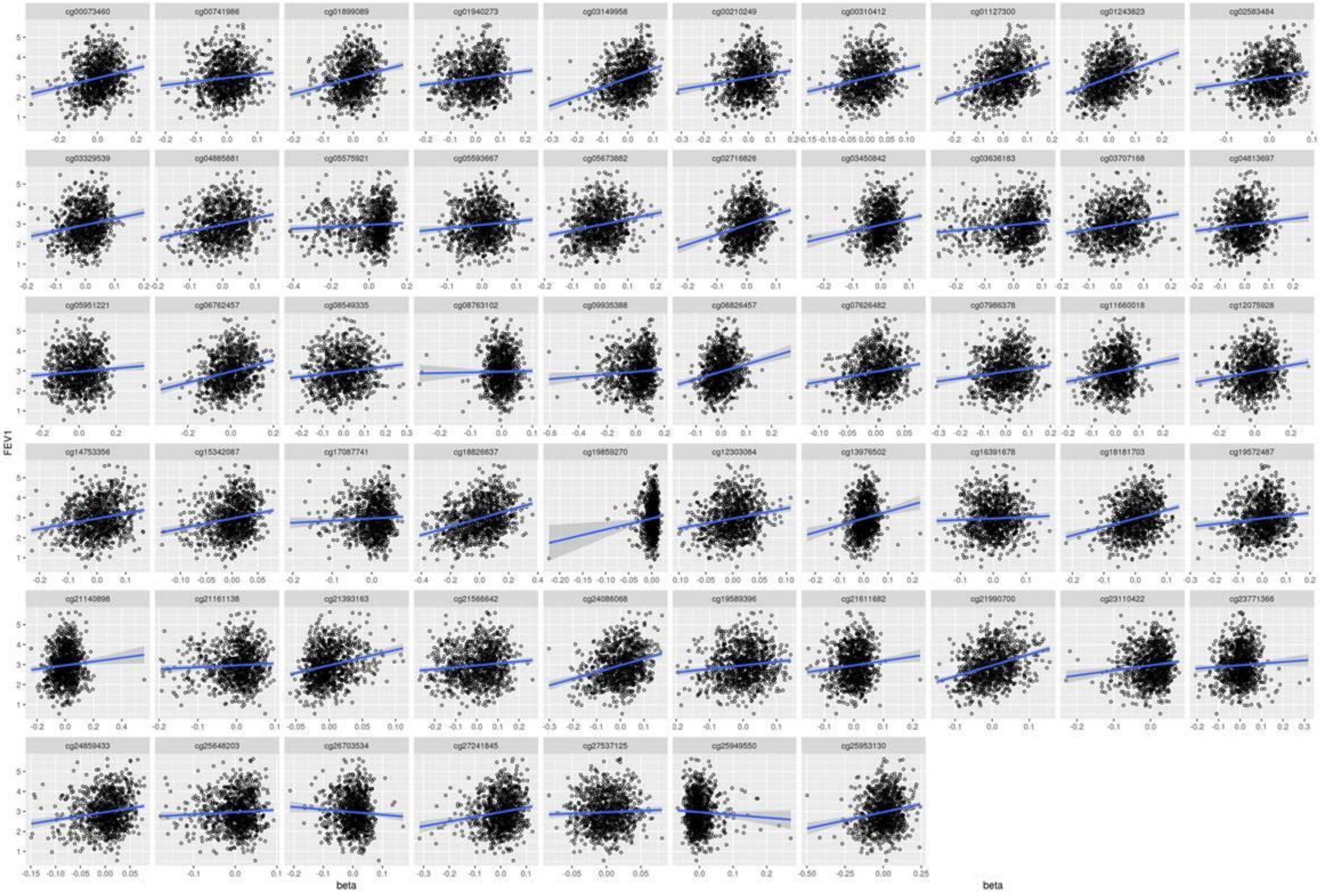

16.3. Figure S11: Scatter plots visualizing the relationship between percent absolute methylation (β-value, X-axis) with FVC (L, y-axis) in SAPALDIA at time point 2

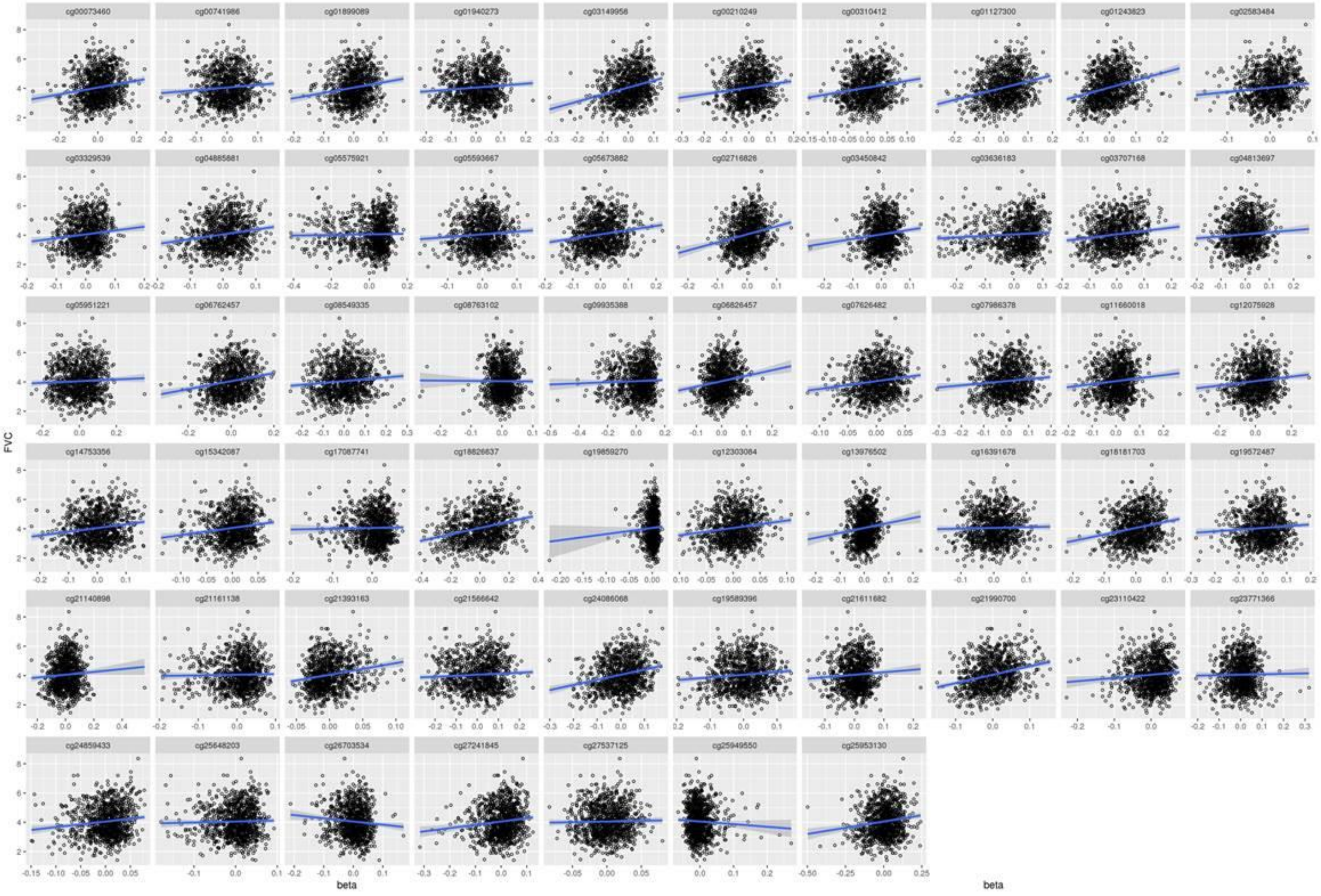

17. Results: Candidate Gene based DNAme smoking index (lung-function-gene-SI)

17.1. Table S24: List of CpGs contributing to lung-function-gene-SI

Table S24: List of CpGs contributing to the gene-based smoking index (lung-function-gene-SI) representing a subset of CpG markers previously identified to be associated with smoking* that were located in GWAS identified genes associated with lung function. Information on ranks of association of the selected CpGs in the discovery EWAS (M_{base}) with FEV_1 are presented, similar low ranks were observed for association with FEV_1/FVC or FVC.

	Locus	CpG ID	chr	position	smoking FDR P-value*	smoking direction of effects*	rank in EWAS on FEV1 in repeat cross-sectional meta-analysis (discovery)
1	ADAM19	cg08295410	5	156'990'663	4.70E-05	++++++++++++++-+	113170
2	ARMC2	cg25127315	6	109'169'227	0.0023	+++-++-++-+++-++	425861
3	C10orf11	cg23024158	10	78'011'952	3.00E-04	--+------+---+--	135262
4	CDC123	cg19576422	10	12'256'343	7.00E-04	---+-??------+--	378523
5	CFDP1	cg10121429	16	75'466'707	0.0036	-----+---+------	315711
6	GPR126	cg11176095	6	142'622'515	7.00E-04	++-+++++++++++++	146657
7	HDAC4	cg11550064	2	240'148'191	5.50E-07	++-+++++++++++++	82203
8	HTR4	cg07102705	5	148'033'896	3.00E-04	+++-+-+++++++-++	206521
9	LRP1	cg14621254	12	57'569'787	0.0165	---+-+-------+--	151255
10	MECOM	cg02556393	3	168'866'705	2.60E-20	--------------+-	12608
11	MFAP2	cg04236263	1	17'305'798	0.0099	---+-----+-+---+	142956
12	PPT2	cg06814287	6	32'120'584	1.60E-10	---+---+------+-	227036
13	RARB	cg27574595	3	25'583'274	0.0062	++-++++-++++++--	33899
14	RHOBTB3	cg02549492	5	95'066'568	0.003	+++++++++++-+-++	393163
15	TGFB2	cg07810039	1	218'524'558	0.0024	++--+-+-++++++++	260626
16	TLE3	cg10381071	15	70'391'035	3.60E-06	--+-------------	44658
17	TNS1	cg06320380	2	218'770'208	0.0011	++-++++-+++++-++	131281
18	ZNF323;ZKSCAN3	cg12212060	6	28'323'405	4.10E-06	---+-----+---+--	367753

Footnote to table S24
*Smoking CpGs defined on the reported FDR corrected P-value <0.05 for association reported with smoking status and reported direction of effects for association with smoking.[45]

Result Associations of lung-function-gene-SI with FEV_1, FEV_1/FVC and FVC:

A smoking index (SI) based on 18 candidate CpGs located in previously identified genes to determine lung function (lung-function-gene-SI) was constructed. The construction and associations of the SI were performed as for the ALEC custom SIs. Meta-analysis results of the lung-function-genes-SI in all participants and by smoking status strata are presented below (Table S25). The effect of the lung-function-genes-SI, combining the effects of gene candidate CpGs, was not as pronounced as that of the effect of the ALEC custom SI, combining the effects of the smoking-related CpGs. The strongest associations were observed in ever smokers for cross-sectional associations with FEV_1 and for prediction of change in FVC in ever smokers (β (SE) =-18.1ml/year (5.1), P=0.0004).

17.2. Table S25: Associations of lung-function-gene-SI with FEV_1, FEV_1/FVC and FVC

<u>**Table S25:**</u> Meta-analyses of lung function gene-based candidate smoking index (SI) based on a subset of CpG markers (18 CpG markers, Table S24) previously identified to be associated with smoking [45] were located in GWAS identified genes associated with lung function. Associations* on the cross-sectional association with FEV_1 (L), FEV_1/FVC (%) and FVC (L) at, time point 2 and on the prediction of the change in lung function during follow-up, FEV_1 (ml/year), FEV_1/FVC (%/year) and FVC (ml/year), base model adjustment (M_{base}†), in all study participant, ever and never smokers.

	Cross-sectional meta-analysis at time point 2				Prediction of SI on change in lung function meta-analysis			
	beta (SE)	P-value	Direction	P-value (het)	beta (SE)	P-value	Direction	P-value (het)
**FEV1**								
All	-0.11 (0.038)	**0.0038**	---	0.841	-7.1 (2.7)	0.0078	---	0.605
Ever smokers	-0.196 (0.053)	**0.0002**	---	0.173	10.1 (3.7)	0.0058	---	0.095
Never smokers	0.048 (0.059)	0.4178	+--	0.045	0.3 (4.3)	0.9436	++-	0.848
**FEV1/FVC**								
All	-1.04 (0.54)	0.0560	---	0.656	-0.04 (0.05)	0.3568	-+-	0.874
Ever smokers	-0.97 (0.75)	0.1949	-+-	0.363	-0.01 (0.06)	0.8188	++-	0.837
Never smokers	0.13 (1.09)	0.9047	+--	0.925	-0.02 (0.08)	0.8316	--+	0.830
**FVC**								
All	-0.098 (0.043)	0.0233	---	0.795	-11.1 (3.6)	**0.0021**	---	0.511
Ever smokers	-0.203 (0.059)	**0.0006**	---	0.215	-18.1 (5.1)	**0.0004**	---	0.019
Never smokers	0.002 (0.013)	0.8835	+--	0.020	0.6 (5.7)	0.9223	++-	0.433

<u>*Footnotes to table 25:*</u>

*Cohort-specific association results for lung-function-gene -SI were meta-analysed.

† Base model covariate adjustment (M_{base}): age, age squared, height, squared deviation from the mean of height, sex and interaction terms of age, age squared, height and squared deviation of height with sex, education (low, medium, high), body mass index, spirometer type, study center as well as cell composition. Prediction models were additionally adjusted for FEV_1/FVC at time point 1.

‡ P-value of meta-analysis: P<0.008 was considered statistically significant, Bonferroni correction for 6 tests per lung function outcome. Order of cohorts for direction of effects: ECRHS, NFBC1966, SAPALDIA.

Abbreviations: beta – coefficient of association; chr – chromosome; SE – standard error; P-value (het) stands for P-value between study heterogeneity.

18. Acknowledgments & Funding of cohorts:

18.1. Discovery cohort: ECRHS - European Community Respiratory Health Survey

Acknowledgments

The authors would like to thank the participants, field workers and researchers who have participated in the ECRHS study for their time and cooperation.

Funding

This work was supported by a contract from the European Commission (018996), Fondo de Investigación Sanitaria (91/0016-060-05/E, 92/0319, 93/0393, 97/0035-01, 99/0034-01 and 99/0034-02), Hospital General de Albacete, Hospital General Ramón Jiménez, Consejería de Sanidad del Principado de Asturias, CIRIT (1997SGR 00079, 1999SGR 00241), and Servicio Andaluz de Salud, SEPAR, Public Health Service (R01 HL62633-01), RCESP (C03/09), Red RESPIRA (C03/011), Basque Health Department, Swiss National Science Foundation, Swiss Federal Office for Education and Science, Swiss National Accident Insurance Fund (SUVA), GSF-National Research Centre for Environment and Health, Deutsche Forschungsgemeinschaft (DFG) (FR 1526/1-1, MA 711/4-1), Programme Hospitalier de Recherche Clinique-DRC de Grenoble 2000 no. 2610, Ministry of Health, Direction de la Recherche Clinique, Ministere de l’Emploi et de la Solidarite, Direction Generale de la Sante, CHU de Grenoble, Comite des Maladies Respiratoires de l’Isere. UCB-Pharma (France), Aventis (France), Glaxo France. Estonian Science Foundation, and Asthma UK (formerly known as National Asthma Campaign UK).

18.2. Discovery cohort: NFBC1966 - Northern Finland Birth Cohort 1966

Acknowledgments

We thank the late Professor Paula Rantakallio (launch of NFBC1966), and Ms Outi Tornwall and Ms Minttu Jussila (DNA biobanking). The authors would like to acknowledge the contribution of the late Academian of Science Leena Peltonen.

Funding

The NFBC resource has been supported by grants from the Academy of Finland (project grants 104781, 120315, 129269, 1114194, 24300796, Center of Excellence in Complex Disease Genetics and SALVE), University Hospital Oulu, Biocenter, University of Oulu, Finland (75617), NHLBI grant 5R01HL087679-02 (1RL1MH083268-01), NIH/NIMH (5R01MH63706:02), ENGAGE project and grant agreement HEALTH-F4-2007-201413, EU FP7 EurHEALTHAgeing -277849, the Medical Research Council, UK (G0500539, G0600705, G1002319, PrevMetSyn/SALVE) and the MRC, Centenary Early Career Award. H2020 DynaHEALTH (European Union's Horizon 2020 research and innovation programme under grant agreement No 633595); Exposomic, Genomic and Epigenomic Approach to Prediction of Metabolic and Cardiorespiratory function and Ill-Health (EGEA), Academy of Finland, Grant No 285547; ALEC Study (funded by the European Union's Horizon 2020 Research and Innovation programme under grant agreement No. 633212); H2020 / Marie Skłodowska-Curie Actions, CAPICE (Marie Curie Grant agreement Number 721567); National Public Health Institute, Biomedicum Helsinki, Finland.

18.3. Discovery cohort: SAPALDIA - Swiss Study on Air Pollution Heart and Lung Disease in Adults

Acknowledgments

The study could not have been done without the help of the study participants, technical and administrative support and the medical teams and field workers at the local study sites.

Study directorate: NM Probst-Hensch (PI; e/g); T Rochat (p), C Schindler (s), N Künzli (e/exp), JM Gaspoz (c)

Scientific team: JC Barthélémy (c), W Berger (g), R Bettschart (p), A Bircher (a), C Brombach (n), PO Bridevaux (p), L Burdet (p), Felber Dietrich D (e), M Frey (p), U Frey (pd), MW Gerbase (p), D Gold (e), E de Groot (c), W Karrer (p), F Kronenberg (g), B Martin (pa), A Mehta (e), D Miedinger (o), M Pons (p), F Roche (c), T Rothe (p), P Schmid-Grendelmeyer (a), D Stolz (p), A Schmidt-Trucksäss (pa), J Schwartz (e), A Turk (p), A von Eckardstein (cc), E Zemp Stutz (e).

Scientific team at coordinating centers: M Adam (e), I Aguilera (exp), S Brunner (s), D Carballo (c), S Caviezel (pa), I Curjuric (e), A Di Pascale (s), J Dratva (e), R Ducret (s), E Dupuis Lozeron (s), M Eeftens (exp), I Eze (e), E Fischer (g), M Foraster (e), M Germond (s), L Grize (s), S Hansen (e), A Hensel (s), M Imboden (g), A Ineichen (exp), A Jeong (g), D Keidel (s), A Kumar (g), N Maire (s), A Mehta (e), R Meier (exp), E Schaffner (s), T Schikowski (e), M Tsai (exp)

(a) allergology, (c) cardiology, (cc) clinical chemistry, (e) epidemiology, (exp) exposure, (g) genetic and molecular biology, (m) meteorology, (n) nutrition, (o) occupational health, (p) pneumology, (pa) physical activity, (pd) pediatrics, (s) statistics

Funding

The Swiss National Science Foundation (grants no 33CS30-148470/1&2, 33CSCO-134276/1, 33CSCO-108796, 324730_135673, 3247BO-104283, 3247BO-104288, 3247BO-104284, 3247-065896, 3100-059302, 3200-052720, 3200-042532, 4026-028099, PMPDP3_129021/1, PMPDP3_141671/1), the Federal Office for the Environment, the Federal Office of Public Health, the Federal Office of Roads and Transport, the canton's government of Aargau, Basel-Stadt, Basel-Land, Geneva, Luzern, Ticino, Valais, and Zürich, the Swiss Lung League, the canton's Lung League of Basel Stadt/ Basel Landschaft, Geneva, Ticino, Valais, Graubünden and Zurich, Stiftung ehemals Bündner Heilstätten, SUVA, Freiwillige Akademische Gesellschaft, UBS Wealth Foundation, Talecris Biotherapeutics GmbH, Abbott Diagnostics, European Commission 018996 (GABRIEL), Wellcome Trust WT 084703MA, Exposomics EC FP7 grant(Grant agreement No: 308610).

18.4. Replication cohort: ALSPAC - Avon Longitudinal Study of Parents and Children

Acknowledgments

We are extremely grateful to all the families who took part in this study, the midwives for their help in recruiting them, and the whole ALSPAC team, which includes interviewers, computer and laboratory technicians, clerical workers, research scientists, volunteers, managers, receptionists and nurses.

Funding

The UK Medical Research Council and Wellcome (Grant ref: 102215/2/13/2) and the University of Bristol provide core support for ALSPAC. The Accessible Resource for Integrated Epigenomics Studies (ARIES) which generated large scale methylation data was funded by the UK Biotechnology and Biological Sciences Research Council (BB/I025751/1 and BB/I025263/1). Additional epigenetic profiling on the ALSPAC cohort was supported by the UK Medical Research Council Integrative Epidemiology Unit and the University of Bristol (MC_UU_12013_1, MC_UU_12013_2, MC_UU_12013_5 and MC_UU_12013_8), the Wellcome Trust (WT088806) and the United States National Institute of Diabetes and Digestive and Kidney Diseases (R01 DK10324).

18.5. Replication cohort: FTC - Finnish Twin Cohort

Acknowledgments

We thank the participants for their invaluable contributions to the study. The Obesity Research Unit team and the staff at the Finnish Twin Cohort Study are acknowledged for their help in the collection of the data. Jaakko Kaprio, University of Helsinki, and Richard J. Rose, Indiana University, are thanked for their contributions to FinnTwin16 cohort

Funding

The FTC data collection and analyses were supported by grants from the Academy of Finland (grants: 297908, 251316), University of Helsinki (grant: 490139), and Sigrid Juselius Foundation received by MO, Academy of Finland Centre of Excellence in Research on Mitochondria, Metabolism and Disease (grant number: 272376), Academy of Finland grants: 314383, 266286), Finnish Medical Foundation, Gyllenberg Foundation, Finnish Foundation for Cardiovascular

Research, Novo Nordisk Foundation, University of Helsinki, Helsinki University Hospital, Government Research Funds, Finnish Diabetes Research Foundation received by KHP, Academy of Finland Centre of Excellence in Complex Disease Genetics (grant numbers: 129680 and 213506), National Institute of Alcohol Abuse and Alcoholism, (grant numbers: AA-09203 and AA-12502),and Academy of Finland, (grant numbers: 141054, 263278, 264146, and 265240) received by Jaakko Kaprio.

18.6. Replication cohort: IOWBC – Isle Of Wight Birth Cohort

Acknowledgments

The authors gratefully acknowledge the cooperation of the children and parents who have participated in this study.

Funding

Recruitment and initial assessment for the first 4 years of age was supported by the Isle of Wight Health Authority. The 10-year follow- up of this study was funded by the National Asthma Campaign, UK (Grant No 364) and the 18-year follow-up by NIH/ NHLBI R01 HL082925-01. The 21 year assessment was funded by Medical Research Council, UK (G23369). The 26 years assessment was supported by The David Hide Asthma & Allergy Research Trust. Measurements of DNA-methylation and their analyses were funded by grants from the National Institute of Health (R01 AI091905, R01 AI121226, R01 HL132321).

18.7. Replication cohort: KORA - Cooperative Health Research in the Augsburg Region Study

Acknowledgments

We are very grateful and thank all participants involved in the KORA surveys for their participation and cooperation. We also thank the clinical staff and scientific teams of the study centre in Augsburg very much for their excellent care of all participants. We further thank Nadine Lindemann, Franziska Scharl and Sonja Kunze for technical support.

Funding

The KORA study was initiated and financed by the Helmholtz Zentrum München – German Research Center for Environmental Health, which was funded by the German Federal Ministry of Education and Research (BMBF) and by the State of Bavaria. Furthermore, KORA research was supported by the Munich Center of Health Sciences (MC-Health), Ludwig-Maximilians-Universität, as part of LMUinnovativ. This work was also supported by the Comprehensive Pneumology Center Munich (CPC-M) as member of the German Center for Lung Research and the Competence Network Asthma and COPD (ASCONET), network COSYCONET (subproject 2, BMBF FKZ 01GI0882) funded by the German Federal Ministry of Education and Research (BMBF).

18.8. Replication cohort: LBC1936 - Lothian Birth Cohort 1936

Acknowledgments

We thank the cohort participants and team members who contributed to these studies. We gratefully acknowledge the contribution of co-author Professor John M. Starr, who died prior to the publication of this manuscript.

Funding

Phenotype collection in the LBC1936 was supported by Age UK (The Disconnected Mind project). Methylation typing was supported by Centre for Cognitive Ageing and Cognitive Epidemiology (Pilot Fund award), Age UK, The Wellcome Trust Institutional Strategic Support Fund, The University of Edinburgh, and The University of Queensland. The work was undertaken by The University of Edinburgh Centre for Cognitive Ageing and Cognitive Epidemiology, part of the cross council Lifelong Health and Wellbeing Initiative (MR/K026992/1). Funding from the BBSRC and MRC is gratefully acknowledged.

18.9. Replication cohort: LifeLines Cohort

Acknowledgments

The authors wish to acknowledge the services of the LifeLines Cohort Study, the contributing research centres delivering data to LifeLines and the participating general practitioners, and all the study participants.

Funding

This work was supported by consortium grant number 4.1.13.007 of the Lung foundation Netherlands and a personal grant from the UMCG to H.M. Boezen. The LifeLines initiative has been made possible by funds from FES (Fonds Economische Structuurversterking), SNN (Samenwerkingsverband Noord Nederland) and REP (Ruimtelijk Economisch Programma).

18.10. Replication cohort: NSPHS - Northern Sweden Population Health Study

Acknowledgments

DNA methylation analyses were performed by the SNP & SEQ Technology Platform in Uppsala, which is supported by Uppsala University, Uppsala University Hospital, Science for Life Laboratory (SciLifeLab)—Uppsala and the Swedish Research Council (Contracts 80576801 and 70374401). The computations were performed on resources provided by SNIC through Uppsala Multidisciplinary Center for Advanced Computational Science (UPPMAX) under projects b2012153.

Funding

The NSPHS study was funded by the Swedish Medical Research Council (Project Number K2007-66X-20270-01-3) and the Foundation for Strategic Research. The DNA Methylation study in NSPHS has been funded by Swedish Medical Research Council (Project Number 2011-2354) and the Göran Gustafssons Foundation. This work has also been supported by the Swedish Society for Medical Research (SSMF), the Kjell och Märta Beijers Foundation , Swedish Medical Research Council (Project Number 2015-03327).

19. References

1. Burney PG, Luczynska C, Chinn S, Jarvis D. The European Community Respiratory Health Survey. *The European respiratory journal : official journal of the European Society for Clinical Respiratory Physiology* 1994: 7(5): 954-960.
2. Rantakallio P. The longitudinal study of the northern Finland birth cohort of 1966. *Paediatr Perinat Epidemiol* 1988: 2(1): 59-88.
3. Sovio U, Bennett AJ, Millwood IY, Molitor J, O'Reilly PF, Timpson NJ, Kaakinen M, Laitinen J, Haukka J, Pillas D, Tzoulaki I, Molitor J, Hoggart C, Coin LJ, Whittaker J, Pouta A, Hartikainen AL, Freimer NB, Widen E, Peltonen L, Elliott P, McCarthy MI, Jarvelin MR. Genetic determinants of height growth assessed longitudinally from infancy to adulthood in the northern Finland birth cohort 1966. *PLoS Genet* 2009: 5(3): e1000409.
4. Lehne B, Drong AW, Loh M, Zhang W, Scott WR, Tan ST, Afzal U, Scott J, Jarvelin MR, Elliott P, McCarthy MI, Kooner JS, Chambers JC. A coherent approach for analysis of the Illumina HumanMethylation450 BeadChip improves data quality and performance in epigenome-wide association studies. *Genome Biol* 2015: 16: 37.
5. Martin BW, Ackermann-Liebrich U, Leuenberger P, Kunzli N, Stutz EZ, Keller R, Zellweger JP, Wuthrich B, Monn C, Blaser K, Bolognini G, Bongard JP, Brandli O, Braun P, Defila C, Domenighetti G, Grize L, Karrer W, Keller-Wossidlo H, Medici TC, Peeters A, Perruchoud AP, Schindler C, Schoeni MH, Villiger B, et al. SAPALDIA: methods and participation in the cross-sectional part of the Swiss Study on Air Pollution and Lung Diseases in Adults. *Soz Praventivmed* 1997: 42(2): 67-84.
6. Ackermann-Liebrich U, Kuna-Dibbert B, Probst-Hensch N, Schindler C, Felber Dietrich D, Zemp Stutz E, Bayer-Oglesby L, Baum F, Brändli O, Brutsche M, Downs S, Keidel D, Gerbase M, Imboden M, Keller R, Knöpfli B, Künzli N, Nicod L, Pons M, Staedele P, Tschopp JM, Zellweger JP, Leuenberger P, team S. Follow-up of the Swiss Cohort Study on Air Pollution and Lung Diseases in Adults (SAPALDIA 2) 1991-2003: methods and characterization of participants. *Soz Praventiv Med* 2005: 50: 245-263.
7. Fraser A, Macdonald-Wallis C, Tilling K, Boyd A, Golding J, Davey Smith G, Henderson J, Macleod J, Molloy L, Ness A, Ring S, Nelson SM, Lawlor DA. Cohort Profile: the Avon Longitudinal Study of Parents and Children: ALSPAC mothers cohort. *International journal of epidemiology* 2013: 42(1): 97-110.
8. Boyd A, Golding J, Macleod J, Lawlor DA, Fraser A, Henderson J, Molloy L, Ness A, Ring S, Davey Smith G. Cohort Profile: the 'children of the 90s'--the index offspring of the Avon Longitudinal Study of Parents and Children. *International journal of epidemiology* 2013: 42(1): 111-127.
9. Relton CL, Gaunt T, McArdle W, Ho K, Duggirala A, Shihab H, Woodward G, Lyttleton O, Evans DM, Reik W, Paul YL, Ficz G, Ozanne SE, Wipat A, Flanagan K, Lister A, Heijmans BT, Ring SM, Davey Smith G. Data Resource Profile: Accessible Resource for Integrated Epigenomic Studies (ARIES). *International journal of epidemiology* 2015: 44(4): 1181-1190.
10. Aryee MJ, Jaffe AE, Corrada-Bravo H, Ladd-Acosta C, Feinberg AP, Hansen KD, Irizarry RA. Minfi: a flexible and comprehensive Bioconductor package for the analysis of Infinium DNA methylation microarrays. *Bioinformatics* 2014: 30(10): 1363-1369.
11. Houseman EA, Accomando WP, Koestler DC, Christensen BC, Marsit CJ, Nelson HH, Wiencke JK, Kelsey KT. DNA methylation arrays as surrogate measures of cell mixture distribution. *BMC Bioinformatics* 2012: 13: 86.
12. Kotecha SJ, Watkins WJ, Heron J, Henderson J, Dunstan FD, Kotecha S. Spirometric lung function in school-age children: effect of intrauterine growth retardation and catch-up growth. *American journal of respiratory and critical care medicine* 2010: 181(9): 969-974.
13. Kaprio J. The Finnish Twin Cohort Study: an update. *Twin Res Hum Genet* 2013: 16(1): 157-162.
14. Kaprio J. Twin studies in Finland 2006. *Twin Res Hum Genet* 2006: 9(6): 772-777.
15. Miller MR, Hankinson J, Brusasco V, Burgos F, Casaburi R, Coates A, Crapo R, Enright P, van der Grinten CP, Gustafsson P, Jensen R, Johnson DC, MacIntyre N, McKay R, Navajas D, Pedersen OF,

Pellegrino R, Viegi G, Wanger J, Force AET. Standardisation of spirometry. *The European respiratory journal : official journal of the European Society for Clinical Respiratory Physiology* 2005: 26(2): 319-338.
16. Quanjer PH, Stanojevic S, Cole TJ, Baur X, Hall GL, Culver BH, Enright PL, Hankinson JL, Ip MS, Zheng J, Stocks J, Initiative ERSGLF. Multi-ethnic reference values for spirometry for the 3-95-yr age range: the global lung function 2012 equations. *The European respiratory journal : official journal of the European Society for Clinical Respiratory Physiology* 2012: 40(6): 1324-1343.
17. Ollikainen M, Ismail K, Gervin K, Kyllonen A, Hakkarainen A, Lundbom J, Jarvinen EA, Harris JR, Lundbom N, Rissanen A, Lyle R, Pietilainen KH, Kaprio J. Genome-wide blood DNA methylation alterations at regulatory elements and heterochromatic regions in monozygotic twins discordant for obesity and liver fat. *Clin Epigenetics* 2015: 7: 39.
18. Arshad SH, Holloway JW, Karmaus W, Zhang H, Ewart S, Mansfield L, Matthews S, Hodgekiss C, Roberts G, Kurukulaaratchy R. Cohort Profile: The Isle Of Wight Whole Population Birth Cohort (IOWBC). *International journal of epidemiology* 2018.
19. Mukherjee N, Lockett GA, Merid SK, Melen E, Pershagen G, Holloway JW, Arshad SH, Ewart S, Zhang H, Karmaus W. DNA methylation and genetic polymorphisms of the Leptin gene interact to influence lung function outcomes and asthma at 18 years of age. *Int J Mol Epidemiol Genet* 2016: 7(1): 1-17.
20. Holle R, Happich M, Lowel H, Wichmann HE, Group MKS. KORA--a research platform for population based health research. *Gesundheitswesen* 2005: 67 Suppl 1: S19-25.
21. Wichmann HE, Gieger C, Illig T, Group MKS. KORA-gen--resource for population genetics, controls and a broad spectrum of disease phenotypes. *Gesundheitswesen* 2005: 67 Suppl 1: S26-30.
22. Lowel H, Meisinger C, Heier M, Hormann A. The population-based acute myocardial infarction (AMI) registry of the MONICA/KORA study region of Augsburg. *Gesundheitswesen* 2005: 67 Suppl 1: S31-37.
23. Zeilinger S, Kuhnel B, Klopp N, Baurecht H, Kleinschmidt A, Gieger C, Weidinger S, Lattka E, Adamski J, Peters A, Strauch K, Waldenberger M, Illig T. Tobacco smoking leads to extensive genome-wide changes in DNA methylation. *PloS one* 2013: 8(5): e63812.
24. Panni T, Mehta AJ, Schwartz JD, Baccarelli AA, Just AC, Wolf K, Wahl S, Cyrys J, Kunze S, Strauch K, Waldenberger M, Peters A. Genome-Wide Analysis of DNA Methylation and Fine Particulate Matter Air Pollution in Three Study Populations: KORA F3, KORA F4, and the Normative Aging Study. *Environmental health perspectives* 2016: 124(7): 983-990.
25. Deary IJ, Gow AJ, Taylor MD, Corley J, Brett C, Wilson V, Campbell H, Whalley LJ, Visscher PM, Porteous DJ, Starr JM. The Lothian Birth Cohort 1936: a study to examine influences on cognitive ageing from age 11 to age 70 and beyond. *BMC Geriatr* 2007: 7: 28.
26. Deary IJ, Gow AJ, Pattie A, Starr JM. Cohort profile: the Lothian Birth Cohorts of 1921 and 1936. *International journal of epidemiology* 2012: 41(6): 1576-1584.
27. Taylor AM, Pattie A, Deary IJ. Cohort Profile Update: The Lothian Birth Cohorts of 1921 and 1936. *International journal of epidemiology* 2018.
28. Shah S, McRae AF, Marioni RE, Harris SE, Gibson J, Henders AK, Redmond P, Cox SR, Pattie A, Corley J, Murphy L, Martin NG, Montgomery GW, Starr JM, Wray NR, Deary IJ, Visscher PM. Genetic and environmental exposures constrain epigenetic drift over the human life course. *Genome Res* 2014: 24(11): 1725-1733.
29. Scholtens S, Smidt N, Swertz MA, Bakker SJ, Dotinga A, Vonk JM, van Dijk F, van Zon SK, Wijmenga C, Wolffenbuttel BH, Stolk RP. Cohort Profile: LifeLines, a three-generation cohort study and biobank. *International journal of epidemiology* 2015: 44(4): 1172-1180.
30. Stolk RP, Rosmalen JG, Postma DS, de Boer RA, Navis G, Slaets JP, Ormel J, Wolffenbuttel BH. Universal risk factors for multifactorial diseases: LifeLines: a three-generation population-based study. *Eur J Epidemiol* 2008: 23(1): 67-74.

31. Igl W, Johansson A, Gyllensten U. The Northern Swedish Population Health Study (NSPHS)--a paradigmatic study in a rural population combining community health and basic research. *Rural Remote Health* 2010: 10(2): 1363.
32. Ahsan M, Ek WE, Rask-Andersen M, Karlsson T, Lind-Thomsen A, Enroth S, Gyllensten U, Johansson A. The relative contribution of DNA methylation and genetic variants on protein biomarkers for human diseases. *PLoS genetics* 2017: 13(9): e1007005.
33. Assenov Y, Muller F, Lutsik P, Walter J, Lengauer T, Bock C. Comprehensive analysis of DNA methylation data with RnBeads. *Nat Methods* 2014: 11(11): 1138-1140.
34. Teschendorff AE, Marabita F, Lechner M, Bartlett T, Tegner J, Gomez-Cabrero D, Beck S. A beta-mixture quantile normalization method for correcting probe design bias in Illumina Infinium 450 k DNA methylation data. *Bioinformatics* 2013: 29(2): 189-196.
35. Touleimat N, Tost J. Complete pipeline for Infinium((R)) Human Methylation 450K BeadChip data processing using subset quantile normalization for accurate DNA methylation estimation. *Epigenomics* 2012: 4(3): 325-341.
36. European Community Respiratory Health Survey IISC. The European Community Respiratory Health Survey II. *The European respiratory journal : official journal of the European Society for Clinical Respiratory Physiology* 2002: 20(5): 1071-1079.
37. Jarvis D, Newson R, Janson C, Corsico A, Heinrich J, Anto JM, Abramson MJ, Kirsten AM, Zock JP, Bono R, Demoly P, Leynaert B, Raherison C, Pin I, Gislason T, Jogi R, Schlunssen V, Svanes C, Watkins J, Weyler J, Pereira-Vega A, Urrutia I, Gullon JA, Forsberg B, Probst-Hensch N, Boezen HM, Martinez-Moratalla Rovira J, Accordini S, de Marco R, Burney P. Prevalence of asthma-like symptoms with ageing. *Thorax* 2018: 73(1): 37-48.
38. Bridevaux PO, Dupuis-Lozeron E, Schindler C, Keidel D, Gerbase MW, Probst-Hensch NM, Bettschart R, Burdet L, Pons M, Rothe T, Turk A, Stolz D, Tschopp JM, Kuenzli N, Rochat T. Spirometer Replacement and Serial Lung Function Measurements in Population Studies: Results From the SAPALDIA Study. *American journal of epidemiology* 2015: 181(10): 752-761.
39. Kunzli N, Kuna-Dibbert B, Keidel D, Keller R, Brändli O, Schindler C, Schweinzer K, Leuenberger P, Ackermann-Liebrich U, team S. Longitudinal validity of spirometers - a challenge in lung function follow-up studies. *Swiss Medical Weekly* 2005: 135(33-34): 503-508.
40. Kunzli N, Ackermann-Liebrich U, Keller R, Perruchoud AP, Schindler C. Variability of FVC and FEV1 due to technician, team, device and subject in an eight centre study: three quality control studies in SAPALDIA. Swiss Study on Air Pollution and Lung Disease in Adults. *The European respiratory journal : official journal of the European Society for Clinical Respiratory Physiology* 1995: 8(3): 371-376.
41. Lampi J, Koskela H, Hartikainen AL, Ramasamy A, Couto Alves A, Jarvelin MR, Pekkanen J. Farm environment during infancy and lung function at the age of 31: a prospective birth cohort study in Finland. *BMJ Open* 2015: 5(7): e007350.
42. Karrasch S, Flexeder C, Behr J, Holle R, Huber RM, Jorres RA, Nowak D, Peters A, Wichmann HE, Heinrich J, Schulz H, Group KS. Spirometric reference values for advanced age from a South german population. *Respiration* 2013: 85(3): 210-219.
43. Mustelin L, Latvala A, Pietilainen KH, Piirila P, Sovijarvi AR, Kujala UM, Rissanen A, Kaprio J. Associations between sports participation, cardiorespiratory fitness, and adiposity in young adult twins. *J Appl Physiol (1985)* 2011: 110(3): 681-686.
44. Teschendorff AE, Yang Z, Wong A, Pipinikas CP, Jiao Y, Jones A, Anjum S, Hardy R, Salvesen HB, Thirlwell C, Janes SM, Kuh D, Widschwendter M. Correlation of Smoking-Associated DNA Methylation Changes in Buccal Cells With DNA Methylation Changes in Epithelial Cancer. *JAMA Oncol* 2015: 1(4): 476-485.
45. Joehanes R, Just AC, Marioni RE, Pilling LC, Reynolds LM, Mandaviya PR, Guan W, Xu T, Elks CE, Aslibekyan S, Moreno-Macias H, Smith JA, Brody JA, Dhingra R, Yousefi P, Pankow JS, Kunze S, Shah SH, McRae AF, Lohman K, Sha J, Absher DM, Ferrucci L, Zhao W, Demerath EW, Bressler J, Grove ML, Huan T, Liu C, Mendelson MM, Yao C, Kiel DP, Peters A, Wang-Sattler R, Visscher PM,

Wray NR, Starr JM, Ding J, Rodriguez CJ, Wareham NJ, Irvin MR, Zhi D, Barrdahl M, Vineis P, Ambatipudi S, Uitterlinden AG, Hofman A, Schwartz J, Colicino E, Hou L, Vokonas PS, Hernandez DG, Singleton AB, Bandinelli S, Turner ST, Ware EB, Smith AK, Klengel T, Binder EB, Psaty BM, Taylor KD, Gharib SA, Swenson BR, Liang L, DeMeo DL, O'Connor GT, Herceg Z, Ressler KJ, Conneely KN, Sotoodehnia N, Kardia SL, Melzer D, Baccarelli AA, van Meurs JB, Romieu I, Arnett DK, Ong KK, Liu Y, Waldenberger M, Deary IJ, Fornage M, Levy D, London SJ. Epigenetic Signatures of Cigarette Smoking. *Circ Cardiovasc Genet* 2016: 9(5): 436-447.
46. de Vries M, van der Plaat DA, Nedeljkovic I, Verkaik-Schakel RN, Kooistra W, Amin N, van Duijn CM, Brandsma CA, van Diemen CC, Vonk JM, Marike Boezen H. From blood to lung tissue: effect of cigarette smoke on DNA methylation and lung function. *Respiratory research* 2018: 19(1): 212.
47. Roadmap Epigenomics C, Kundaje A, Meuleman W, Ernst J, Bilenky M, Yen A, Heravi-Moussavi A, Kheradpour P, Zhang Z, Wang J, Ziller MJ, Amin V, Whitaker JW, Schultz MD, Ward LD, Sarkar A, Quon G, Sandstrom RS, Eaton ML, Wu YC, Pfenning AR, Wang X, Claussnitzer M, Liu Y, Coarfa C, Harris RA, Shoresh N, Epstein CB, Gjoneska E, Leung D, Xie W, Hawkins RD, Lister R, Hong C, Gascard P, Mungall AJ, Moore R, Chuah E, Tam A, Canfield TK, Hansen RS, Kaul R, Sabo PJ, Bansal MS, Carles A, Dixon JR, Farh KH, Feizi S, Karlic R, Kim AR, Kulkarni A, Li D, Lowdon R, Elliott G, Mercer TR, Neph SJ, Onuchic V, Polak P, Rajagopal N, Ray P, Sallari RC, Siebenthall KT, Sinnott-Armstrong NA, Stevens M, Thurman RE, Wu J, Zhang B, Zhou X, Beaudet AE, Boyer LA, De Jager PL, Farnham PJ, Fisher SJ, Haussler D, Jones SJ, Li W, Marra MA, McManus MT, Sunyaev S, Thomson JA, Tlsty TD, Tsai LH, Wang W, Waterland RA, Zhang MQ, Chadwick LH, Bernstein BE, Costello JF, Ecker JR, Hirst M, Meissner A, Milosavljevic A, Ren B, Stamatoyannopoulos JA, Wang T, Kellis M. Integrative analysis of 111 reference human epigenomes. *Nature* 2015: 518(7539): 317-330.
48. Gerstein MB, Kundaje A, Hariharan M, Landt SG, Yan KK, Cheng C, Mu XJ, Khurana E, Rozowsky J, Alexander R, Min R, Alves P, Abyzov A, Addleman N, Bhardwaj N, Boyle AP, Cayting P, Charos A, Chen DZ, Cheng Y, Clarke D, Eastman C, Euskirchen G, Frietze S, Fu Y, Gertz J, Grubert F, Harmanci A, Jain P, Kasowski M, Lacroute P, Leng JJ, Lian J, Monahan H, O'Geen H, Ouyang Z, Partridge EC, Patacsil D, Pauli F, Raha D, Ramirez L, Reddy TE, Reed B, Shi M, Slifer T, Wang J, Wu L, Yang X, Yip KY, Zilberman-Schapira G, Batzoglou S, Sidow A, Farnham PJ, Myers RM, Weissman SM, Snyder M. Architecture of the human regulatory network derived from ENCODE data. *Nature* 2012: 489(7414): 91-100.
49. Rao SS, Huntley MH, Durand NC, Stamenova EK, Bochkov ID, Robinson JT, Sanborn AL, Machol I, Omer AD, Lander ES, Aiden EL. A 3D map of the human genome at kilobase resolution reveals principles of chromatin looping. *Cell* 2014: 159(7): 1665-1680.
50. Kanehisa M, Furumichi M, Tanabe M, Sato Y, Morishima K. KEGG: new perspectives on genomes, pathways, diseases and drugs. *Nucleic acids research* 2017: 45(D1): D353-D361.
51. Gene Ontology C. Gene Ontology Consortium: going forward. *Nucleic acids research* 2015: 43(Database issue): D1049-1056.
52. Phipson B, Maksimovic J, Oshlack A. missMethyl: an R package for analyzing data from Illumina's HumanMethylation450 platform. *Bioinformatics* 2016: 32(2): 286-288.
53. Kramer A, Green J, Pollard J, Jr., Tugendreich S. Causal analysis approaches in Ingenuity Pathway Analysis. *Bioinformatics* 2014: 30(4): 523-530.
54. Charmpi K, Ycart B. Weighted Kolmogorov Smirnov testing: an alternative for Gene Set Enrichment Analysis. *Stat Appl Genet Mol Biol* 2015: 14(3): 279-293.
55. Subramanian A, Tamayo P, Mootha VK, Mukherjee S, Ebert BL, Gillette MA, Paulovich A, Pomeroy SL, Golub TR, Lander ES, Mesirov JP. Gene set enrichment analysis: a knowledge-based approach for interpreting genome-wide expression profiles. *Proceedings of the National Academy of Sciences of the United States of America* 2005: 102(43): 15545-15550.
56. Gaunt TR, Shihab HA, Hemani G, Min JL, Woodward G, Lyttleton O, Zheng J, Duggirala A, McArdle WL, Ho K, Ring SM, Evans DM, Davey Smith G, Relton CL. Systematic identification of genetic influences on methylation across the human life course. *Genome Biol* 2016: 17: 61.

57. Hemani G, Zheng J, Elsworth B, Wade KH, Haberland V, Baird D, Laurin C, Burgess S, Bowden J, Langdon R, Tan VY, Yarmolinsky J, Shihab HA, Timpson NJ, Evans DM, Relton C, Martin RM, Davey Smith G, Gaunt TR, Haycock PC. The MR-Base platform supports systematic causal inference across the human phenome. *Elife* 2018: 7.
58. Chen YA, Lemire M, Choufani S, Butcher DT, Grafodatskaya D, Zanke BW, Gallinger S, Hudson TJ, Weksberg R. Discovery of cross-reactive probes and polymorphic CpGs in the Illumina Infinium HumanMethylation450 microarray. *Epigenetics* 2013: 8(2): 203-209.
59. Tingley D. YT, Hirose K., Keele L., Imai K. Mediation: R package for causal mediation analysis. *Journal of Statistical Software* 2014: 59(5): 1-38.
